# Parallelization of Neural Processing on Neuromorphic Hardware

**DOI:** 10.3389/fnins.2022.867027

**Published:** 2022-05-10

**Authors:** Luca Peres, Oliver Rhodes

**Affiliations:** Advanced Processor Technologies Group, Department of Computer Science, The University of Manchester, Manchester, United Kingdom

**Keywords:** neuromorphic computing, SpiNNaker, real-time, parallel programming, event-driven simulation, spiking neural networks

## Abstract

Learning and development in real brains typically happens over long timescales, making long-term exploration of these features a significant research challenge. One way to address this problem is to use computational models to explore the brain, with Spiking Neural Networks a popular choice to capture neuron and synapse dynamics. However, researchers require simulation tools and platforms to execute simulations in real- or sub-realtime, to enable exploration of features such as long-term learning and neural pathologies over meaningful periods. This article presents novel multicore processing strategies on the SpiNNaker Neuromorphic hardware, addressing parallelization of Spiking Neural Network operations through allocation of dedicated computational units to specific tasks (such as neural and synaptic processing) to optimize performance. The work advances previous real-time simulations of a cortical microcircuit model, parameterizing load balancing between computational units in order to explore trade-offs between computational complexity and speed, to provide the best fit for a given application. By exploiting the flexibility of the SpiNNaker Neuromorphic platform, up to 9× throughput of neural operations is demonstrated when running biologically representative Spiking Neural Networks.

## 1. Introduction

The human brain is capable of operating using less energy than a light bulb (Levy and Calvert, 2020). However, simulation of biologically representative Spiking Neural Networks (SNN) is a challenging task on conventional computer hardware. Models from the literature can produce millions of spikes per second, which need to be delivered to hundreds of thousands of neurons (Potjans and Diesmann, 2012; Schmidt et al., 2018; Casali et al., 2019) with very tight timing constraints. A common way to simulate these network dynamics is through CPU-based HPC platforms, using dedicated software such as NEST (Gewaltig and Diesmann, 2007). However, because of the timing constraints and the intrinsic high parallelism of these tasks, they fail to keep energy consumption low when attempting to run these applications, and performance gain is limited by the latency of MPI-based (Ippen et al., 2017) communications. An alternative approach, namely Neuromorphic engineering, inspired by the structure of the brain (Mead, 1990), has proven effective when dealing with this type of simulation (Rhodes et al., 2019), efficiently addressing the sparsity of signals typical of these applications and keeping energy consumption low. This approach is characterized by simple computational units with close access to distributed memory (Mead, 1990; Indiveri et al., 2011). To date, several Neuromorphic platforms have been developed, both in the digital, analog and mixed signal domains (Furber et al., 2014; Akopyan et al., 2015; Schemmel et al., 2017; Davies et al., 2018; Moradi et al., 2018). From a digital perspective, neurons (or neural compartments) are implemented by processors, which usually simulate both the neural dynamics and the synaptic receptors. Analog platforms on the other hand, employ a circuit implementation of models from literature. The efficiency of such systems is usually measured in terms of synaptic events (namely one spike targetting one synapse) per second and neurons they can simulate, with these two measures limited by the on-core memory capacity and computational power in digital neuromorphic platforms and by the physical implementation for analog architectures.

High synaptic fan-in represents one of the biggest challenges in biologically representative SNNs and it usually prevents real-time execution, requiring to slow down the simulations (i.e., resulting in a simulated time longer than the biological time) to process all network activity. Another strong limitation is given by long-range connections between different brain areas (Schmidt et al., 2018), which are typically represented by extremely sparse connectivity patterns. Recent work (Rhodes et al., 2019) demonstrated that, by performing more efficient task-partitioning and by acting on the placement of networks on Neuromorphic hardware, it is possible to improve significantly the throughput of these systems, enabling real-time execution of models that were not possible before.

Real-time simulations of biologically-representative SNNs are a common target in the field. Several solutions have been proposed to address the presented issues, including a procedural generation of the synaptic weights whenever a spike is received, instead of storing these, to reduce the memory footprint and improve performance (Knight and Nowotny, 2021). Some digital simulation platforms managed to achieve remarkable results in terms of real-time simulations, even reaching sub real-time performance for established benchmarks in the field (Knight et al., 2021; Kurth et al., 2021; Heittmann et al., 2022).

This work offers an improved parallelization strategy, namely the Multi-target partitioning, on how to efficiently deploy Spiking Neural Networks on Neuromorphic hardware. This strategy aims at addressing the major bottlenecks of SNN simulations and informing the design of the next generation of Neuromorphic platforms. The use-case platform chosen for this work is SpiNNaker, a many-core digital Neuromorphic platform designed at The University of Manchester (Furber et al., 2014).

Following this introduction, Section 2 provides a background on SNNs simulations in general, together with the critical aspects of real-time simulations and their challenges. Section 3 gives details about the SpiNNaker Neuromorphic platform and how SNNs are mapped on it through the available partitioning strategies. The Multi-target partitioning approach is then presented in Section 3.4. Section 4 demonstrates the advantages of this new strategy through benchmarking on SpiNNaker. Finally, Section 5 contains a discussion about the potentialities of this approach and possible future applications.

## 2. Background

### 2.1. Neural Processing

SNN simulations are typically performed starting from a high level description of the network characteristics, through high level specification languages such as PyNN (Davison et al., 2009). Groups of neurons sharing the same properties are grouped into ensembles called Populations, and the connections between them are called Projections. Starting from these high level descriptions, Populations and Projections are typically fragmented (or partitioned) such that they can fit the requirements set by the underlying hardware platform. Digital platforms commonly employ a discrete time resolution, using fixed length timesteps, within which all spikes are considered to happen at the same time. Each computational unit involved in the simulation is in charge of handling a subset of a Population, meaning that it needs to update the state of a predefined number of neurons, generate output spikes for those neurons and receive input spikes.

**Figure 1:**
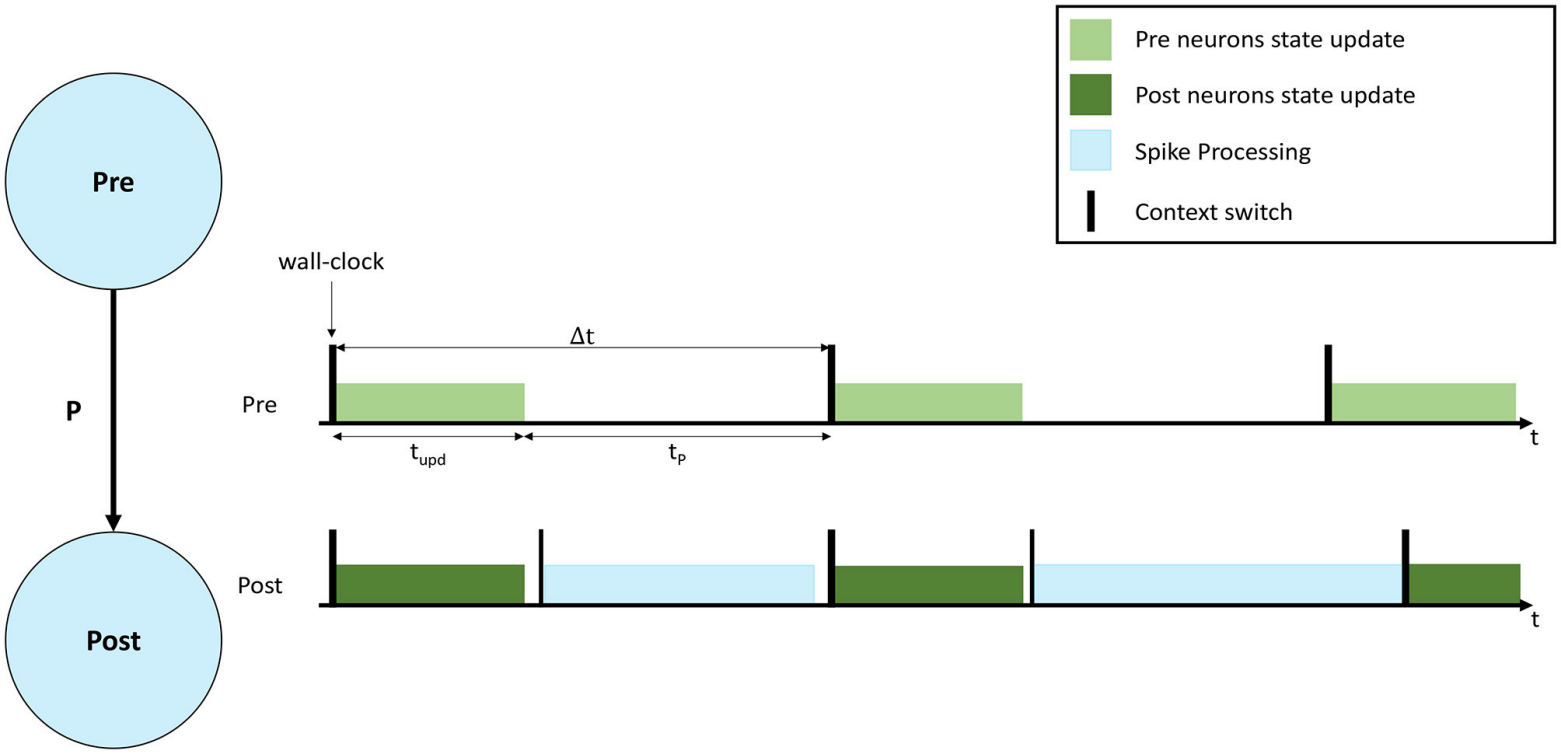
Representation of neural processing. The schematic of a SNN composed of 2 Populations (Pre and Post) with connectivity *P* is shown on the **left**. On the **right** the interaction of simulation events for 3 simulation timesteps, with real-time requirements violation is presented. The green bars show the neural state update, the blue bars the synaptic input processing.

A representation of a neural simulation is shown in Figure 1. Two Populations are shown (left), called Pre and Post, respectively, and the neurons are connected with a probability *P*, meaning that each presynaptic neuron has a probability *P* to connect to each postsynaptic neuron. An interaction of simulation events is shown on the right, where 3 simulation timesteps are presented for both Populations. In this case each Population is simulated by a separate computational unit. Timesteps are indicated by Δ*t*, and are synchronized among the involved computational units. Both computational units update the neural state for their implemented neurons according to the neuron model equations (light and dark green, respectively, in Figure 1). After the state update, neurons will fire, generating spikes that will be delivered to the Post neurons. The remaining fraction of the timestep (*t*_*P*_ in Figure 1) is commonly used to process the incoming spikes (light blue).

During a simulation, the length of the green bar (*t*_*upd*_) is constant. Increasing the number of neurons per computational unit extends the green bar. When the input firing activity is high (commonly when the number of input connections is high), or *P* is increased, the blue bar grows. The length of the blue bar therefore varies according to the amount of synaptic inputs received during the timestep. In order to maintain real-time processing, both the bars need to complete the execution before the beginning of the subsequent timestep (therefore before the next green bar is due to start). Figure 1 shows an example of a non real-time simulation, where the first timestep for the Post computational unit completes in time, however, during the second timestep the synaptic processing overflows on the third timestep, causing it to start delayed for the Post computational unit. Some platforms allow this case to happen, performing soft real-time simulations, and therefore allowing to overrun timesteps and then recover for the lost time in future timer periods, where the load is reduced. This however violates the hard real-time requirements, which mandate to simulate each individual timestep in the corresponding amount of wall-clock time: i.e., each 0.1 ms of biological time is completed in 0.1 ms.

A reduction of the size of the green bar (neural state update) can be achieved by reducing the number of neurons per computational unit. However, this operation has the effect of requiring additional hardware, since the network becomes more distributed and adds burdens to the communication fabric, increasing the number of destinations for the generated spikes. The time taken to process spikes, as indicated by the length of the blue bar in Figure 1, is a function of the number of postsynaptic neurons simulated per unit. When the number of neurons simulated per unit increases, each spike can potentially target more postsynaptic neurons, hence requiring more processing time. While the fan-in to each postsynaptic neuron is independent of the number of neurons simulated, the fan-out of each arriving spike is proportional to the number of available target neurons (defined by the number of neurons simulated per core). Therefore, when this number is reduced, the total available target neurons are reduced, meaning the cost of processing a spike is amortized over fewer individual connections. This reduction in efficiency is a significant problem, as spike processing tends to dominate computation in biologically-representative SNN simulations (Schmidt et al., 2018; Casali et al., 2019).

A more efficient partitioning strategy (Knight and Furber, 2016; Rhodes et al., 2019), demonstrated that it is possible to separate the two phases (neural state update and spike processing) onto separate computational units. This enables simulations with higher numbers of neurons per unit, together with higher efficiency for the synaptic input processing. This approach however still shows some limitations in dealing with very sparse connectivity patterns, as the number of target synapses per spike is still limited by the amount of neurons that can be simulated on a single computational unit. Section 3 presents a novel parallelization approach which overcomes this limitation, maximizing the number of postsynaptic receptors and improving spike processing performance.

## 3. Materials and Methods

### 3.1. The SpiNNaker System

SpiNNaker is a Globally Asynchronous Locally Synchronous (GALS) many-core digital Neuromorphic platform, specifically designed to simulate SNNs in real time (Plana et al., 2011; Furber et al., 2014). From a hardware perspective, its main building block is the SpiNNaker chip, which contains 18 ARM968 cores (ARM, 2006), each having two separate Tightly Coupled Memories (TCMs), to store local data and simulation code, respectively. Additionally the chip includes a 32 KB shared memory (SysRAM), a 128 MB off-chip shared memory (SDRAM) and a tree-based routing infrastructure which allows direct packet-based communication with 6 other neighboring chips. Each on-chip router can be used as an intermediate hop to forward packets to other chips (Furber et al., 2013; Painkras et al., 2013; Mavaridas et al., 2015). For fault tolerance purposes, the available cores per chip are 17. Access to the shared memories can be performed through bridge or Direct Memory Access (DMA). Bridge access is slow (> 100 ns/word), while a DMA controller provides more efficient bulk transfers (≈ 10 ns/word) up to 64 KB per request, with DMA requests broken down into bursts 128 B wide. Access to the memory controller is however limited to a single channel. Simultaneous attempts to access shared memory give rise to a phenomenon called contention, where a single requesting processor is given access to the memory controller and the others are queued (Painkras et al., 2013; Sharp and Furber, 2013; Rhodes et al., 2018).

SNNs models are simulated through dedicated software (Rhodes et al., 2018; Rowley et al., 2019), with each processor simulating a predefined number of neurons, each implemented through mathematical models governing their neural dynamics. All the available processors (excluding two service cores Rowley et al., 2019, used for system purposes) perform the simulation. This consists in updating the neural state of the implemented neurons in sequential fashion, generating postsynaptic action potentials where necessary, receiving incoming spikes and extracting the synaptic events from incoming packets. SNN simulations on SpiNNaker follow an event-driven approach (Sharp et al., 2011), where cores remain in a low-power state, until an event triggers a processing callback. Periodic timer events are used to advance the simulation time through discrete fixed-length timesteps, while asynchronous events signal the reception of a spike and trigger synaptic processing (Rhodes et al., 2018). Timesteps allow for discretization of continuous time models and, provided the timestep resolution is high enough (commonly 0.1 or 1 ms), allow modeling of neuron state updates *via* exponential integration (Rotter and Diesmann, 1999), calculating the dynamics timestep by timestep.

The spike processing activity spans through most of the simulation timestep and, in case of large networks (Potjans and Diesmann, 2012; Schmidt et al., 2018; Casali et al., 2019), the number of received spike events can cause the neural state update to be preempted and delayed beyond the boundaries of the simulation timesteps (van Albada et al., 2018; Bogdan et al., 2021), resulting in non real-time performance. Real-time performance means that the simulation time of a network matches the modeling time of the network itself, therefore 1 s of activity needs to be simulated in 1 s for it to be in biological real-time.

On SpiNNaker, spikes are delivered through multicast packets in the Address Event Representation (AER) format (Mead, 1989), therefore only containing information about the sender. All synaptic information for a given presynaptic spike (i.e., number of postsynaptic connections, weights and delays) is stored on the postsynaptic side in the SDRAM shared memory. This reduces the amount of information that is transmitted over the communication network, by only specifying the sender. Therefore, upon the reception of a spike packet, each core performs a DMA request to retrieve the associated synaptic data (Rhodes et al., 2018). This information is stored as a sparse synaptic matrix using the compressed-row format, row-indexed by the presynaptic neuron ID. Postsynaptic cores therefore, upon the reception of a spike have a unique identifier of the sender available (given by AER spike packets), and use this as an index to locate the correct synaptic row inside the matrix. By storing the synaptic matrices in the SDRAM memory it is possible to simulate SNNs where neurons have much larger individual fan-ins (a common aspect of biologically-representative SNNs). This overcomes the limitations set by reduced local memory typical of Neuromorphic platforms. This solution also allows simulations of plastic networks, as opposed to the procedural approach (Knight and Nowotny, 2021), and it is more suited to platforms where the memory access is faster than generating pseudo-random values, such as Neuromorphic hardware. This however comes with the penalty of retrieving synaptic rows every time a spike is received, and, in case of plastic networks, a write-back operation for the updated weights is required.

### 3.2. Homogeneous Parallelization

**Figure 2:**
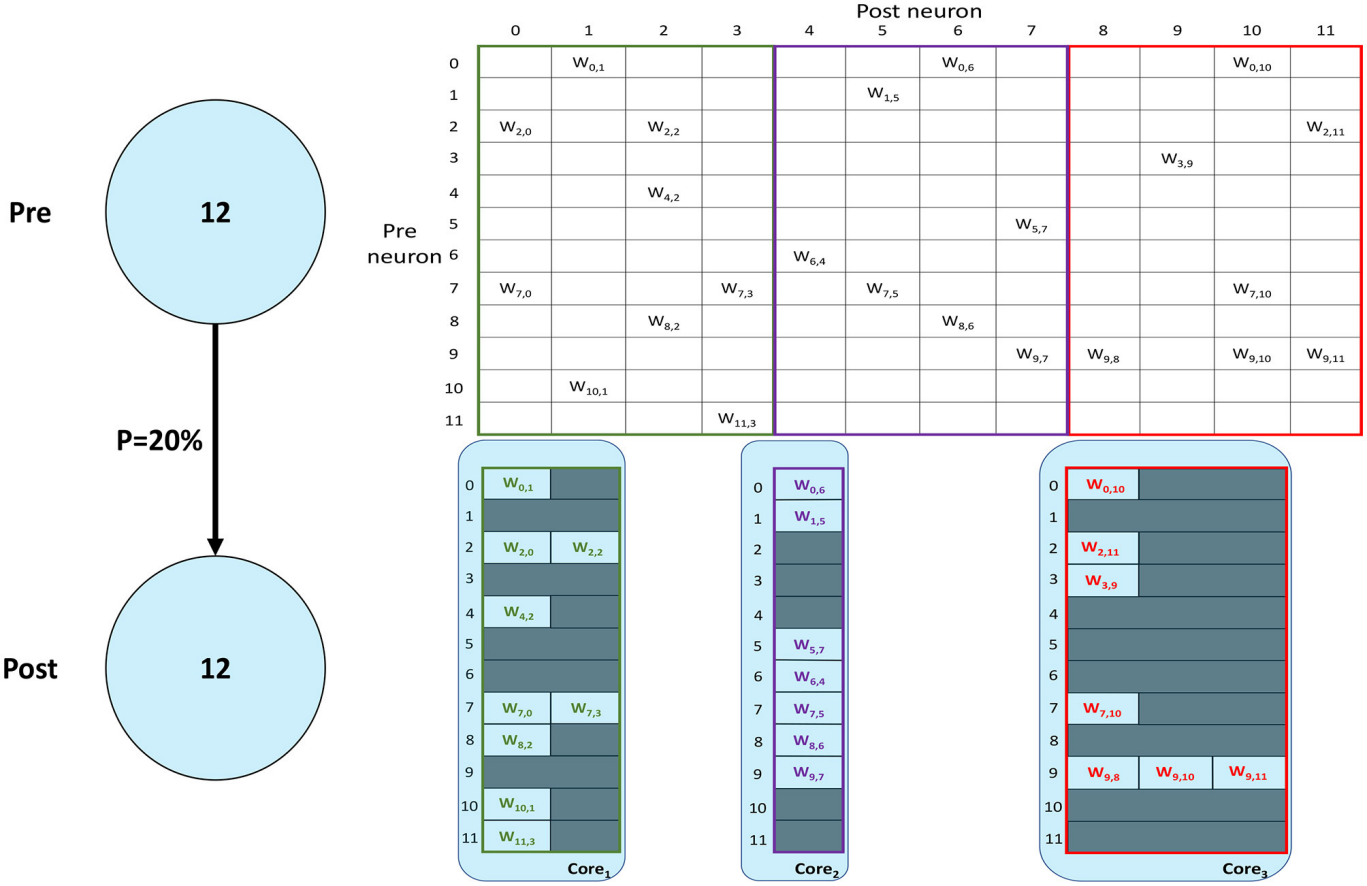
Synaptic matrix partitioning under the homogeneous partitioning. The presented matrix comes from an example network composed of 2 populations having 12 neurons each with 20% connectivity (schematic on the left). The full synaptic matrix is shown on top right. The sparse representation partitioned into 3 different cores is shown on bottom right (with colors matching the full synaptic matrix). The partitioning assumes a limit of 4 neurons per core, therefore 3 cores are required.

SNNs on SpiNNaker are commonly partitioned following a Homogeneous parallelization approach (Rhodes et al., 2018; Rowley et al., 2019), where each core simulates a subset of a Population, as described in Section 1. An example of the synaptic matrix representation under the Homogeneous parallelization approach is shown in Figure 2. Here, we show a network composed of 2 populations having 12 neurons each, connected with 20% probability (represented on the left). The full synaptic matrix is displayed (top right), where each row corresponds to a presynaptic neuron and each column to a postsynaptic neuron. Where a connection is formed a weight is added to the respective cell. Figure 2 shows how synaptic matrices are partitioned and mapped to SpiNNaker cores. The right bottom representation shows 3 cores each with its own sparse representation of the synaptic matrix, assuming a limit of 4 neurons per core. This representation reduces the size of the stored matrix, only including the relevant information.

Despite reducing the required memory to store synaptic matrices, this partitioning approach is inefficient; indeed, for sparse connectivity patterns it generates several empty rows, as seen in Figure 2. Each core has access to all the presynaptic rows pertaining to the implemented neurons. This limits the number of neurons that can be simulated per core, resulting in an inefficient allocation. Furthermore, for large networks, simply limiting the number of neurons per core is not sufficient, as the amount of incoming synaptic events requires a processing time larger than the timestep itself (van Albada et al., 2018; Bogdan et al., 2021). Also, by reducing the number of neurons per core, the length of the synaptic rows shrinks (as shown in Figure 2). This happens because SNNs typically have low connectivity probabilities, especially for long-range connections, therefore having a small number of postsynaptic neurons per core increases the chance of no connections being made, resulting in empty rows in the synaptic matrix. Empty rows are problematic because they cannot be detected until the core has completed the DMA transfer, resulting in wasted processing cycles retrieving meaningless information from SDRAM.

The cost of retrieving a synaptic row from shared memory is however amortized by the number of postsynaptic neurons implemented on each core, as a single transfer per packet is performed. This means that, by simulating more neurons per core it is possible to reduce the number of accesses to memory. A higher number of neurons per core however requires to process additional information, which might not be possible within the boundaries of the timestep.

Synaptic processing throughput is defined as the maximum number of synaptic events that can be processed per timestep, while maintaining real-time performance (Rhodes et al., 2018).


(1) $$\begin{array}{l} E=\left(\frac{t_{P}-t_{1^{st}}-t_{last}}{t_{spike}}+2\right)Pn \end{array}$$

(2) $$\begin{array}{l} t_{P}=\Delta t-t_{upd} \end{array}$$

(3) $$\begin{array}{l} t_{spike}=m_{s}Pn+c_{s} \end{array}$$

This can be evaluated according to Equations (1)–(3), where *E* represents the number of synaptic events per timestep, *t*_*P*_ indicates the fraction of the timestep available to process synaptic information, and is obtained by subtracting from the timestep duration (Δ*t*) the time required to update the neural state (*t*_*upd*_) of all the neurons simulated on core. The time required to process a single spike is defined by *t*_*spike*_. This value is expressed by Equation (3) and can be broken in a fixed contribution (*c*_*s*_), which is paid once per spike packet, corresponding to context switches, synaptic row location in the shared memory and transfer time, and a variable contribution (*m*_*s*_) which corresponds to the cost of processing a single synaptic event. Spike processing on SpiNNaker is handled through a pipelined approach, therefore the cost of processing the first and the last spike in the pipeline are different due to different API calls (Rhodes et al., 2018). These values are indicated by $t_{1^{st}}$ and *t*_*last*_, respectively, and follow the same rule as *t*_*spike*_, but have different values for fixed and variable costs (Rhodes et al., 2018).

The processing time ($t_{P}-t_{1^{st}}-t_{last}$) is divided by *t*_*spike*_ to obtain the processed spikes per timestep. This number is then incremented by 2, to account for $t_{1^{st}}$ and *t*_*last*_ previously subtracted. The number of synaptic events that can be processed in a single timestep is, therefore, given by multiplying the number of spikes by the connectivity probability (*P*), which indicates the number of postsynaptic connections per spike and then by the number of postsynaptic neurons on core (*n*).

### 3.3. Heterogeneous Parallelization

**Figure 3:**
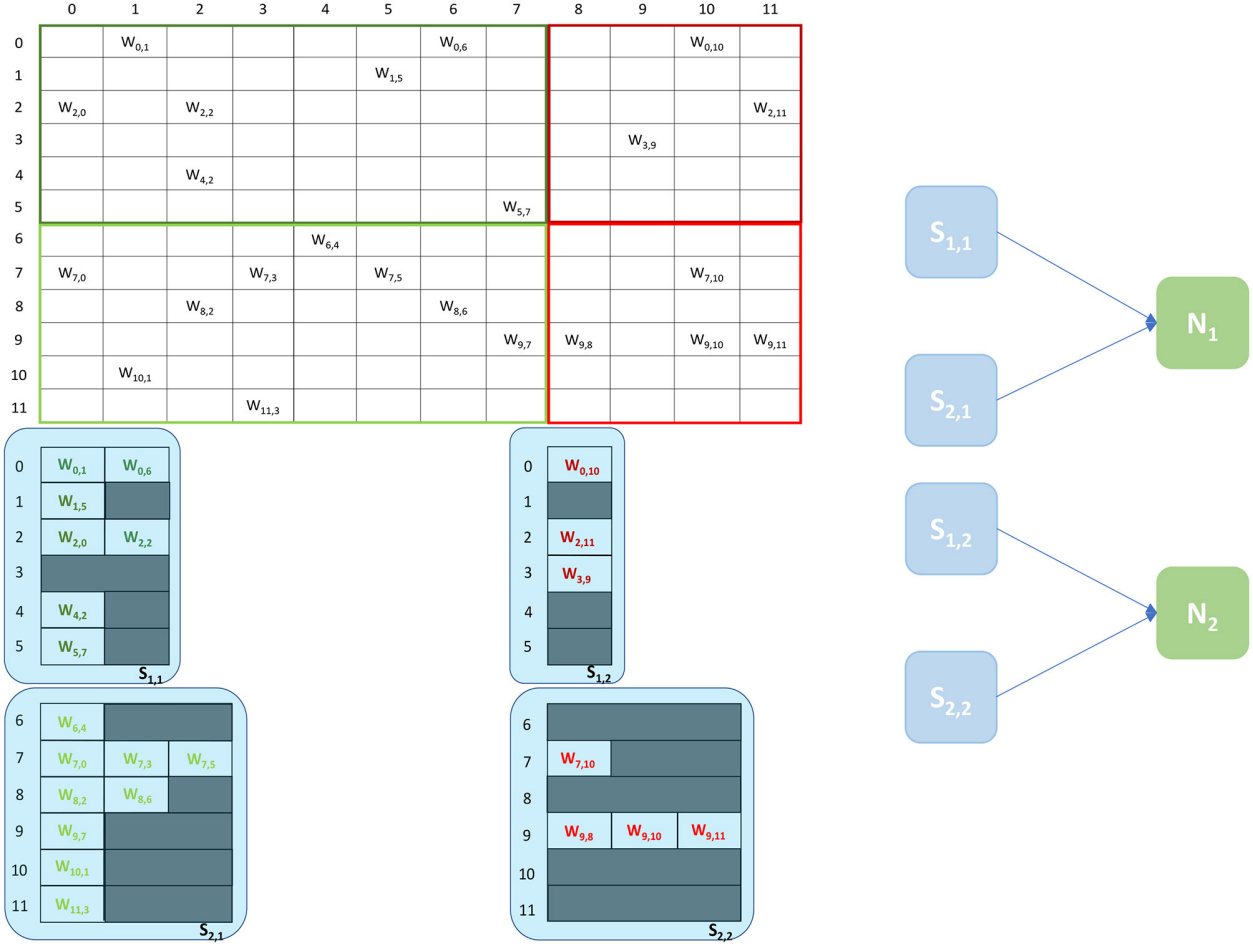
Synaptic matrix partitioning under the Heterogeneous Programming Model. The same matrix presented in Figure 2 is used. *Synapse* cores allow to partition the matrix by presynaptic index, and to relieve Neuron cores from processing spikes, enabling the possibility of simulating more neurons per core, which in turn allows to increase the length of synaptic rows. A schematic of the ensembles generated by this partitioning is shown on the right, where each *Neuron* core receives inputs from two *Synapse* cores.

The Heterogeneous Programming Model (Rhodes et al., 2019) is a simulation approach which evolved from a previous study on the partitioning of synaptic matrices on SpiNNaker (Knight and Furber, 2016). This approach aimed at improving the placement of SNNs on SpiNNaker to achieve real-time simulations of complex SNNs (Rhodes et al., 2019). By partitioning the synaptic matrices horizontally (see Figure 3), as opposed to the vertical approach (see Figure 2), it is possible to maintain longer postsynaptic rows and parallelize processing of incoming spikes. This is achieved by introducing separate cores, called *Synapse* cores, dedicated to the spike processing phase only, each implementing a subset of the synaptic receptors for each postsynaptic neuron (see Figure 3). The postsynaptic neurons are simulated on dedicated *Neuron* cores, having the role of advancing the neural state and generating action potentials only. These cores combine the inputs coming from the connected *Synapse* cores, through shared memory. This partitioning strategy allows simulations of higher numbers of neurons per core, therefore increasing the length of the synaptic rows maintained by the connected *Synapse* cores. This enables simulations of sparser connectivity patterns. Through this approach it is furthermore possible to connect multiple *Synapse* cores to each *Neuron* core, increasing the synaptic event throughput of the overall system (Knight and Furber, 2016; Rhodes et al., 2019). The communication between connected *Synapse* and *Neuron* cores happens *via* the chip-local SDRAM shared memory. Each *Synapse* core writes at the end of each timestep the synaptic contributions (representing partial input currents) coming from the receptors simulated by the core. The *Neuron* core reads all the contributions in a single memory block read and computes the total input currents by adding together the values from different *Synapse* cores.

An example of the partitioning of synaptic matrices under this approach is shown in Figure 3. In this example the same synaptic matrix addressed in Figure 2 is used, however it is now split horizontally by presynaptic neurons. Therefore, one *Synapse* core (*S*_11_) receives inputs from the lower 6 presynaptic neurons (dark green in Figure 2) and the other *Synapse* core (*S*_21_) from the higher 6 (light green in Figure 2). This increases the number of neurons per core, as the *Neuron* core's sole task is to update the neural state. In this simple example, each *Neuron* core can therefore now simulate 8 neurons, allowing to double the length of the synaptic rows associated to each *Synapse* core. The remaining 4 neurons are simulated by a separate *Neuron* core which replicates the structure of the other ensemble. The two ensembles are shown in Figure 3 right. *N*_1_ simulates the lower 8 postsynaptic neurons, *N*_2_ the remaining 4 neurons. Each *Neuron* core receives its inputs from 2 *Synapse* cores. The synaptic labels correspond to the cores depicted on the left.

The number of synaptic events that can be processed in a timestep under this approach per *Synapse* core is expressed by Equations (4)–(7), adapted from Equation (1). For this model, *t*_*p*_ represents the spike processing window, which is obtained by subtracting from the duration of the timestep (Δ*t*) the time required by the *Synapse* cores to write the synaptic contributions to shared memory (*t*_*w*_), minus the time taken by the postsynaptic *Neuron* core to read the contributions from shared memory (*t*_*r*_). These last two components represent a fraction of the timestep which is wasted, as during *t*_*w*_ no additional spikes can be processed, and during *t*_*r*_ the *Neuron* core has to wait, as it is retrieving the information necessary to update neuron state. The number of neurons is indicated by *n*. These are simulated by the *Neuron* core of the ensemble. The spike processing times *t*_*spike*_, $t_{1^{st}}$ and *t*_*last*_ follow the same rule presented in Equation (3).


(4) $$\begin{array}{l} E=\left[\frac{t_{p}-t_{1^{st}}-t_{last}}{t_{spike}}+2\right]Pn \end{array}$$

(5) $$\begin{array}{l} t_{p}=\Delta t-t_{w}-t_{r} \end{array}$$

(6) $$\begin{array}{l} t_{w}=aS_{c}+b \end{array}$$

(7) $$\begin{array}{l} t_{r}=cS_{c}+d \end{array}$$

**Figure 4:**
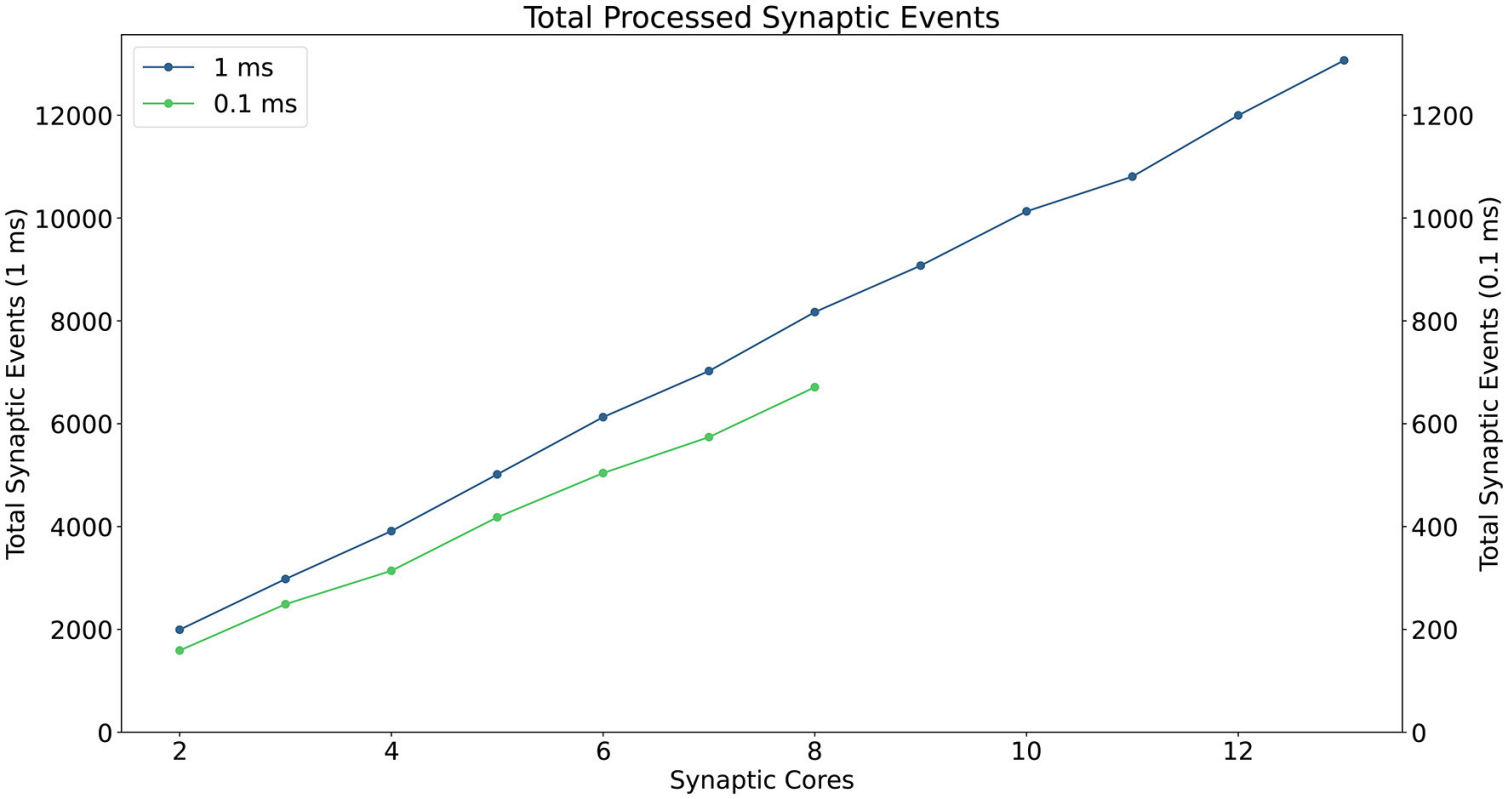
Processed synaptic events per timestep at 10% connectivity with increasing *Synapse* cores per ensemble. The blue line shows the 1 ms case (values reported on the left axis), the green line the 0.1 ms case (values reported on the right axis). The number of *Synapse* cores is limited to 8 for the latter, because of timing constraints due to the synaptic contributions reads.

A description of the read and write times is given by Equations (6) and (7) and they depend on the number of involved *Synapse* cores (*S*_*c*_). This dependency can be easily explained by the increase in size of the memory block containing the synaptic contributions (which size is directly proportional to the number of connected *Synapse* cores) to be read by the *Neuron* core every timestep, and by memory access contention, arising when multiple *Synapse* cores try to write to memory at the end of each timestep simultaneously. The lower case coefficients (*a, b, c*, and *d*) are hardware specific values. Previously measured quantities, obtained from experimental analysis on SpiNNaker, are shown in Table 1. The value described in Equation (4) represents the number of synaptic events per *Synapse* core. The total number of synaptic events per ensemble is calculated by adding together the values for each *Synapse* core belonging to the ensemble. Compared to the Homogeneous partitioning case, with the same number of postsynaptic neurons, this represents a pseudo-linear increase in the processed events per timestep. A demonstration of this can be seen in Figure 4, which shows the number of synaptic events processed by SpiNNaker for a 10% connectivity network, with increasing numbers of *Synapse* cores. Here, the blue line shows the 1 ms case and the green line 0.1 ms. For the latter it is not possible to include more than 8 *Synapse* cores per ensemble, as the synaptic contribution reading time from the *Neuron* core's perspective becomes predominant, therefore preventing real-time execution.

**Table 1:** Reading and writing time coefficients for the Heterogeneous and Multi-target partitioning measured on SpiNNaker.

**SpiNNaker reading and writing time coefficients**		
**Coefficient**	**Heterogeneous partitioning value**	**Multi-target partitioning value**
a	0.4	0.9
b	4	0.1
c	0.3	0.6
d	3.9	1.3
e	-	1.2
f	-	0.4
g	-	0.2

*Column 2 refers to Equations (6) and (7). Column 3 to Equations (10) and (11)*.

The Heterogeneous Programming model can achieve impressive performance improvements, however it also presents limitations, as seen in the example shown in Figure 3. The length of the synaptic rows is still not optimal, requiring two additional (or more, according to the presynaptic partitioning) *Synapse* cores (*S*_12_ and *S*_22_), to simulate the last four columns, resulting in additional resources being allocated and a sub-optimal partitioning of the matrices. Furthermore the number of synaptic events that can be processed for 0.1 ms timestep simulations is limited to the throughput of 8 *Synapse* cores, which does not allow to fully exploit the available parallelism.

### 3.4. Multi-Target *Synapse* Cores

Here, we present a novel parallelization approach enabling more efficient use of the available system resources, to address peak synaptic throughput performance and increased sparsity in synaptic connections.

This new approach, termed *Multi-target Partitioning*, extends the concept of *Synapse* cores introduced in Section 3.3, by assigning multiple *Neuron* core targets. Therefore, each neural ensemble will have multiple *Synapse* cores implementing the postsynaptic receptors of multiple *Neuron* cores, instead of matching the neurons of a single *Neuron* core. This technique improves partitioning of the synaptic matrices, by allowing longer rows. This therefore reduces the chance of empty rows for very sparse networks, and, at the same time, allows to amortize the fixed cost of processing a spike (i.e., preprocessing, context switches, and DMA cost) over a larger number of synapses.

**Figure 5:**
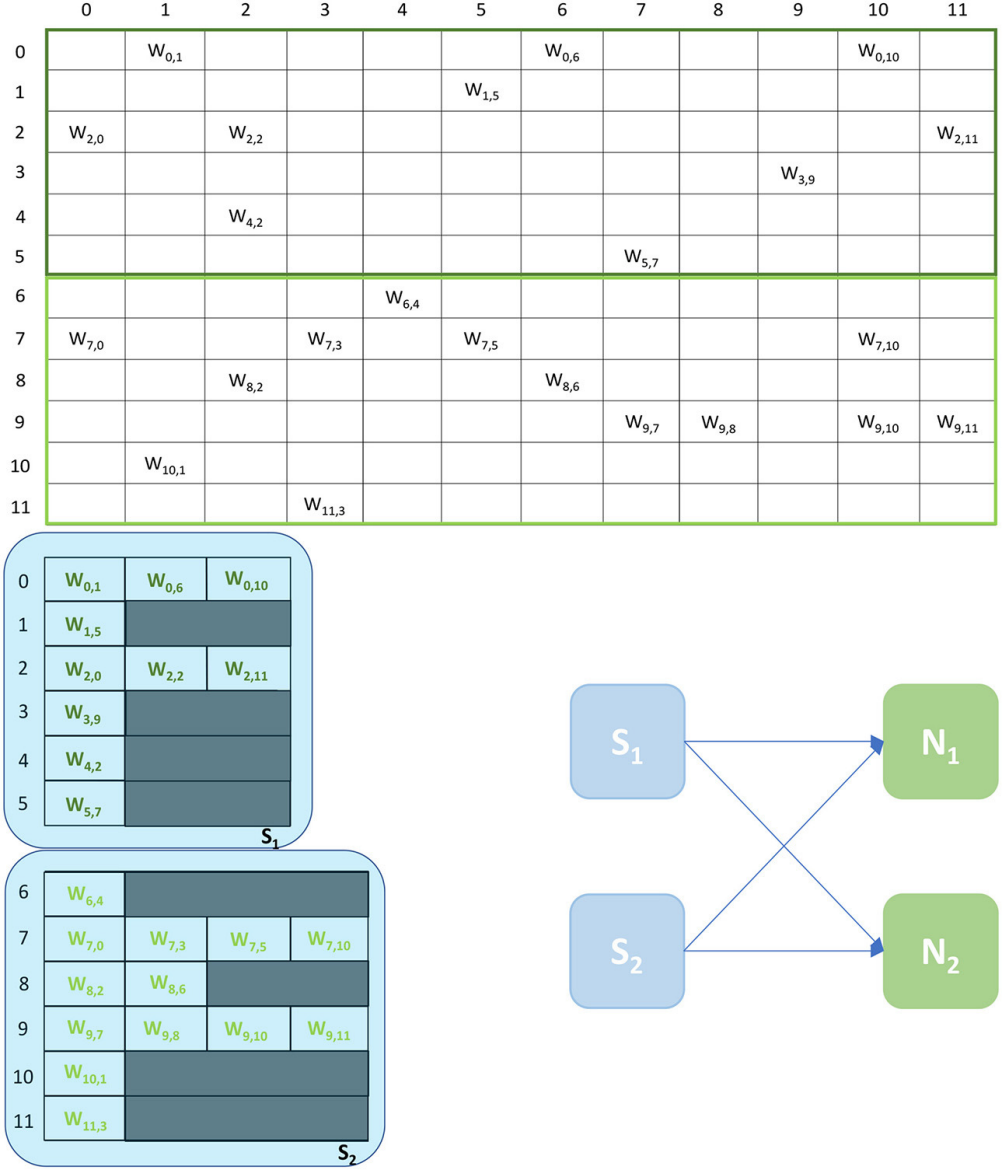
Synaptic matrix partitioning for the Multi-target approach. The used network is the same shown in Figures 2, 3. Here, *Synapse* cores have much longer synaptic rows, further reducing the risk of empty rows, therefore fewer resources are required. The generated ensemble is shown on the right.

An example of the Multi-target partitioning of synaptic matrices is shown in Figure 5. The *Synapse* cores now span over a much larger synaptic matrix, covering the entire rows in the example. The partitioning is performed presynaptically (horizontally), similarly to the Heterogeneous Model. However for the Multi-target partitioning, each *Synapse* core can target multiple postsynaptic *Neuron* cores, implementing all receptors for all target *Neuron* cores (effectively reducing the vertical partitioning). This approach allows to save resources (2 *Synapse* cores in the case of the example in Figure 5) and further reduces the chance of having empty rows for a given probability of connection. The number of synaptic events that can be processed per timestep is now modeled by Equations (8)–(13).


(8) $$\begin{array}{l} E=\left[\frac{t_{P}-t_{1^{st}}-t_{last}}{t_{spike}}+2\right]PN \end{array}$$

(9) $$\begin{array}{l} t_{p}=\Delta t-t_{w}-t_{r} \end{array}$$

(10) $$\begin{array}{l} t_{w}=aS_{c}-bN_{c}+cN_{c}S_{c}+d \end{array}$$

(11) $$\begin{array}{l} t_{r}=eS_{c}+fN_{c}-g \end{array}$$

(12) $$\begin{array}{l} N=nN_{c} \end{array}$$

(13) $$\begin{array}{l} t_{spike}=m_{s}PN+c_{s} \end{array}$$

The components are similar to the Heterogeneous model case, however *N* depends now on the number of *Neuron* cores connected to each *Synapse* core, and is obtained by multiplying the number of neurons per core (*n*) by the number of connected *Neuron* cores (*N*_*c*_). This reflects also on the spike processing times, as shown in Equation (13), where the variable cost is now multiplied by the total number of neurons targeted by the spike, therefore by the *Synapse* core. The reading (*t*_*r*_) and writing (*t*_*w*_) times now depend on the structure of the ensemble, as both contention and size of the transfer play a key role. The lower case coefficients (*a* to *g*) are hardware specific values, which therefore change according to the chosen platform. Table 1 reports values for the SpiNNaker platform obtained by profiling execution.

#### 3.4.1. Neuromorphic Implementation

**Figure 6:**
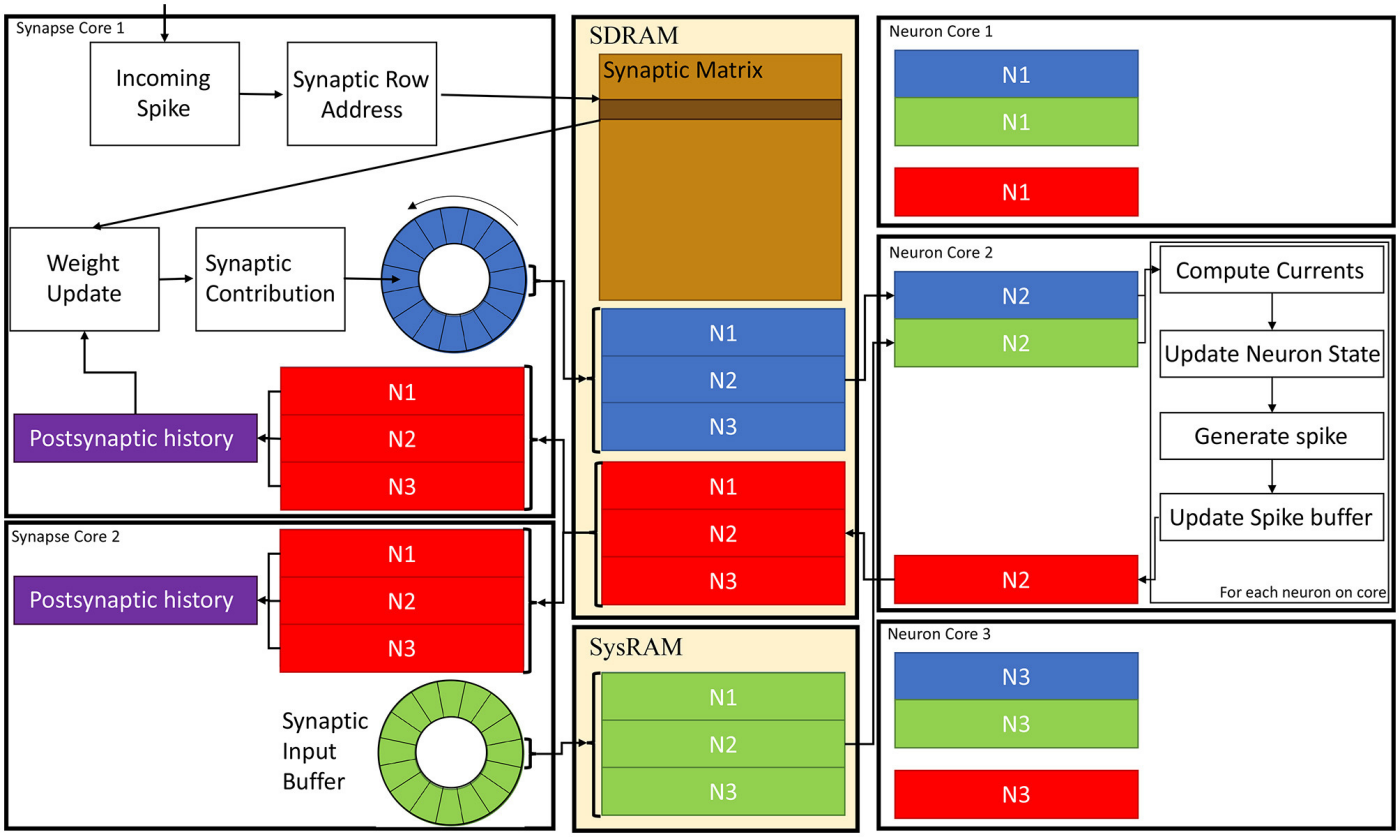
*Synapse* and *Neuron* cores memory interaction for the Multi-target partitioning. 2 *Synapse* cores targeting 3 *Neuron* cores are shown with all the steps from spike reception to neural state update. Communication between cores belonging to the same ensemble happens *via* the two shared memories (SDRAM and SysRAM), through the represented data structures.

A schematic of the core interactions and memory structures for the Multi-target partitioning implementation is shown in Figure 6. The ensemble demonstrates 2 *Synapse* cores each targeting 3 *Neuron* cores.

In the Multi-target approach the synaptic matrices are partitioned according to the synaptic view of the ensemble, meaning that the postsynaptic neurons simulated by multiple *Neuron* cores can now be included in a single matrix. Therefore, *Synapse* cores allocate the shared memory region for the current timestep synaptic contributions (blue and green blocks in SysRAM and SDRAM memories in Figure 6). This is opposed to the Heterogeneous Model, where the *Neuron* core of the ensemble sets the shared regions. This allows to perform a single block write per *Synapse* core per timestep, instead of fragmenting into multiple regions. This choice is motivated by architectural features, as the read throughput is higher than the write for the SpiNNaker chip (Painkras et al., 2013), therefore it is preferred to have fewer writes per timestep. *Neuron* cores retrieve the address of each memory block of each connected *Synapse* core, and compute the offset according to the indices of the implemented neurons (blue and green sub-blocks in Figure 6). This results in one write per *Synapse* core and multiple reads per *Neuron* core, according to the number of afferent *Synapse* cores.

During simulation initialization, *Synapse* core 1 allocates the blue region in SDRAM in Figure 6, which is large enough to store the contributions to postsynaptic neurons of all 3 *Neuron* cores. *Synapse* core 2 allocates the green region, having the same characteristics. The *Neuron* cores then retrieve the addresses of both memory regions and compute the starting address of their sub-regions according to the implemented postsynaptic neurons. Therefore, *Neuron* core 1 has the N1 sub-region from both the green and blue region, *Neuron* core 2 has the N2 sub-region and *Neuron* core 3 has N3. During a simulation timestep, when a spike is received, *Synapse* cores act the same way as the Heterogeneous Model (Rhodes et al., 2019). They extract the synaptic row address for the received spike, retrieve the correct row from the synaptic matrix and then add the connection weight to the synaptic input buffer (shown as circles in Figure 6 in blue for *Synapse* core 1 and in green for *Synapse* core 2), according to delay and postsynaptic index. Synaptic input buffers (Morrison et al., 2005; Rhodes et al., 2018) are structures employed to handle synaptic delays, and store the input currents for postsynaptic neurons. These are typically two-dimensional data structures, indexed by postsynaptic neuron ID and delay. When a spike is received on a postsynaptic core, for each postsynaptic neuron, the correct buffer slot is located, according to the delay and destination of the spike. Then, the weight of the connection is added to that buffer slot.At the end of the timestep, the slots of the synaptic input buffers representing the next timestep's synaptic input are written to shared memory (these include all the slots having delay 1 timestep). Therefore, *Synapse* core 1 writes N1, N2 and N3 sub-regions of the blue region, which will contain one slot per postsynaptic neuron having 1 timestep delay, and *Synapse* core 2 does the same for the green region. Different *Synapse* cores write to different memories (either SDRAM or SysRAM), to reduce contention on the SDRAM memory controller. The destination is decided according to the physical core ID,evenly spreading the contributions between the two memories. Both memories are part of the system memory map, therefore the allocation can be performed simply by specifying the correct memory heap, and the address retrieval is transparent to this operation.

At the beginning of the subsequent timestep, all *Neuron* cores perform reads of the sub-regions. Upon completion, the input currents for each postsynaptic neuron are calculated by adding together all contributions from the *Synapse* cores for the specific neuron. The synaptic currents are then used to update the neuron state, according to the implemented neuron model and, if the model mandates it, a spike is generated.

The time required to read the memory regions is a crucial design parameter, because it sets a boundary on when the *Neuron* core can generate the first spike. In fact until all the contributions are read, the *Neuron* cores cannot start processing the neural state updates. This reflects on when postsynaptic *Synapse* cores can start receiving spikes, effectively reducing the spike processing window. It is therefore of paramount importance to reduce this reading interval as much as possible. In order to address this issue, *Neuron* cores are instructed to perform out-of-order read operations of the sub-regions. This means that, based on the *Neuron* core ID, the first read region will be either from SysRAM or SDRAM. This effectively halves the *Neuron* cores accessing the same memory at the same time, by explicitly instructing half of them to first read from SDRAM and half of them from SysRAM. After each read is completed, each *Neuron* core sends the subsequent request to the other memory.

### 3.5. Plasticity

The Heterogeneous model and the Multi-target partitioning can be extended to include simulations of plastic SNNs. For plastic networks, the time required to process synaptic events is higher compared to the static case, as a weight update phase is needed. Therefore, simulations of plastic SNNs would also benefit from reduced processing time per synaptic event. Here, we present the steps to extend the plasticity framework, in order to include the Multi-target partitioning. This framework is independent from the implemented plasticity rule, and the synaptic update is fully handled by *Synapse* cores, which implement the chosen rule for a simulation. Figure 6 also shows the memory structures necessary for the implementation of STDP, as well as the weight update framework.

The plasticity framework adopted by the SpiNNaker toolchain performs synaptic weight updates upon receiving a spike (Galluppi et al., 2015). This minimizes the accesses to shared memory, as synaptic rows are commonly retrieved whenever a spike is received. After a row is stored in local memory, before adding the weight contribution to the correct synaptic input buffer, each weight is updated according to the implemented plasticity rule. STDP rules commonly require information about postsynaptic firing activity (Morrison et al., 2008). This information is stored into a postsynaptic buffer, locally maintained by the *Neuron* cores, which contains one slot per postsynaptic neuron, and is updated every time a neuron fires. The introduction of plasticity into the Multi-target approach adds complexity, since the *Neuron* cores need to communicate back to the *Synapse* cores which neurons have spiked during the timestep, to correctly update the synaptic weights. This operation is again performed through shared memory. All *Synapse* cores share the same postsynaptic region (red region in Figure 6), therefore this area is allocated into SDRAM by the *Synapse* core of the ensemble having the lowest index, and the address is retrieved by all the other *Synapse* cores. The *Neuron* cores get the address in the same way as the synaptic contributions, and will use the same offset to get access to their specific sub-regions.

During each timestep, after all the neurons on core have been updated, the postsynaptic buffer (red sub-blocks in the *Neuron* cores), which contains information on whether each neuron has spiked or not, is written to SDRAM by each *Neuron* core. The *Synapse* cores can read this region and update the postsynaptic history (purple buffers in Figure 6) for each receptor. In order to keep the memory operations short, the postsynaptic buffers are saved as binary flags, indicating whether each neuron has spiked or not. The update of the postsynaptic history depends on the simulated plasticity rule, which is implemented on the *Synapse* cores (as only these have visibility of the timing of incoming spikes). Only after this read operation is completed is it possible to update the weights and to process the incoming spikes. Therefore, the received spikes before this operation are buffered and ready to be processed when the read is completed. The *Synapse* core read is scheduled to happen after a fixed amount of time (for a given configuration), as the *Neuron* cores require a fixed amount of time to update the neural state and write back the postsynaptic buffers.

## 4. Results

The performance of the Multi-target partitioning approach presented in Section 3.4 is now evaluated from the perspectives of: system memory (Section 4.1), peak synaptic event throughput (Section 4.2) and the effect of connection sparsity (Section 4.3).

### 4.1. Memory Access

#### 4.1.1. Experiment Description

This first experiment measures the impact of writing and reading the synaptic contributions between *Synapse* and *Neuron* cores under the new ensembles scheme, showing timings for each possible combination of *Neuron* and *Synapse* cores on a chip. Each *Neuron* core is set to simulate 64 Leaky Integrate-and-Fire (Gerstner and Kistler, 2002) neurons, and afferent *Synapse* cores handle their synaptic receptors. In order to isolate the transfer times, the values are sampled in the context of a neural simulation in absence of spike packets. Therefore, the standard neural state is updated, but the spike processing pipeline and the spike generation phases are turned off. This prevents neural processing from increasing contention, while maintaining the characteristics required by SNN simulations. Each test simulates 100 timesteps, and is repeated 10 times to ensure consistency. For each arrangement timings are presented for both the SysRAM + SDRAM case, and the SDRAM only case. The results are presented in form of heatmaps, where the horizontal axis shows the number of employed *Synapse* cores, while the vertical axis the *Neuron* cores. All the *Synapse* cores for each case are connected to all the *Neuron* cores of the same case. The reported values are the worst case transfer times obtained by this test. These values are fundamental to estimate the impact of memory access time on the approach. Through these measurements it is possible to correctly allocate timings which allow the processors to initiate DMA transfers in time to maintain real-time performance.

#### 4.1.2. Reading Times

**Figure 7:**
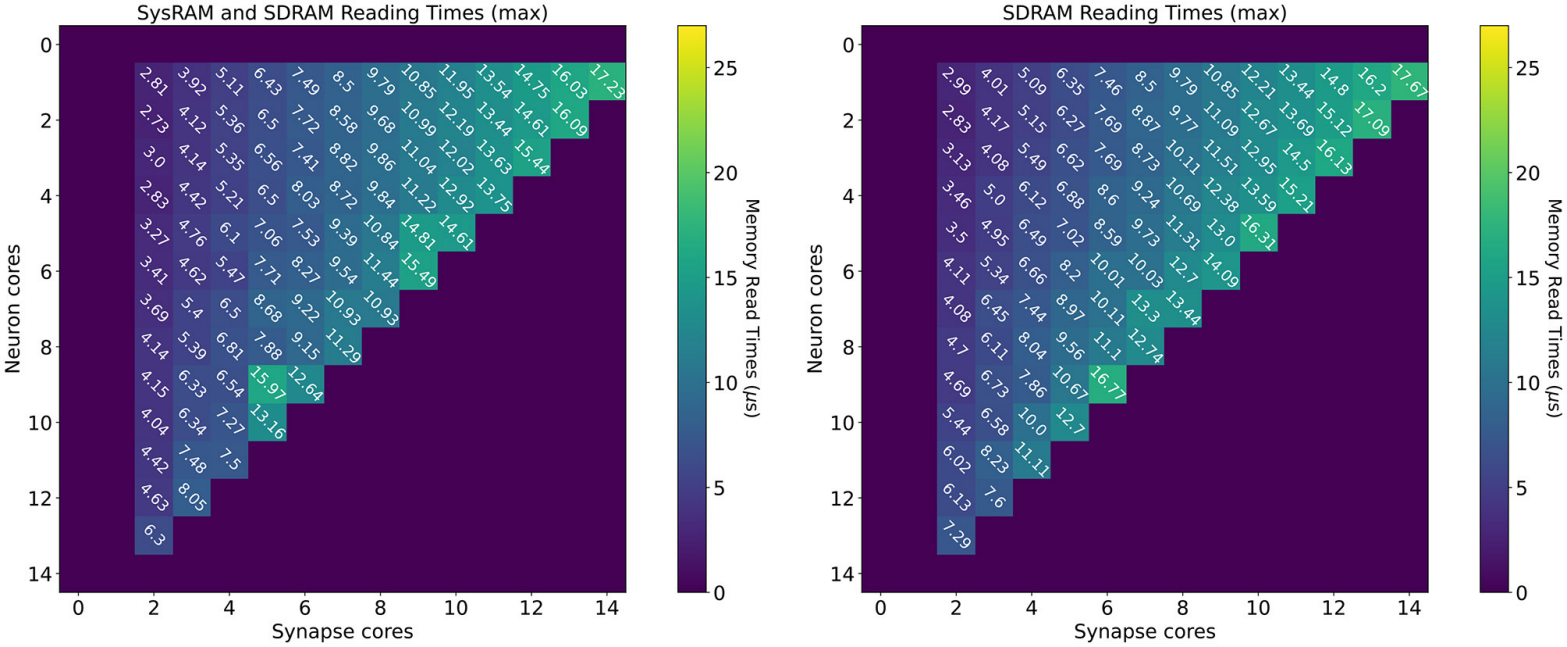
Memory heatmaps showing worst case DMA reading timings for increasing *Synapse* and *Neuron* cores. *Synapse* cores are represented on the horizontal axis, target *Neuron* cores on the vertical. All the measured times are in μs. The two plots represent the dual memory **(left)** and the SDRAM only case **(right)**. Purple blocks represent configurations not allowed by the machine.

Reading time measurements are shown in Figure 7 (all times measured in μs). The plot on the left presents values using both the shared memories available to the SpiNNaker chips (SysRAM and SDRAM), while the plot on the right contains timings relative to the SDRAM use only. All the purple boxes without a number are combinations of cores not allowed by the machine. The case with a single *Synapse* core has been omitted, since the transfer was completed quickly enough not to impact performance. The timings have been extracted in the context of a neural application simulating 64 neurons per core. Each synaptic weight is stored on a 16 bit (2 B) integer, meaning the contributions of a *Synapse* core targeting a single *Neuron* core amount to 64 × 2 B = 128 B (each DMA read has this fixed length).

By increasing the number of *Synapse* cores (moving from left to right on the horizontal axis), the number of reads per timestep per *Neuron* core increases. Reads are scheduled by the *Neuron* cores at the beginning of the timestep and performed sequentially, since there is a single DMA engine. As expected, for both the plots, the case with a single *Neuron* core (first line), shows linearly increasing reading times. The use of two separate memories does not influence this aspect, as one read at a time is performed. However it is observed that times in the dual memories plot are slightly lower. This is due to half of the *Synapse* cores contributions being stored into SysRAM which has a lower access time than SDRAM, therefore providing faster access. By increasing the number of *Neuron* cores (from top to bottom on the vertical axis), the contention increases, as multiple *Neuron* cores try to access shared memory to retrieve their synaptic contributions simultaneously. This case demonstrates the benefits of having two different memories in use with separate access. The SysRAM + SDRAM case indeed performs generally better than the single memory case allowing a gain up to 4 μs. There are, however, some isolated allocations where the single memory case performs better. This is probably due to a bad allocation of the cores on the chip, which results in a slower access to memory. Core allocation affects the memory access time, as to grant fairness, access to memory is regulated by a binary tree with arbiters at every junction point. The cores are on the leaves of the tree. A situation where the allocation of *Synapse* cores is unbalanced can cause higher contention between memory requests, as requests coming from more populated branches of the tree need to be filtered by multiple arbitration steps. This results in additional delays, which increase the total memory transfer time from the cores' perspective. Cores are assigned by the SpiNNaker toolchain during the placement phase. The values reported here represent the measured worst case reading times, therefore they are likely to represent the worst allocation of cores.

The worst case for both the experiments happens with 14 *Synapse* cores, which represents the placement with the highest number of sequential reads, performed by a single *Neuron* core. Furthermore, by keeping the number of *Synapse* cores constant, and increasing the *Neuron* cores, the transfer time becomes higher, as the reading contention increases. This reduces the portion of the timestep available for neural processing. It is therefore of paramount importance to understand the requirement of the SNN to be simulated, in order to determine the appropriate number of *Synapse* cores to allocate per *Neuron* core. It is noted that the values shown here represent the worst case scenario, thus presenting the highest recorded reading times. A more detailed analysis including best and average cases, is provided in the Supplementary Material.

The worst case analysis is important from a reading perspective to understand when the *Neuron* cores will start to fire, as the read phase must precede the neural state update and therefore *Neuron* cores must wait until this phase is completed before processing the neuron state update.

#### 4.1.3. Writing Times

**Figure 8:**
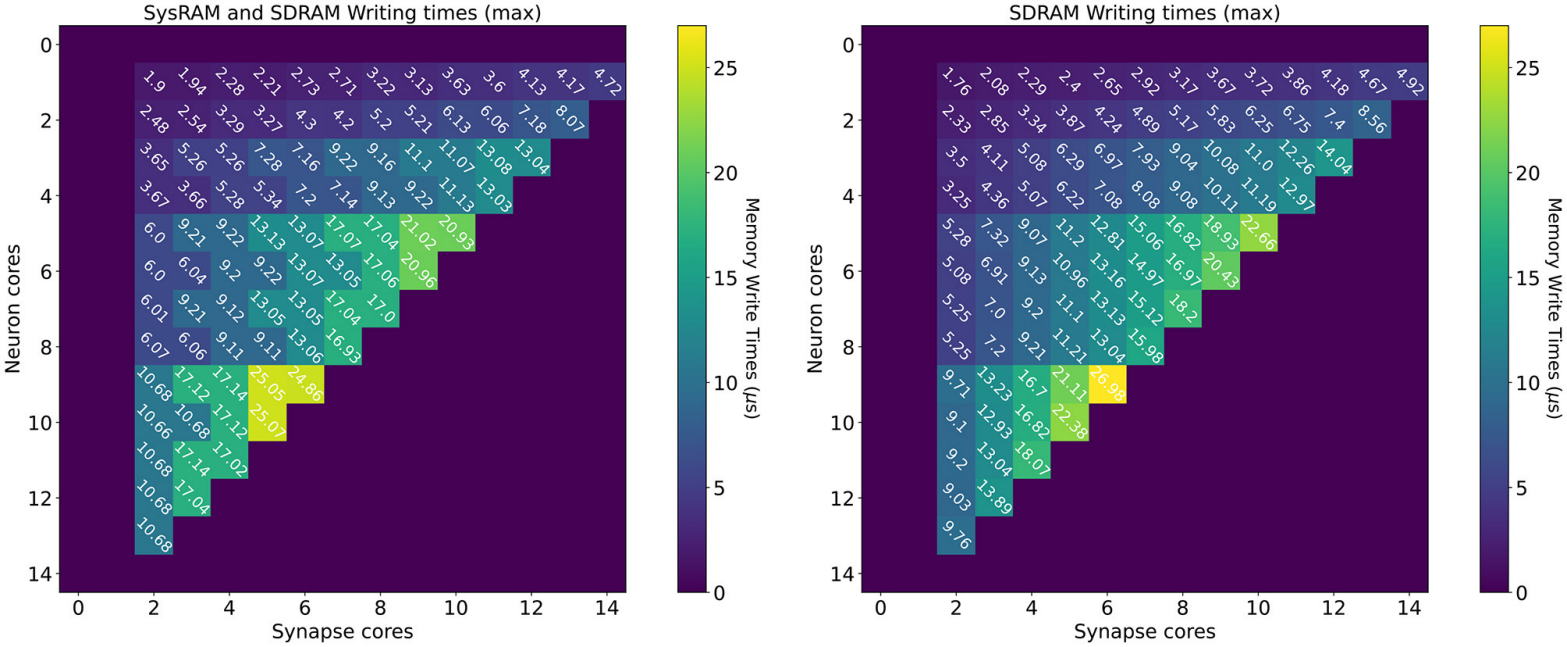
Memory heatmaps showing worst case DMA writing Timings for increasing *Synapse* and *Neuron* cores. *Synapse* cores are represented on the horizontal axis, target *Neuron* cores on the vertical. All the measured times are in μs. The two plots represent the dual memory **(left)** and the SDRAM only case **(right)**. Purple blocks represent configurations not allowed by the machine.

The measurements for the writing times are shown in Figure 8: the left plot shows the dual-memory case, while the right plot contains the SDRAM only case. Times are measured in μs, and each square represents a single write. Increasing *Synapse* cores are displayed horizontally, while increasing *Neuron* cores on the vertical axis. By increasing the number of *Synapse* cores, the contention grows, as multiple cores attempt to write to shared memory simultaneously. By increasing the number of *Neuron* cores however, the size of each write becomes larger. This is because each *Synapse* core performs one single write per timestep. Therefore, by increasing the number of postsynaptic receptors (connected *Neuron* cores), the number of synaptic contributions to be written grows as well. The size of each write is expressed by Equation (14), where *n* is the number of neurons per *Neuron* core (64 in this case), *w* is the size of a contribution (2 B for standard SNNs) and *T* is the number of target *Neuron* cores for each *Synapse* core. Therefore, in Figure 8, *T* increases vertically from top to bottom.


(14) $$\begin{array}{l} C=nwT \end{array}$$

Similarly to the read case, the reported times are the worst case measured writing times, and, for some cases, the access time is worse for the dual memory case. This can be due to several factors, as *Synapse* core contributions are partially located in SysRAM and partially in SDRAM. Although SysRAM provides a faster access, it has a slower transfer rate, therefore, for larger transfers, it can result in similar or worse performance compared to SDRAM. This, combined with a bad cores placement, can result in losing the advantages of using SysRAM, negating the faster memory access, due to contention on the memory controller. Average and best case measurements however highlight that this is an isolated case, and show that the dual memory approach is more effective for the arrangements of interest. For more details and analysis, please refer to the Supplementary Material.

From a writing perspective, the worst case scenario is useful to instruct *Synapse* cores on when to stop processing incoming spikes and start writing the synaptic contributions to shared memory (in order to meet real-time requirements). The highest recorded writing time is when using SDRAM only with 6 *Synapse* cores targeting 7 *Neuron* cores. This time amounts to 26.98 μs. This does not represent an issue in 1 ms timesteps simulations, but amounts to more than a quarter of the timestep for real-time simulations with 0.1 ms timesteps.

The worst case writing and reading measurements therefore allow to Taylor synaptic contribution writing and reading times to the required number of *Synapse* and *Neuron* cores per ensemble. This avoids overestimations which would further reduce the processing time shown in Equation (9). This analysis shows the importance of balancing the number of *Synapse* and *Neuron* cores according to the application requirements, in order to incur minimal memory access penalties. Network sparsity and firing activity also play a key role in the choice of core allocations, therefore the next sections focus on these aspects.

### 4.2. Peak Processing Profiling

#### 4.2.1. Experiment Description

The most useful metric when evaluating throughput performance of the Multi-target partitioning is the maximum number of processed synaptic events per timestep. This experiment therefore compares the peak throughput performance for the Multi-target partitioning to previous works. To perform a fair comparison, the same SNN is profiled using the different approaches: Multi-target and Heterogeneous models. The same number of cores is allocated for both configurations, but with different internal connections between *Synapse* cores and target *Neuron* cores. A third configuration is also presented, referred to as *single target expanded*. This consists of a standard Heterogeneous partitioning which maintains the same number of *Neuron* cores as the previous two cases, but allocates the same input *Synapse* cores capacity per *Neuron* core as the Multi-target approach. This last configuration provides a useful comparison, as the number of cores required for the single target Heterogeneous partitioning is adjusted to match the input capability of the Multi-target partitioning. The aim of including these cases is, therefore, twofold: first to compare the Multi-target partitioning to its Heterogeneous counterpart employing the same hardware resources, evaluating the performance difference; second, to show that, to achieve the input processing capability of the Multi-target approach, while using the Heterogeneous partitioning, is necessary to employ a larger number of hardware resources. This is represented by the *single target expanded* case.

**Figure 9:**
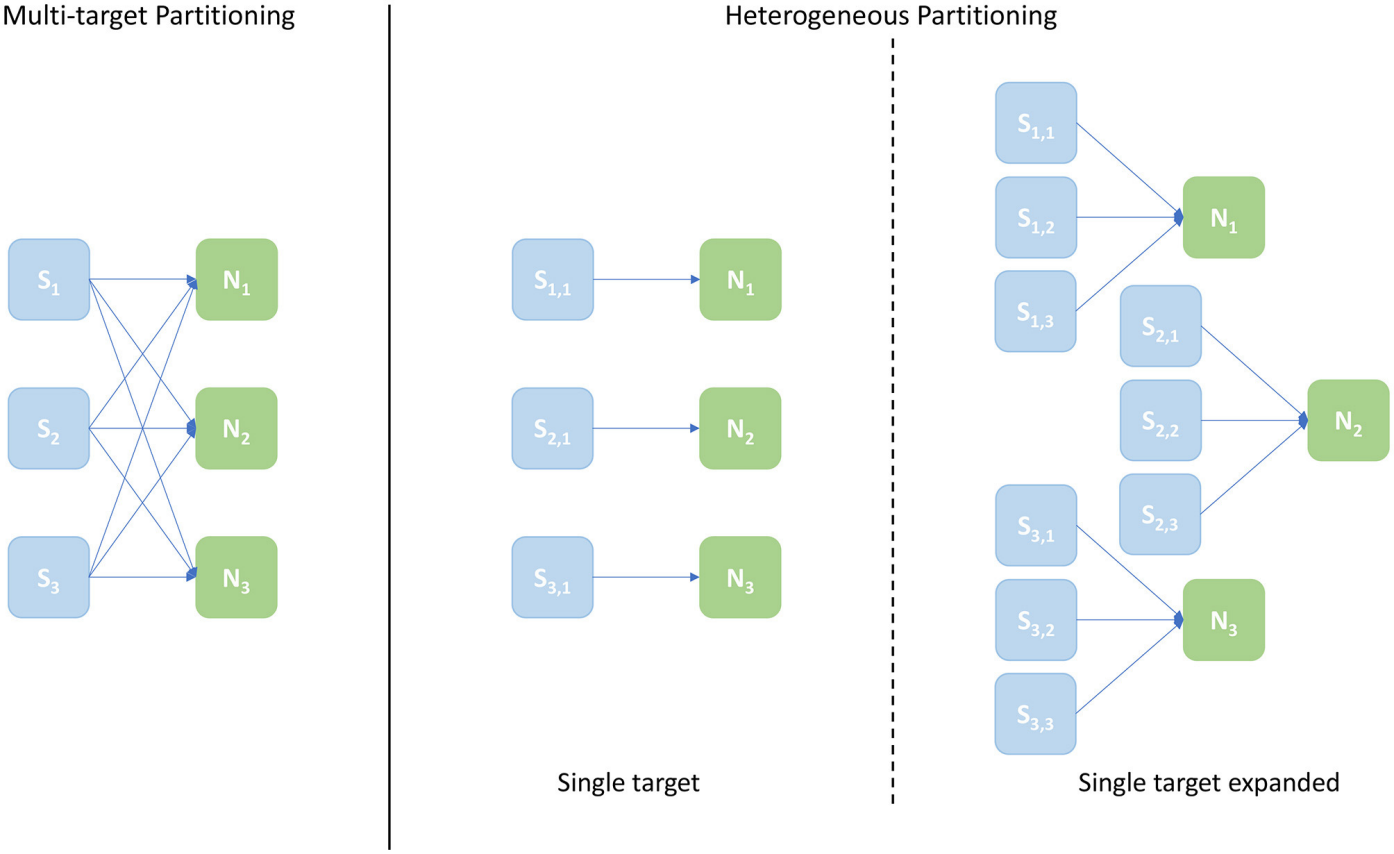
Arrangement of *Synapse* and *Neuron* cores under the explored configurations: Multi-target partitioning **(left)**; Heterogeneous partitioning **(right)**. The example shown demonstrates the [3, 3] test case, with 3 *Synapse* cores and 3 *Neuron* cores. For the Multi-target partitioning configuration, each *Synapse* core targets all *Neuron* cores. Comparison to the Heterogeneous approach is provided by: the Single-target partitioning, where the same overall number of cores are used, but connected one *Synapse* core to each *Neuron* core; and the Single-target expanded partitioning, where the same number of *Neuron* cores is maintained, but each with the same number of *Synapse* cores as implemented in the Multi-target approach.

A schematic of core allocations for the three approaches is shown in Figure 9. The experiments run to evaluate this metric are structured in test cases defined by 2 numbers in the form [*S*_*c*_, *N*_*c*_], where *S*_*c*_ is the number of *Synapse* cores and *N*_*c*_ the number of *Neuron* cores – the case shown in Figure 9 is [3, 3]. The Multi-target partitioning is shown on the left, where all the *Synapse* cores are connected to all the *Neuron* cores. The Heterogeneous partitioning is shown on the right, including the two different mappings explored: *single target* and *single target expanded*. The *single target* Heterogeneous partitioning presents 3 *Neuron* cores receiving input from a single *Synapse* core each, showing an input capacity reduced by a third compare to the Multi-target case. The *single target expanded* in the experiment is therefore comparable with the [3, 3] cases for the two other configurations, however the number of cores allocated is [9, 3]. This single-target expanded configuration matches the input capacity per *Neuron* core of the Multi-target partitioning, keeping the same number of neurons and *Neuron* cores (therefore in the presented example each *Neuron* core receives inputs from 3 *Synapse* cores similarly to the Multi-target case, but each *Synapse* core is single target). The intent here is to show that the Multi-target partitioning can reach similar performance compared to this extended configuration, requiring only a fraction of the allocated resources.

The SNN model used for this experiment consists of 2 populations of neurons, configurable with a range of sizes and connectivity (similar to that shown in Figure 1 left). All the presynaptic neurons are Leaky Integrate-and-Fire (Gerstner and Kistler, 2002) spiking neurons, with current-based exponentially-decaying synapses. Neurons are initialized with the internal voltage above firing threshold to produce spikes in a controlled manner. This approach is adopted to send spikes, instead of using spike sources, as it better represents the interaction between cores when simulating biologically-representative SNNs. This is because spike sources on SpiNNaker generate and send all spike packets together, causing a high firing activity concentrated at the beginning of the timestep, and then they remain silent. Cores implementing Populations (*Neuron* cores in this case) on the other hand, generate spike packets every time a neuron is updated and the model equations require it to spike, therefore distributing the spike packet generation over the timestep.

The size of the presynaptic Population changes according to the number of incoming partitions (number of *Synapse* cores per ensemble) of the postsynaptic Population. These Population sizes have been obtained experimentally, such that the postsynaptic Population receives more spike packets than it can process. This allows saturation of the receivers in order to determine their limits. The number of generated spike packets however needs to be limited, due to limitations set by the SpiNNaker communication infrastructure (Mavaridas et al., 2015). An excessive firing activity would cause higher congestion at the routing level, causing spike packets to be delivered late. This would result in lower processed synaptic events, compared to the real peak throughput, due to late arrivals. More details about Population sizes can be found in the Supplementary Material. The postsynaptic Population employs the same type of neurons as the presynaptic Population, and has variable size between 64 to 896 neurons (corresponding to 1–14 *Neuron* cores, respectively). Different connectivity patterns have been tested to demonstrate the robustness of the approach. Here, the 1% connectivity case is shown, as it is commonly found in biologically-representative SNNs (Potjans and Diesmann, 2012; Schmidt et al., 2018). For 0.1, 5, and 10% connectivities, please refer to the Supplementary Material.

The same experiment was run both with 1 and 0.1 ms timesteps. The importance of showing results with both timestep resolutions is given by the requirement of biologically-representative SNNs to be modeled using tighter timing resolutions, to better capture their dynamics. Real-time 0.1 ms timestep simulations, indeed, present additional challenges due to tighter timing constraints and a reduced spike processing window (as demonstrated in Section 4.1), which is not amortized by a smaller number of neurons or synaptic receptors.

The simulated network in this experiment was the same for both 1 and 0.1 ms timestep cases, with the exception of the presynaptic Population size, which was scaled down of a factor ≈10× (see Supplementary Material for exact values). The same experiment was run both for plastic and static networks and the results are presented separately. In order to provide a fair comparison the number of neurons per core is kept fixed at 64. For additional cases, please refer to the Supplementary Material.

#### 4.2.2. Static Networks

**Figure 10:**
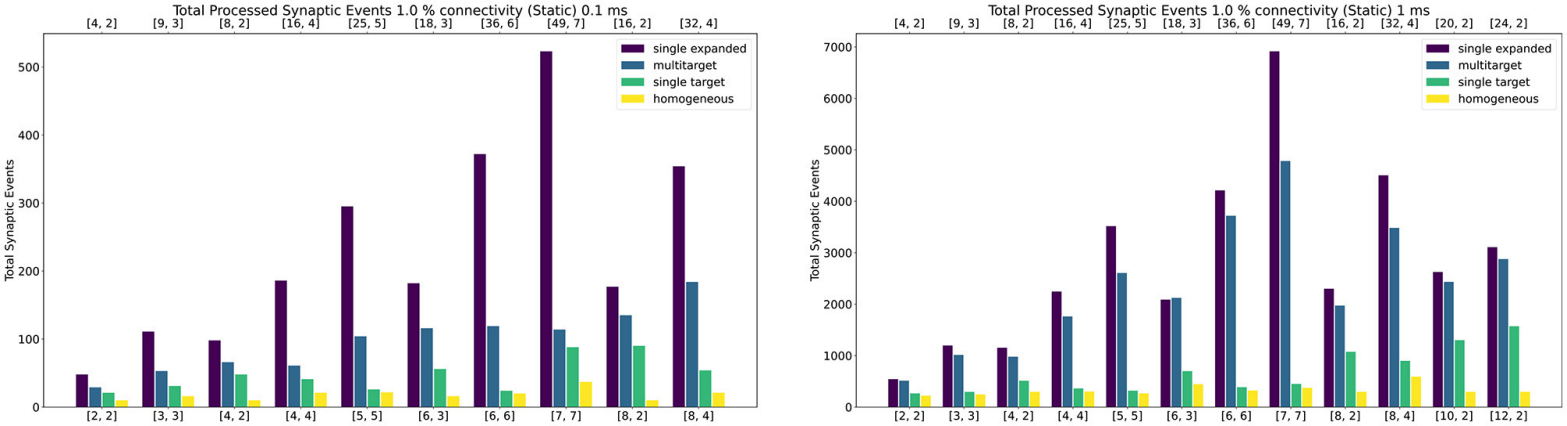
Peak processed synaptic events per timestep. The presented configuration represents a 1% connectivity static network. Both 0.1 ms **(left)** and 1 ms **(right)** cases are shown. Each plot contains the results for the Single target expanded (purple), multi-target (blue), single target (green) and baseline homogeneous partitioning (yellow) cases. The horizontal axes show the number of cores per ensemble in the form of [*S*_*c*_, *N*_*c*_], as indicated in Section 4.2.1 and Figure 9. The top axis refers to the single target expanded case (purple), the bottom to the other cases.

Figure 10 shows peak synaptic event throughput in the form of barcharts for the experiment with static connections, for both 0.1 ms (left) and 1 ms (right) timesteps. The connectivity between the two Populations is randomly generated with a probability of a connection between a pre- and postsynaptic neuron set to 1%. The *Multi-target* case is represented by the blue bars, while the *single target* with the same amount of cores by the green bars. The purple bars represent the *single target expanded* case. Finally, the yellow bars show the processed synaptic events using the Homogeneous partitioning with the same network and neurons per core.

Both the single target cases (green and purple) make use of the Heterogeneous model. The number of employed cores for each test case is indicated on the horizontal axes. The lower axis refers to the *Multi-target* (blue) and the *single target* (green). The upper axis shows values for the *single target expanded* (purple). The chosen configurations of cores allow direct comparison of the approaches. The left number in each tuple represents the *Synapse* cores of that test case, the right number the *Neuron* cores (as shown by the example presented in Figure 9). In the case of the Multi-target partitioning, all the *Synapse* cores of the ensemble target all the *Neuron* cores. For the single target cases the number of *Synapse* cores per *Neuron* core is obtained dividing the first number by the second. The blue and green bars are on the same axis because they employ the same number of cores, the difference between these two cases is in the connections between cores. This demonstrates that it is possible to improve the peak processing by rearranging the available units. The purple cases use the same number of *Synapse* cores per ensemble of the green tests, however, in this case each *Synapse* core has one single target (therefore there is a single *Neuron* core per ensemble). This replicates the input capabilities of the Multi-target partitioning per ensemble, but requires a considerably higher amounts of cores compared to the Multi-target case, resulting in the worst case of 56 total cores compared to 14 (8^*th*^ test case).

In all the cases the *Multi-target* approach (blue) performs better compared to the *single target* model (green). This is because the Multi-target partitioning performs a more efficient use of the available system resources compared to the Heterogeneous partitioning, allocating a higher input processing capacity to each *Neuron* core.

For the 1 ms timestep experiment the highest synaptic event throughput is given by the [7,7] configuration, where the Multi-target partitioning processes ≈9× more synaptic events than the heterogeneous partitioning. The reason why this happens is due to a full exploitation of the source-based partitioning offered by the approach. Each *Synapse* core in the *Multi-target* case receives inputs from one seventh of the presynaptic neurons and targets all the 448 postsynaptic neurons. The *single target* partitioning on the other hand, has each *Synapse* core receiving inputs from all the presynaptic neurons, but targets only 64 neurons. Because the connectivity is very sparse, a reduced input traffic achieves better results.

The *Multi-target* approach performs well also compared to the *single target expanded* (purple), which represents a remarkable result, since the amount of resources in use is much lower, especially in the [7, 7] case. The single target expanded approach employs the same number of *Synapse* cores per ensemble as the Multi-target partitioning, but has a single target per ensemble. Therefore, in the [7, 7] case ([49, 7] for the *single target expanded*), each *Synapse* core receives input from one seventh of the presynaptic neurons and targets 64 postsynaptic neurons only.

The trend is similar for 0.1 ms timesteps, with the *Multi-target* partitioning performing better than the *single target* case. However, with higher numbers of *Synapse* cores targeting higher numbers of *Neuron* cores, performance compared to the *single target expanded* case tends to be lower. This is due to the tight constraints set by the timestep resolution and the fact that memory read and write times for the synaptic contributions do not scale down with the timestep resolution.

This experiment shows that, by efficiently using the Multi-target partitioning, it is possible to achieve comparable results to the *single target expanded* case, but with a fraction of the hardware resources (a quarter in the [7, 7] case). Furthermore, with the same amount of resources it is possible to achieve considerably higher synaptic event throughput.

**Figure 11:**
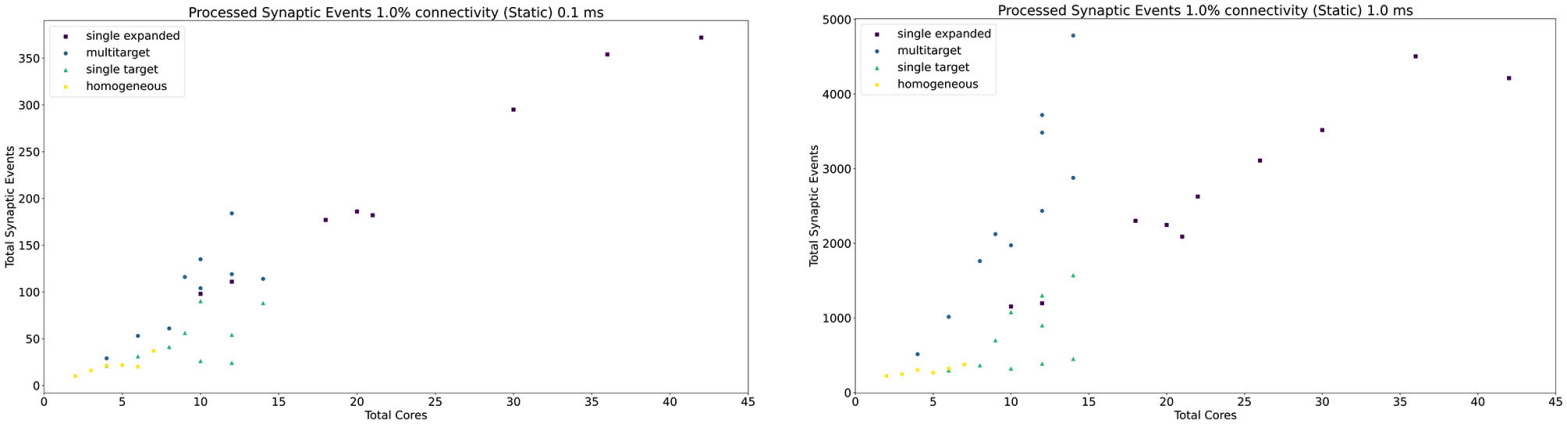
Resource allocation vs. peak performance for the different partitioning strategies (*single target expanded, multi-target, single target*, and baseline homogeneous). The network is the same used for Figure 10, with 1% connectivity and static connections. The color scheme matches that used in Figure 10. For a case by case labeled version of this plot, please refer to the Supplementary Material.

The general trend for the three approaches, together with the Homogeneous partitioning baseline is compared in Figure 11, where the horizontal axis shows the total number of allocated cores, and the vertical axis the processed synaptic events per timestep. The simulations are analogous to those shown in Figure 10. Each point in Figure 11 matches one of the bars (refer to the Supplementary Material for a case by case labeled representation of this plot). The *Multi-target* approach shows the best gain, having the steepest increase compared to the other three approaches, performing the best use of the available resources. Additional analysis is performed in the Supplementary Material including 0.1, 5, and 10% connectivity patterns for both the 0.1 and 1 ms timestep resolutions.

#### 4.2.3. Plastic Networks

**Figure 12:**
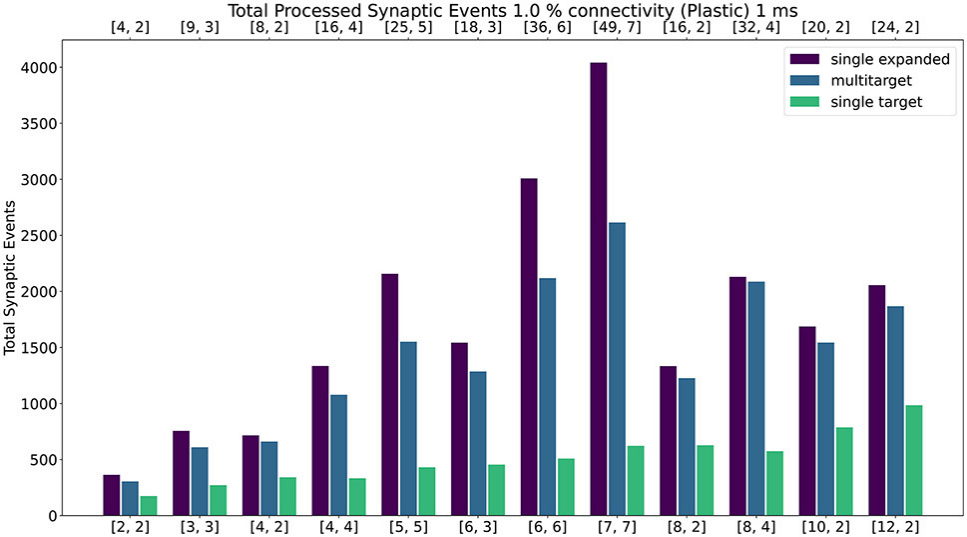
Peak processed synaptic events per timestep. The presented configuration represents a 1% connectivity network with plastic connections. Timestep resolution is set to 1 ms. The plot shows results for the *single target expanded* (purple), *multi-target* (blue) and *single target* (green) cases. The horizontal axes show the number of cores per ensemble in the form of [[*S*_*c*_, *N*_*c*_]], as indicated in Section 4.2.1 and Figure 9. The **top** axis refers to the single target expanded case (purple), the **bottom** to the other cases.

Figure 12 shows the results of the experiment with the addition of synaptic plasticity. The color scheme for the bar chart is analogous to the static case and the network is run with 1 ms timestep. Connectivity probability is set at 1%, additional analysis (including 0.1%, 5% and 10% connectivities) can be found in the Supplementary Material. The same type of experiment was run for the plastic case, with the exception of the connections being defined through STDP with Spike-Pair rule for timing dependence and additive weight dependence (Morrison et al., 2008). The number of firing neurons has been reduced compared to the static case, as synaptic processing for plastic synapses requires additional steps (as highlighted in Section 3.5). For details regarding population sizes and the employed plasticity rule, please refer to the Supplementary Material.

**Figure 13:**
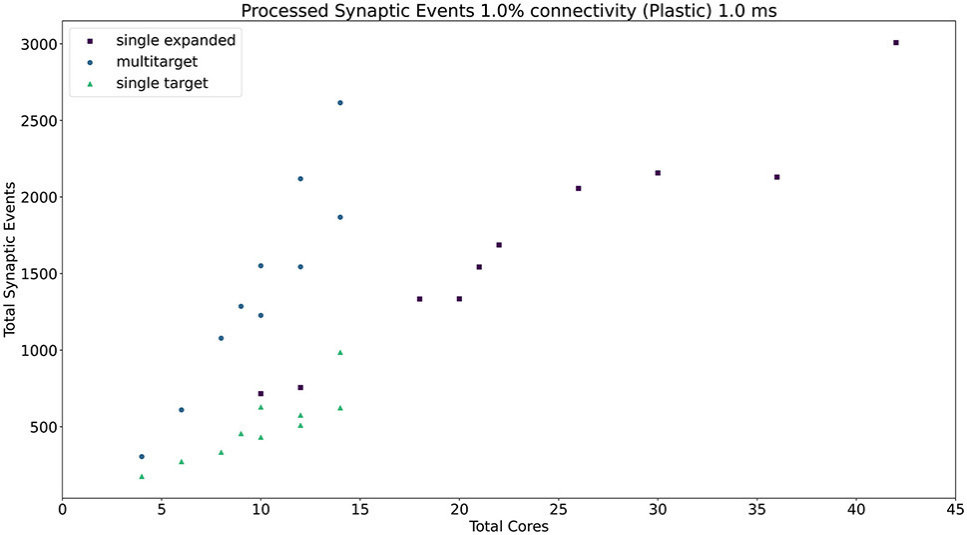
Resource allocation vs. peak performance for the three different approaches (*single target expanded, multi-target*, and *single target*). The network is the same used for Figure 12, with 1% connectivity and plastic connections. The color scheme matches that used in Figure 12. For a case by case labeled version of this plot, please refer to the Supplementary Material.

Similarly to the static case, the *Multi-target* approach shows better performance than the *single target* case for all simulated configurations, demonstrating again that the approach makes better use of the available resources. For very sparse networks, with plastic synapses, the *Multi-target* approach achieves peak synaptic event throughput very close to the *single target expanded* simulations. This is due to the differences in processing plastic synapses compared to static synapses. Plasticity, requires the updated weights to be written back to shared memory, therefore doubling the accesses to SDRAM compared to the static case. This operation becomes extremely costly when the number of receptors per row are limited. Therefore, having longer synaptic rows, as in the case of the *Multi-target* approach, allows to further increase the number of synaptic events that can be processed per timestep. Figure 13 contains a comparison of the general trend for the three approaches (refer to the Supplementary Material for a case by case labeled representation of this plot). Similarly to the static case, the Multi-target partitioning shows the steepest increase of processed synaptic events per timestep (vertical axis) with increasing allocated resources (horizontal axis). This further demonstrates that the Multi-target partitioning achieves better performance than previous approaches when the same hardware resources are available and comparable results with reduced hardware requirements, also for SNN simulations involving synaptic plasticity.

### 4.3. Sparsity Profiling

#### 4.3.1. Experiment Description

Profiling of peak synaptic event throughput with a range of connection sparsity levels is now explored. This experiment shows the variation of the processed synaptic events per timestep with increasing numbers of target *Neuron* cores. The number of *Synapse* cores is kept fixed and the target *Neuron* cores are gradually increased. In order to provide a good balance (and according to the peak performance shown in Section 4.2), the chosen number of *Synapse* cores is 7 and the target *Neuron* cores range from 1 to 7, guaranteeing to fit on a single chip. This allocation also allows equal comparison between simulations with 1 ms timestep resolution and 0.1 ms, having set the number of neurons per *Neuron* core in both cases to 64. The connectivity probabilities investigated are: 0.1, 1, 10, and 50%. Connectivity patterns above 50% are beyond the scope of this study, as they are extremely rare in biology (Hagmann et al., 2008), and are handled sufficiently well by traditional hardware (GPUs, CPUs, etc.). The network employed for this experiment has a structure analogous to that described in Section 4.2.1. For this case various sparsity patterns are shown, together with different cores allocations per chip. This experiment is useful to demonstrate the flexibility of the approach in handling multiple sparsity levels, a common feature in biologically-representative SNNs (Schmidt et al., 2018).

#### 4.3.2. Sparsity Results

**Figure 14:**
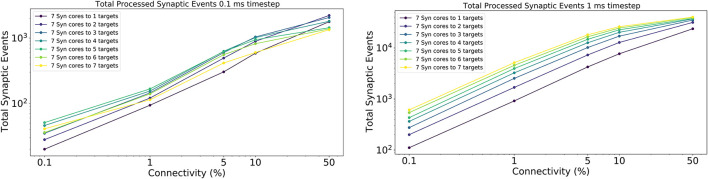
Processed synaptic events for different connectivity configurations. Both the 0.1 ms timestep **(left)** and 1 ms timestep **(right)** cases are shown. The vertical axis shows the total processed synaptic events per timestep, the horizontal axis different connectivity probabilities. Each line represents a different neural ensemble with constant *Synapse* cores, but increasing target *Neuron* cores.

The results for this experiment are shown in Figure 14 left for 0.1 ms timestep resolution and in Figure 14 right for 1 ms timestep resolution. The horizontal axis shows the connectivity probabilities, the vertical axis the processed synaptic events per timestep. Each line represents a different configuration of *Synapse* cores to *Neuron* cores, where each *Synapse* core is connected to all the targets of that configuration. The number of postsynaptic receptors per *Synapse* core therefore can be obtained by multiplying the number of *Neuron* cores by 64 (number of neurons per *Neuron* core).

For the 1 ms case (Figure 14 right), as expected, simulations with higher number of targets process the highest number of synaptic events per timestep. The most evident jump happens between the configurations with 1 and 2 targets, respectively, where the synaptic rows double in size. This shows that having larger synaptic rows impacts processing times, especially for very sparse networks, by improving the processed synaptic events of ≈ 1 order of magnitude for 0.1% connectivity between worst and best case. This gain reduces when the connectivity probability increases, because of multiple synaptic events are carried per spike. Therefore, the time processing per spike increases as well.

The 0.1 ms case (Figure 14 left) follows a similar trend to the 1 ms case, however the examples with 6 and 7 targets do not give any improvements. The reason for this is due to the time required to perform the transfers between shared and local memories for the synaptic contributions, which have a higher impact on the timestep relative to the 1 ms case. For the sparse simulations (0.1% and 1% connectivity), having multiple target *Neuron* cores gives advantage similarly to the 1 ms case, however, when the network becomes denser the trend starts to invert, as the cost of processing a single incoming spike dominates over the gain introduced by this approach.

## 5. Discussion

This work presents a novel parallelization approach for neural processing on Neuromorphic hardware, which improves the performance of SNN simulations by acting on the way synaptic matrices are partitioned and processed. The Multi-target partitioning approach provides additional freedom when designing SNN simulations, by allowing to target applications more specifically, according to their requirements. By allowing parameterization of synaptic and neural processing units, it is possible to allocate the appropriate amount of resources for a given requirement, prioritizing the number of *Neuron* processing units for sparser applications and increasing the number of *Synapse* processing units when the fan-in dominates. Thanks to these improvements it is possible to maximize the performance, while using minimal hardware resources and therefore reducing power consumption.

Through a SpiNNaker implementation of the Multi-target partitioning approach, it is possible to improve the peak synaptic processing throughput up to 9× compared to previous results for the same hardware resources. Furthermore, it is possible to obtain comparable processed synaptic events per ms, by reducing the hardware resources to a quarter, resulting in a much smaller machine (and energy consumption) dedicated to the simulation (as detailed in Section 4.2).

The Multi-target partitioning approach additionally enables optimal processing of incoming spike packets, providing a larger pool of target neurons for each spike, hence increasing the length of processed synaptic rows for a given connection density. This greatly reduces the required number of accesses to shared memory per timestep, therefore allowing more efficient processing of sparsely connected networks (detailed in Section 4.3). This is shown by Equations (8) and (13), where the number of target neurons of each spike grows according to the number of target *Neuron* cores, expanding the limit beyond a single postsynaptic *Neuron* core. This has the effect of reducing, by a factor *N*_*c*_ the number of destination processors per spike packet, facilitating the routing of spike packets and so reducing the pressure on the communication fabric. Furthermore, this increased number of targets per spike packet allows to amortize the dominating fixed cost of processing a spike (*c*_*s*_) (Rhodes et al., 2018) over a higher number of postsynaptic receptors, which can now be larger than that of a single *Neuron* core, overcoming this limitation which is still observed for the Heterogeneous partitioning.

The Multi-target partitioning approach is optimal as it comes with minimal additional costs compared to previous approaches. However, the SpiNNaker implementation is limited by the different access patterns to shared memory. The shared memory access time plays a key role in the fraction of the timestep available for spike processing, as shown by Equation (9) and by the recorded values presented in Sections 4.1.2 and 4.1.3. The relatively old technology employed by SpiNNaker represents a bottleneck in this context, resulting in both memory contention and transfer size limiting the total system throughput. This causes the synaptic contributions writing (*t*_*w*_) and reading (*t*_*r*_) times (Equations 9–11) to increase with the number of cores in the ensemble, consuming approximately half the timestep duration for high timestep resolution simulations such as 0.1 ms. For this reason the need for faster access to shared memory is proven, by showing that there is a large potential gain in having access to multiple separate shared memories, compared to a single shared memory. This consideration opens up to the possibility of using more advanced memory architectures for Neuromorphic hardware, such as multiport memories, since structures like synaptic matrices and synaptic contributions are non-overlapping and therefore would benefit from the capability of separate independent accesses.

The flexibility of the approach also makes it portable and extendable for the next generation of digital Neuromorphic platforms. SpiNNaker 2, by exploiting its chip organization of cores in quartets, namely QPEs (Höppner et al., 2021; Yan et al., 2021), could map a cluster-based implementation of multiple neural ensembles per chip, where each processor (PE) represents either a *Neuron* core or a *Synapse* core. Since each PE has the capability to efficiently access the local memory of other PEs on the same QPE, it is possible to efficiently share the synaptic contributions within a QPE, overcoming the contention issue. A step further would include a tree-like structure, where QPEs could implement a group of 4 *Synapse* cores, which generate the synaptic contributions as a single block for the 4 cores. Then, a single PE per QPE accesses the chip shared memory to communicate with other QPEs implementing blocks of *Neuron* cores. Following the same strategy, a single *Neuron* core per *Neuron* QPE accesses the shared memory to retrieve the contributions. This would expand the ensemble capabilities to a full chip (up to 160 cores), limiting the memory contention to a quarter of the cores in use, which combined with the much higher memory throughput (6 vs. 1 GB/s for the SpiNNaker SDRAM) would have a large impact on the synaptic contributions reading and writing times.

The Multi-target partitioning approach also has potential benefits in Neuromorphic systems where all synaptic information is stored locally to the computational units. For these systems the approach would allow synaptic compartments to target multiple neural compartments, improving the handling of sparse connections, and overcoming the limitations set by the fixed coupling between synaptic and neural units. Furthermore the added benefits seen when processing plastic connections offers advantages for online learning applications, particularly in sparsely-connected biologically-representative SNNs.

## Data Availability Statement

The material and the code generated for this study, as well as the experiments, are available from the SpiNNaker software stack: https://github.com/SpiNNakerManchester, using the branch Multitarget_syn_cores.

## Author Contributions

LP led the design of the Multi-target partitioning model, built the SpiNNaker implementation, designed and ran the experiments and drafted the manuscript. OR co-designed the Multi-target partitioning model and supervised the research. Both authors read, commented, and approved the final manuscript.

## Funding

The design and construction of the SpiNNaker machine was supported by EPSRC (the UK Engineering and Physical Sciences Research Council) under grant EP/D07908X/1 and EP/G015740/1, in collaboration with the universities of Southampton, Cambridge, and Sheffield and with industry partners ARM Ltd., Silistix Ltd., and Thales. Ongoing development of the software is supported by the EU ICT Flagship Human Brain Project (H2020 785907 and 945539), in collaboration with many university and industry partners across the EU and beyond. LP was funded by an EPSRC DTA studentship in the Department of Computer Science.

## Conflict of Interest

The authors declare that the research was conducted in the absence of any commercial or financial relationships that could be construed as a potential conflict of interest.

## Publisher's Note

All claims expressed in this article are solely those of the authors and do not necessarily represent those of their affiliated organizations, or those of the publisher, the editors and the reviewers. Any product that may be evaluated in this article, or claim that may be made by its manufacturer, is not guaranteed or endorsed by the publisher.

## References

[B1] Akopyan F. Sawada J. Cassidy A. Alvarez-Icaza R. Arthur J. Merolla P.. (2015). TrueNorth: design and tool flow of a 65 mW 1 million neuron programmable neurosynaptic chip. IEEE Trans. Comput. Aided Design Integr. Circ. Syst. 34, 1537–1557. 10.1109/TCAD.2015.2474396

[B2] ARM (2006). ARM968E-S Technical Reference Manual. ARM. Available online at: https://developer.arm.com/documentation/ddi0311/

[B3] Bogdan P. A. Marcinne B. Casellato C. Casali S. Rowley A. G. Hopkins M.. (2021). Towards a bio-inspired real-time neuromorphic cerebellum. Front. Cell. Neurosci. 15, 622870. 10.3389/fncel.2021.622870 34135732 PMC8202688

[B4] Casali S. Marenzi E. Medini C. Casellato C. D'Angelo E. (2019). Reconstruction and simulation of a scaffold model of the cerebellar network. Front. Neuroinform. 13, 37. 10.3389/fninf.2019.00037 31156416 PMC6530631

[B5] Davies M. Srinivasa N. Lin T.-H. Chinya G. Cao Y. Choday S. H.. (2018). Loihi: a neuromorphic manycore processor with on-chip learning. IEEE Micro 38, 82–99. 10.1109/MM.2018.112130359

[B6] Davison A. Bruderle D. Eppler J. Kremkow J. Muller E. Pecevski D.. (2009). PyNN: a common interface for neuronal network simulators. Front. Neuroinform. 2, 11. 10.3389/neuro.11.011.2008 19194529 PMC2634533

[B7] Furber S. B. Galluppi F. Temple S. Plana L. A. (2014). The SpiNNaker project. Proc. IEEE 102, 652–665. 10.1109/JPROC.2014.2304638

[B8] Furber S. B. Lester D. R. Plana L. A. Garside J. D. Painkras E. Temple S.. (2013). Overview of the SpiNNaker system architecture. IEEE Trans. Comput. 62, 2454–2467. 10.1109/TC.2012.142

[B9] Galluppi F. Lagorce X. Stromatias E. Pfeiffer M. Plana L. A. Furber S. B.. (2015). A framework for plasticity implementation on the SpiNNaker neural architecture. Front. Neurosci. 8, 429. 10.3389/fnins.2014.00429 25653580 PMC4299433

[B10] Gerstner W. Kistler W. M. (2002). Spiking Neuron Models: Single Neurons, Populations, Plasticity. (Cambridge: Cambridge University Press). 10.1017/CBO9780511815706

[B11] Gewaltig M.-O. Diesmann M. (2007). NEST (NEural Simulation Tool). Scholarpedia 2, 1430. 10.4249/scholarpedia.1430

[B12] Hagmann P. Cammoun L. Gigandet X. Meuli R. Honey C. J. Wedeen V. J.. (2008). Mapping the structural core of human cerebral cortex. PLoS Biol. 6, e159. 10.1371/journal.pbio.0060159 18597554 PMC2443193

[B13] Heittmann A. Psychou G. Trensch G. Cox C. E. Wilcke W. W. Diesmann M.. (2022). Simulating the cortical microcircuit significantly faster than real time on the IBM INC-3000 neural supercomputer. Front. Neurosci. 15, 728460. 10.3389/fnins.2021.728460 35126034 PMC8811464

[B14] Höppner S. Yan Y. Dixius A. Scholze S. Partzsch J. Stolba M.. (2021). The SpiNNaker 2 processing element architecture for hybrid digital neuromorphic computing. arXiv preprint arXiv:2103.08392. 10.48550/arXiv.2103.08392

[B15] Indiveri G. Linares-Barranco B. Hamilton T. van Schaik A. Etienne-Cummings R. Delbruck T.. (2011). Neuromorphic silicon neuron circuits. Front. Neurosci. 5, 73. 10.3389/fnins.2011.00073 21747754 PMC3130465

[B16] Ippen T. Eppler J. M. Plesser H. E. Diesmann M. (2017). Constructing neuronal network models in massively parallel environments. Front. Neuroinform. 11, 30. 10.3389/fninf.2017.00030 28559808 PMC5432669

[B17] Knight J. C. Furber S. B. (2016). Synapse-centric mapping of cortical models to the SpiNNaker neuromorphic architecture. Front. Neurosci. 10, 420. 10.3389/fnins.2016.00420 27683540 PMC5022244

[B18] Knight J. C. Komissarov A. Nowotny T. (2021). PyGeNN: a Python library for GPU-enhanced neural networks. Front. Neuroinform. 15, 659005. 10.3389/fninf.2021.659005 33967731 PMC8100330

[B19] Knight J. C. Nowotny T. (2021). Larger GPU-accelerated brain simulations with procedural connectivity. Nat. Comput. Sci. 1, 136–142. 10.1038/s43588-020-00022-7 38217218

[B20] Kurth A. C. Senk J. Terhorst D. Finnerty J. Diesmann M. (2021). Sub-realtime simulation of a neuronal network of natural density. Neuromorph. Comput. Eng. 2, 021001. 10.1088/2634-4386/ac55fc

[B21] Levy W. B. Calvert V. G. (2020). Computation in the human cerebral cortex uses less than 0.2 watts yet this great expense is optimal when considering communication costs. bioRxiv. 1, 1–13. 10.1101/2020.04.23.057927

[B22] Mavaridas J. Lujan M. Plana L. A. Temple S. Furber S. B. (2015). SpiNNaker: enhanced multicast routing. Parallel Comput. 45, 49–66. 10.1016/j.parco.2015.01.002

[B23] Mead C. (1989). Analog VLSI and Neural Systems. (Boston, MA: Addison-Wesley Longman Publishing Co., Inc).

[B24] Mead C. (1990). Neuromorphic electronic systems. Proc. IEEE 78, 1629–1636. 10.1109/5.58356

[B25] Moradi S. Qiao N. Stefanini F. Indiveri G. (2018). A scalable multicore architecture with heterogeneous memory structures for dynamic neuromorphic asynchronous processors (DYNAPs). IEEE Trans. Biomed. Circ. Syst. 12, 106–122. 10.1109/TBCAS.2017.2759700 29377800

[B26] Morrison A. Diesmann M. Gerstner W. (2008). Phenomenological models of synaptic plasticity based on spike timing. Biol. Cybern. 98, 459–478. 10.1007/s00422-008-0233-1 18491160 PMC2799003

[B27] Morrison A. Mehring C. Geisel T. Aertsen A. Diesmann M. (2005). Advancing the boundaries of high-connectivity network simulation with distributed computing. Neural Comput. 17, 1776–1801. 10.1162/0899766054026648 15969917

[B28] Painkras E. Plana L. A. Garside J. Temple S. Galluppi F. Patterson C.. (2013). SpiNNaker: a 1-W 18-core system-on-chip for massively-parallel neural network simulation. IEEE J. Solid State Circ. 48, 1943–1953. 10.1109/JSSC.2013.2259038

[B29] Plana L. A. Clark D. Davidson S. Furber S. B. Garside J. D. Painkras E.. (2011). SpiNNaker: design and implementation of a GALS multicore system-on-chip. ACM J. Emerg. Technol. Comput. Syst. 4, 1–18. 10.1145/2043643.2043647

[B30] Potjans T. C. Diesmann M. (2012). The cell-type specific cortical microcircuit: relating structure and activity in a full-scale spiking network model. Cereb. Cortex 24, 785–806. 10.1093/cercor/bhs358 23203991 PMC3920768

[B31] Rhodes O. Bogdan P. A. Brenninkmeijer C. Davidson S. Fellows D. Gait A.. (2018). sPyNNaker: a software package for running PyNN simulations on SpiNNaker. Front. Neurosci. 12, 816. 10.3389/fnins.2018.00816 30524220 PMC6257411

[B32] Rhodes O. Peres L. Rowley A. G. D. Gait A. Plana L. A. Brenninkmeijer C.. (2019). Real-time cortical simulation on neuromorphic hardware. Philos. Trans. R. Soc. A Math. Phys. Eng. Sci. 378. 10.1098/rsta.2019.0160 31865885 PMC6939236

[B33] Rotter S. Diesmann M. (1999). Exact digital simulation of time-invariant linear systems with applications to neuronal modeling. Biol. Cybern. 81, 381–402. 10.1007/s004220050570 10592015

[B34] Rowley A. G. D. Brenninkmeijer C. Davidson S. Fellows D. Gait A. Lester D. R.. (2019). SpiNNTools: the execution engine for the SpiNNaker platform. Front. Neurosci. 13, 231. 10.3389/fnins.2019.00231 30971873 PMC6444189

[B35] Schemmel J. Kriener L. Muller P. Meier K. (2017). An accelerated analog neuromorphic hardware system emulating NMDA- and calcium-based non-linear dendrites, in 2017 International Joint Conference on Neural Networks (IJCNN). (Anchorage), 2217–2226. 10.1109/IJCNN.2017.7966124

[B36] Schmidt M. Bakker R. Shen K. Bezgin G. Diesmann M. van Albada S. J. (2018). A multi-scale layer-resolved spiking network model of resting-state dynamics in macaque visual cortical areas. PLoS Comput. Biol. 14, e1006359 10.1371/journal.pcbi.1006359 30335761 PMC6193609

[B37] Sharp T. Furber S. B. (2013). Correctness and performance of the SpiNNaker architecture, in The 2013 International Joint Conference on Neural Networks (IJCNN). (Dallas), 1–8. 10.1109/IJCNN.2013.6706988

[B38] Sharp T. Plana L. A. Galluppi F. Furber S. B. (2011). Event-driven simulation of arbitrary spiking neural networks on SpiNNaker, in ICONIP. (Heidelberg) 10.1007/978-3-642-24965-5_48

[B39] van Albada S. J. Rowley A. G. Senk J. Hopkins M. Schmidt M. Stokes A. B.. (2018). Performance comparison of the digital neuromorphic hardware SpiNNaker and the neural network simulation software NEST for a full-scale cortical microcircuit model. Front. Neurosci. 12, 291. 10.3389/fnins.2018.00291 29875620 PMC5974216

[B40] Yan Y. Stewart T. C. Choo X. Vogginger B. Partzsch J. Hoppner S.. (2021). Comparing Loihi with a SpiNNaker 2 prototype on low-latency keyword spotting and adaptive robotic control. Neuromorph. Comput. Eng. 1, 014002. 10.1088/2634-4386/abf150

