# Supplementary material for: Parallelization of Neural Processing on Neuromorphic Hardware
Source: Front Neurosci. 2022 May 10;16:867027. doi: 10.3389/fnins.2022.867027 (PMC9128596; doi:10.3389/fnins.2022.867027)
Supplement: Supplementary file 1 [file Data_Sheet_1.pdf]

Supplementary Material: Parallelization of Neural Processing on Neuromorphic Hardware

1 MEMORY IMPACT

The analysis presented in Section 4.1 focused on the worst case reading and writing times for the synaptic contributions using the Multi-target partitioning approach. This type of analysis is useful to define the general system requirements, however it does not provide information on the general behaviour of the remaining cores on the machine. For this reason here we provide additional results, showing the recorded minimum and average reading and writing times, obtained from the same test. These values provide a more detailed view on memory read and write performance, showing where optimisations are possible. This section is structured similarly to the main material, separating the reading cases from the writing cases.

1.1 Reading Times

The results presented here are in the same format as those shown in the main material: heatmaps with increasing *Synapse* cores on the horizontal axis (representing increasing DMA reads per timestep) and *Neuron* cores on the vertical axis (indicating increasing memory access contention) are presented. Each Figure contains two heatmaps having the dual memory case (left) and the SDRAM only memory case (right).

Figure 1 contains the best case reading times. Comparing the two cases, the dual memory allocation always provides better results than the SDRAM only case, especially when the contention increases (from top to bottom) and the number of transfers per core becomes higher (from left to right). It is important to notice the difference between best case and worst case (main material), to understand how much the placement of cores impacts on the measured times. Best case reading times show improvements of up to 5 μs, representing a considerable impact on 0.1 ms timesteps simulations (5% of the overall simulation timestep).

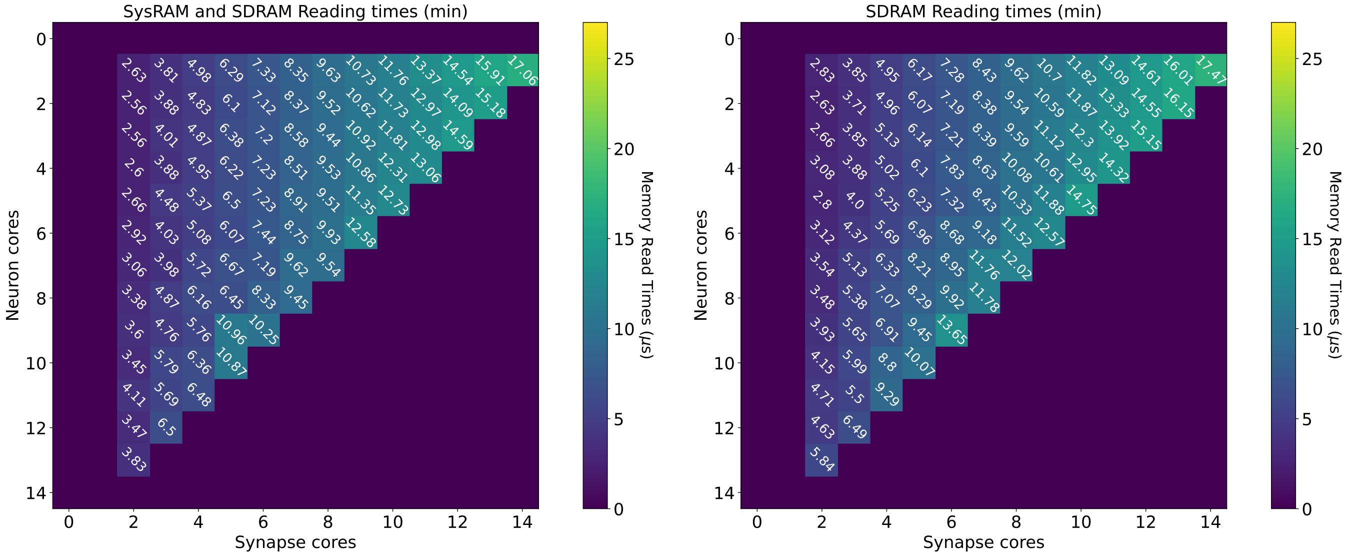

Figure 1. Best Case DMA reading Timings for increasing synaptic and *Neuron* cores

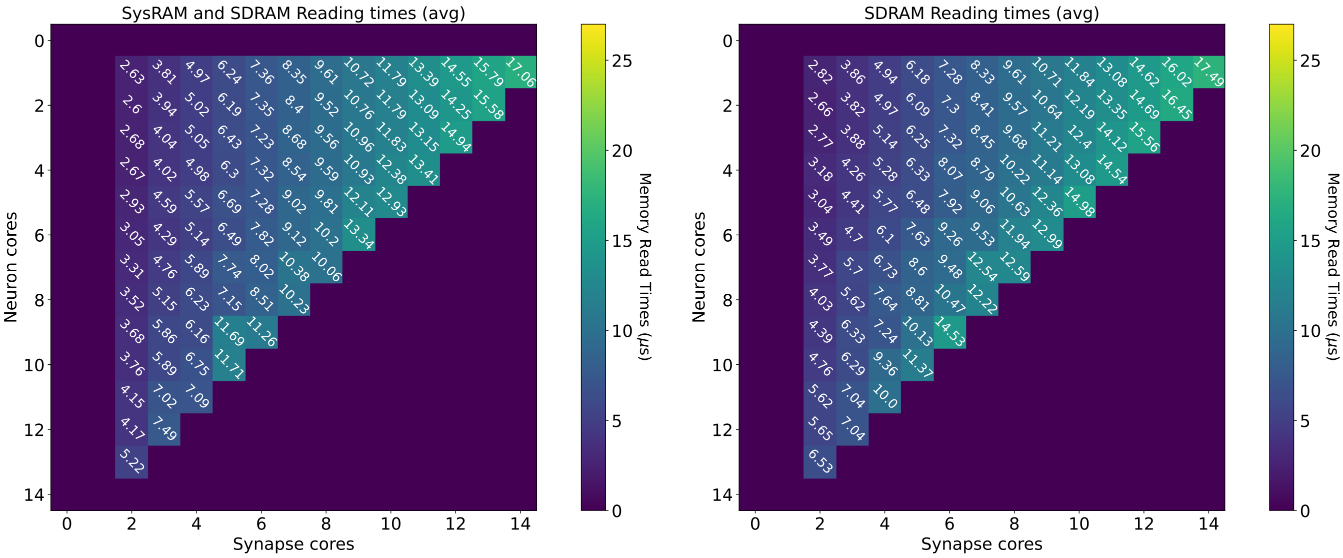

Figure 2. Average case DMA reading Timings for increasing synaptic and *Neuron* cores

Similar considerations are recorded for the average case, shown in Figure 2. The average values however are closer to the minimum, for most of the cases, which shows that the majority of the cores act similarly to the best case, and that the worst case tends to be isolated.

The core allocation phase also plays a key role in the memory timings, due to the architectural features presented in the main material. This is confirmed by the presented results, and suggests that a more targeted placement can achieve lower access times. This can be performed by informing the SpiNNaker toolchain of the type of cores (*Synapse* and *Neuron* cores), so that the most critical processors, according to the application requirement, are placed on less contended branches of the memories access trees. This is however a non-trivial operation, as, due to fault tolerance, it is hard to predict which core is available on each chip, and whether all the cores on the chip will be functioning.

1.2 Writing Times

Write times are also profiled, and presented here following the format above. Heatmaps with increasing *Synapse* cores on the horizontal axis (representing increasing access contention) and *Neuron* cores on the vertical axis (indicating increasing transfer size) are shown. The dual memory case is the left heatmap, while SDRAM only is presented on the right.

Figure 3 shows the best case writing times. For the writing phase it is more evident how the placement of cores impacts on the transfer time (relative to the reading phase above). Comparing the best with the worst case (main material), significant gains are seen, particularly for the dual memory case. This difference becomes larger when the contending cores increase, and in some cases amounts to 15 μs, representing a significant impact on 0.1 ms timestep simulations (15% of the overall timestep). A similar comparison is performed between the dual-memory and SDRAM-only best cases, showing that with higher contention the writing time is halved with the dual-memory approach, proving that it is possible to half the contention by using separate memories with separate accesses.

Similar considerations are made regarding the average case results (shown in Figure 4), where access times are up to 6 μs smaller than worst case for dual memories. The SDRAM-only case, however, shows numbers closer to worst case, and appears to perform better than the dual memory case with very low contention (2 *Synapse* cores) on the average case analysis. This can be explained by the fact that SysRAM

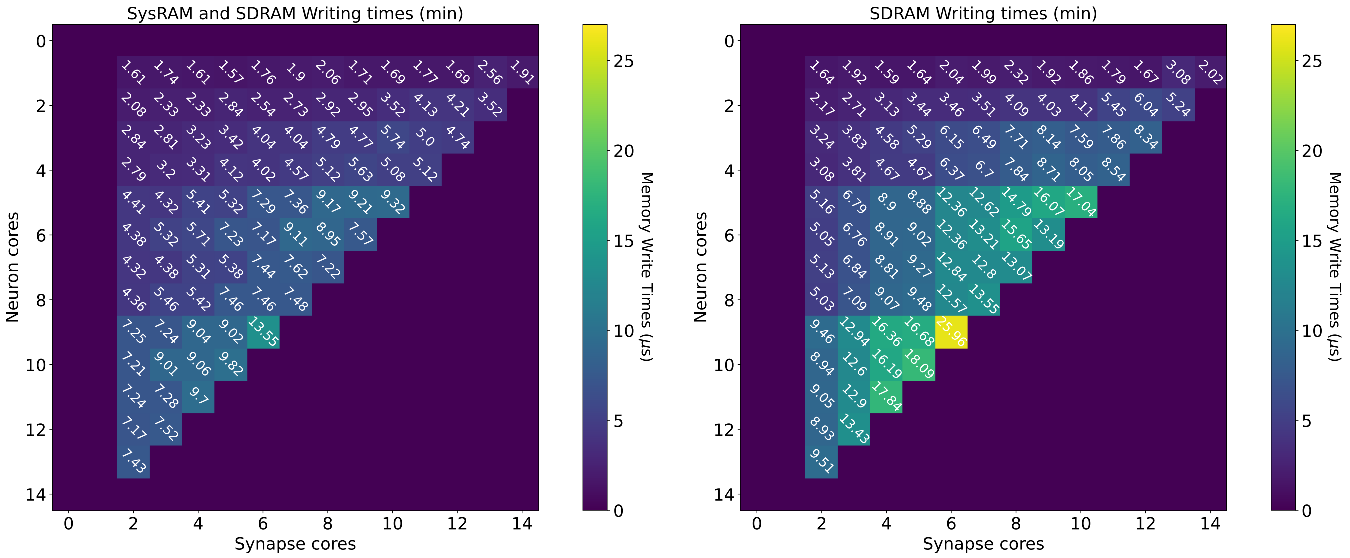

Figure 3. Best case DMA writing Timings for increasing synaptic and *Neuron* cores

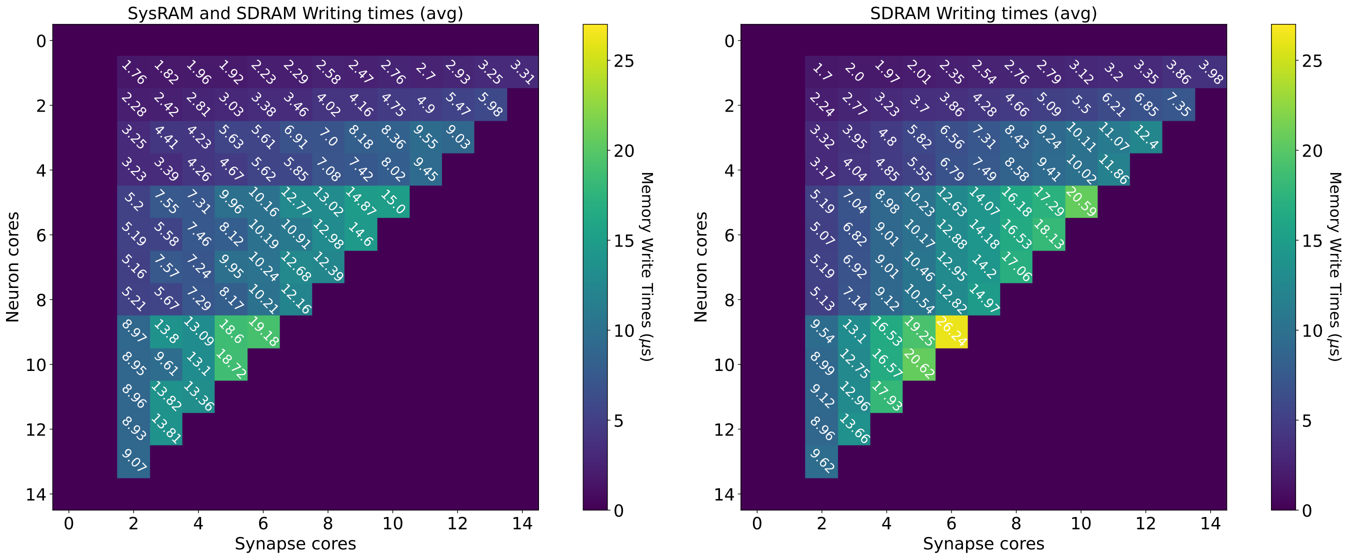

Figure 4. Average case DMA writing Timings for increasing synaptic and *Neuron* cores

has a slower transfer rate, therefore, when contention is low this has a higher impact on the total time. However, when contention increases, the dual-memory case usage drastically reduces the access time (as only half of the cores are contending the access), therefore reducing the total transfer time, relative to the SDRAM-only case.

These results again show the impact of a sub-optimal placement of cores on a chip, and demonstrate that an improved placement strategy, combined with independent memory accesses can have a significant impact on the fraction of the timestep available for neural processing, especially for critical cases such as 0.1 ms timestep simulations.

2 PEAK THROUGHPUT PERFORMANCE

The experiment presented in Section 4.2 of the main paper shows comparisons between the Heterogeneous model and the Multi-target partitioning, regarding the processed synaptic events per timestep. The analysis

Presynaptic Population Size			
	1 ms Timestep(static)	0.1 ms Timestep	1 ms Timestep (plastic)
2,2	476	40	476
3,3	833	63	715
4,2	1000	77	741
4,4	1000	77	770
5,5	1428	100	770
6,3	1667	111	833
6,6	1667	111	909
7,7	2000	125	1000
8,2	2000	143	1428
8,4	2000	143	1428
10,2	2500	N/A	1667
12,2	2500	N/A	2000

Table 1. Presynaptic firing neurons for the peak processing benchmark according to the timestep resolution (columns) and allocated postsynaptic resources (rows).

included a comparison using the same amount of hardware resources between the Heterogeneous model and the Multi-target partitioning, and a third case where the Heterogeneous model was configured using the same input structure per ensemble of the Multi-target partitioning, but requiring a considerably more hardware resources (the case termed *single expanded*). These two comparisons allowed to measure both how the Multi-target partitioning improves performance for a given allocation of hardware resources, and the relative resource reduction enabled to preserve a given performance level.

A test network composed of two separate populations was used as a benchmark and performance was profiled for static and plastic connections. Static profiling is reported for both 1 ms and 0.1 ms timestep resolutions; plasticity on the other hand required 1 ms timesteps, because of the additional operations related to weight updates. Here we provide additional details on the network topology, together with additional test cases containing a range of connection densities, including sparser connectivity patterns at $P_c = 0.1\%$, and more common levels of 5% and 10%, in order to show the scaling of the of the approach.

2.1 Network Details

Table 1 contains the presynaptic population sizes for all test cases. Each row represents a core allocation case (as reported in plots in the main paper), and each column a different timestep resolution.

The values are obtained from a population of 10000 neurons. Multiple simulations have been performed gradually decreasing the presynaptic sizes, while monitoring the state of the SpiNNaker communication network. These values represent the biggest sizes for which all the spike packets were delivered to their destinations without adding network delays or creating network congestion, and at the same time they are enough to generate more synaptic events than the processing capabilities of the allocated *Synapse* cores for each case. This allowed to test the limit of the processing capabilities of the various approaches.

For the 0.1 ms timestep resolution, the last two tests have been omitted. For these cases the timestep resolution represents a limiting factor on the number of *Synapse* cores per ensemble, as the *Neuron* cores cannot retrieve the synaptic contributions for more than 8 *Synapse* cores and update the neural state, while honoring the real-time performance. Therefore all configurations with more than 8 *Synapse* cores per ensemble cannot be simulated with 0.1 ms timesteps on SpiNNaker.

2.1.1 Plasticity Rule

For the plastic experiment connections were implemented through STDP using Spike-pair rule and additive weight dependence. This rule has been chosen as it captures well the main features of STDP rules. The mathematical formulation of the rule is expressed by Equation 1 (Morrison et al., 2008).

$$\begin{aligned} \Delta w^{+} &= A_{+}exp(-|\Delta t|/\tau_{+}) \qquad if \;\; \Delta t > 0 \\ \Delta w^{-} &= -A_{-}exp(-|\Delta t|/\tau_{-}) \qquad if \;\; \Delta t \leq 0 \end{aligned} \tag{1}$$

The term $\Delta t = t_{post} - t_{pre}$ represents the temporal difference between post- and presynaptic spikes. The time constants for potentiation and depression are indicated by τ_{+} and τ_{-} respectively. The rate of learning is expressed by A_{+} and A_{-}. The rule requires to maintain a low-passed filtered version of the presynaptic spike train, and one for the postsynaptic spike train. These are expressed by the two exponentials. The presynaptic trace on SpiNNaker is stored in the synaptic matrices for each row. This is made available to the *Synapse* cores upon retrieval of a synaptic row when a spike is received. The postsynaptic trace is maintained locally on the *Synapse* cores in the form of array, where each slot corresponds to a postsynaptic neuron. In order to correctly update the postsynaptic trace, *Synapse* cores needs to retrieve information on postsynaptic spiking from the *Neuron* cores, as described in Section 3.5 from the main paper. Therefore, according to Equation 1, the presynaptic trace is used to evaluate depression and the postsynaptic trace for potentiation. The final weight update is calculated by summing together the two contributions.

2.2 Static Profiling

Here we present the results of the test run for the three different allocations and the baseline set by the homogeneous partitioning, using different sparsity levels and static connections. Bar charts presenting the peak processed Synaptic events per timestep are shown in Figure 5-10. Each configuration contains four bars (purple for the Heterogeneous *single target expanded*, blue for the *Multi-target*, green for the Heterogeneous model with same resources as the Multi-target approach and yellow for the baseline case). Dual horizontal axes are utilised: showing on the bottom the core allocations for the green and blue bars; and on the top the allocation for the purple bars. The two numbers represent *Synapse* cores and *Neuron* cores respectively. In the case of the Multi-target partitioning, all *Synapse* cores are connected with all *Neuron* cores; for the Heterogeneous model the target is single, and the number of *Synapse* cores per *Neuron* core is obtained by dividing the first number by the second.

Figures 5 and 6 show the results with 0.1% connectivity with 0.1 ms and 1 ms stimestep resolutions respectively, Figures 7 and 8 are for 5% connectivity, and Figures 9 and 10 for 10%.

The measured values here reflect the general behaviour shown in the main paper with 1% connectivity. The Multi-target partitioning performs better than the single Heterogeneous approach in all the presented cases, and represents the optimal choice for a given allocation of hardware resources. This is again more evident in the [7, 7] case, here performing more than 9$\times$ better. Results are also comparable with the *single expanded* case for the 1 ms simulations in most cases, which makes the Multi-target approach the best solution, only requiring a fraction of the hardware resources. Another interesting result is the fact that the improvement given by the Multi-target approach compared to the Heterogeneous cases becomes more pronounced with higher connection probabilities, demonstrating that this approach also scales well with SNN connection density.

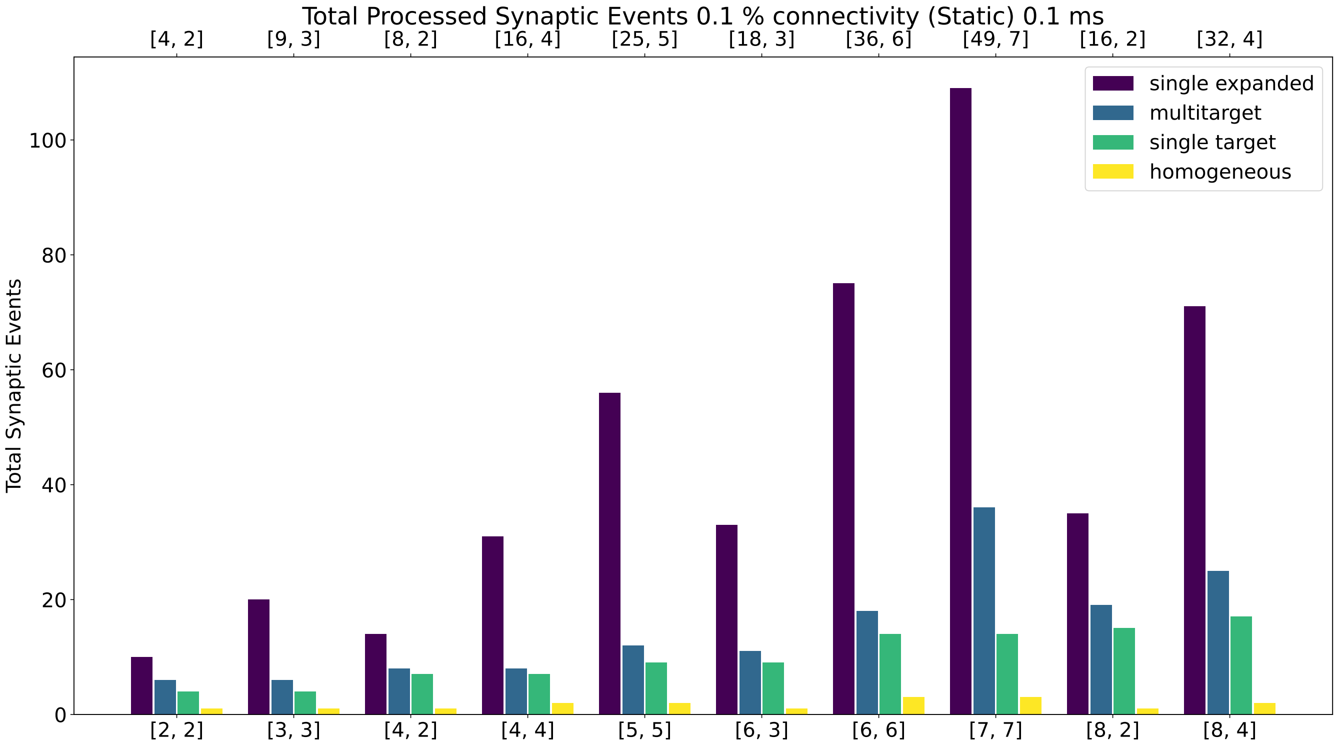

Figure 5. Processed synaptic events per timestep, using 0.1 ms timestep resolution using 64 neurons per core. Connectivity probability set to 0.1%.

Regarding the 0.1 ms results, the general trend follows the 1 ms results, showing that the Multi-target approach is still optimal. However there are some edge cases where the gain is small, particularly with 0.1% connectivity. The reason behind this is due to the extreme sparsity and the resulting empty packets representing spikes which target no neurons on the destination computational unit. The SNN is relatively small in this case, and the total number of synaptic events that are effectively received is small. However the [7, 7] case in Figure 5, demonstrates again that having longer synaptic rows (the Multi-target approach employs synaptic rows 7 times longer than the Heterogeneous model) allows to process many more synaptic events per timestep ($\approx 3\times$ here). The reason behind large discrepancies between the extended Heterogeneous approach and the Multi-target with 0.1 ms timesteps, as in the case of the [7, 7] is due to the much higher computational power of the extended approach compared to the Multi-target. With 0.1 ms timesteps the reading and writing times have a much greater impact on the time available for synaptic processing, therefore in this case there are where there are $7\times$ more *Synapse* cores, this translates into improved peak performance.

To prove the robustness of the approach, the same experiment was run with 1 ms timestep resolution employing 256 neurons per core. The results are presented in form of barcharts in Figures 11, 12, 13 and 14. The results show very similar behaviour compared to the cases employing 64 neurons per core, showing that the Multi-target partitioning improves the processed synaptic events per timestep compared to the single target Heterogeneous partitioning. The optimality of the Multi-target partitioning is proven against the single target expanded case as well, showing that it is possible to reach comparable performance with a fraction of the employed resources. Furthermore, the number of processed synaptic events per core using the Multi-target partitioning is higher than the number of processed synaptic events per core using the single target expanded, for all the considered cases. This shows a higher throughput per core for the Multi-target partitioning than any other method.

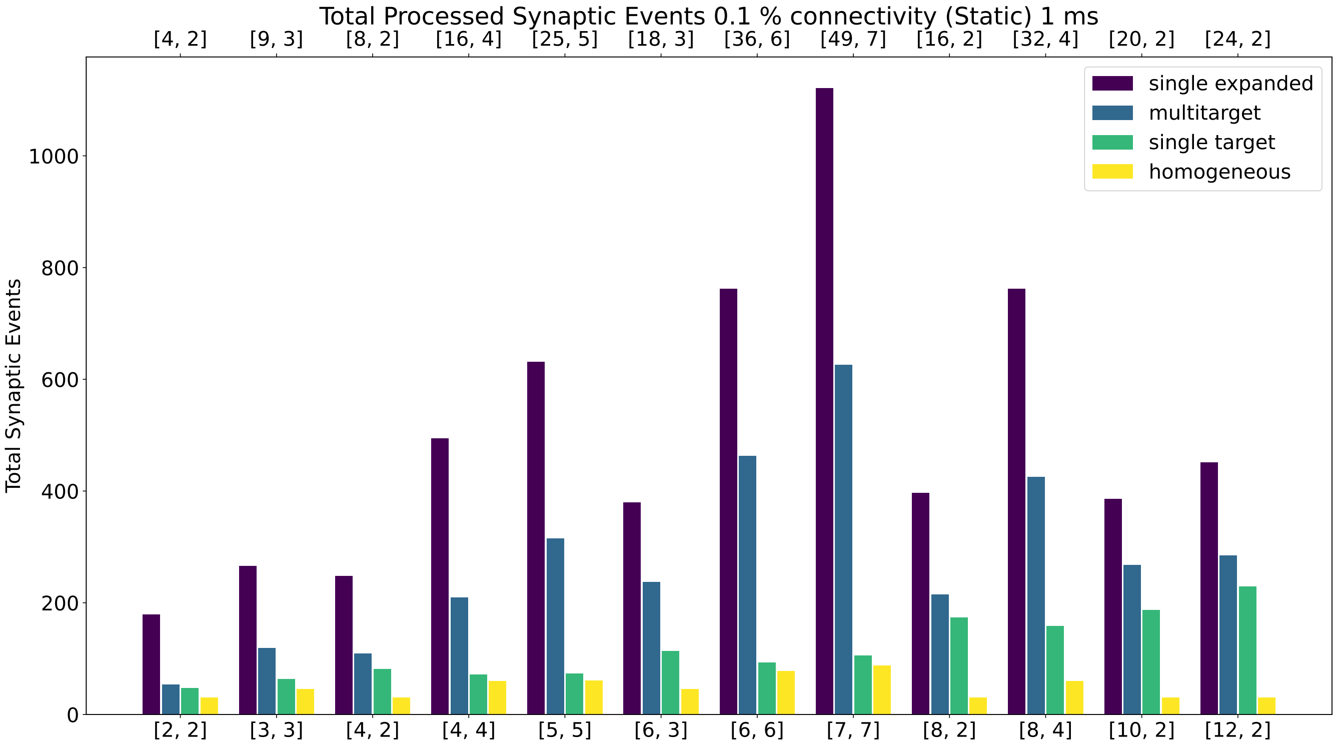

Figure 6. Processed synaptic events per timestep, using 1 ms timestep resolution using 64 neurons per core. Connectivity probability set to 0.1%.

2.3 Plastic Profiling

This section extends the analysis of peak throughput performance to incorporate synaptic plasticity for a range of additional connection density levels. Figure 15 shows the results with 0.1% connectivity, Figure 16 for 5% connectivity, and Figure 17 for 10%.

Similarly to the plastic case presented in the main paper, the Multi-target approach represents the optimal method for a given allocation of resources in all cases. In the case of extreme sparsity (0.1% connectivity), the Multi-target approach even performs better than the extended Heterogeneous configuration. This is explained by the additional operations required by synaptic plasticity. Synaptic rows need to be read from shared memory, updated, and then written back (to commit weight changes). Having larger rows allows to amortise the read and write operations over a higher number of synapses, which in turn requires a reduced number of shared memory accesses. This is amplified by the sparsity, as having longer rows allows to have fewer meaningless accesses to memory and to amortise the read and write back over more synapses. As expected, this effect decreases with increasing density, as rows contain more synapses and therefore the computational power of the extended Heterogeneous approach grants higher processing capability.

2.4 Performance and Resource Comparison

Finally we present the results comparing resource allocation and peak performance for all considered connectivity patterns. These results are shown in form of scatter plots, using the same convention adopted in the main material, but here including case labels. The static values are presented in Figure 18 and 19 for 0.1% connectivity using 0.1 ms and 1 ms timestep resolutions respectively, Figure 22 and 23 for 5%, and Figure 24 and 25 for 10%. Figures 25 and 21 show the labelled 1% connectivity experiments from the main material. For all cases, the single extended case (purple points) processes the highest number of

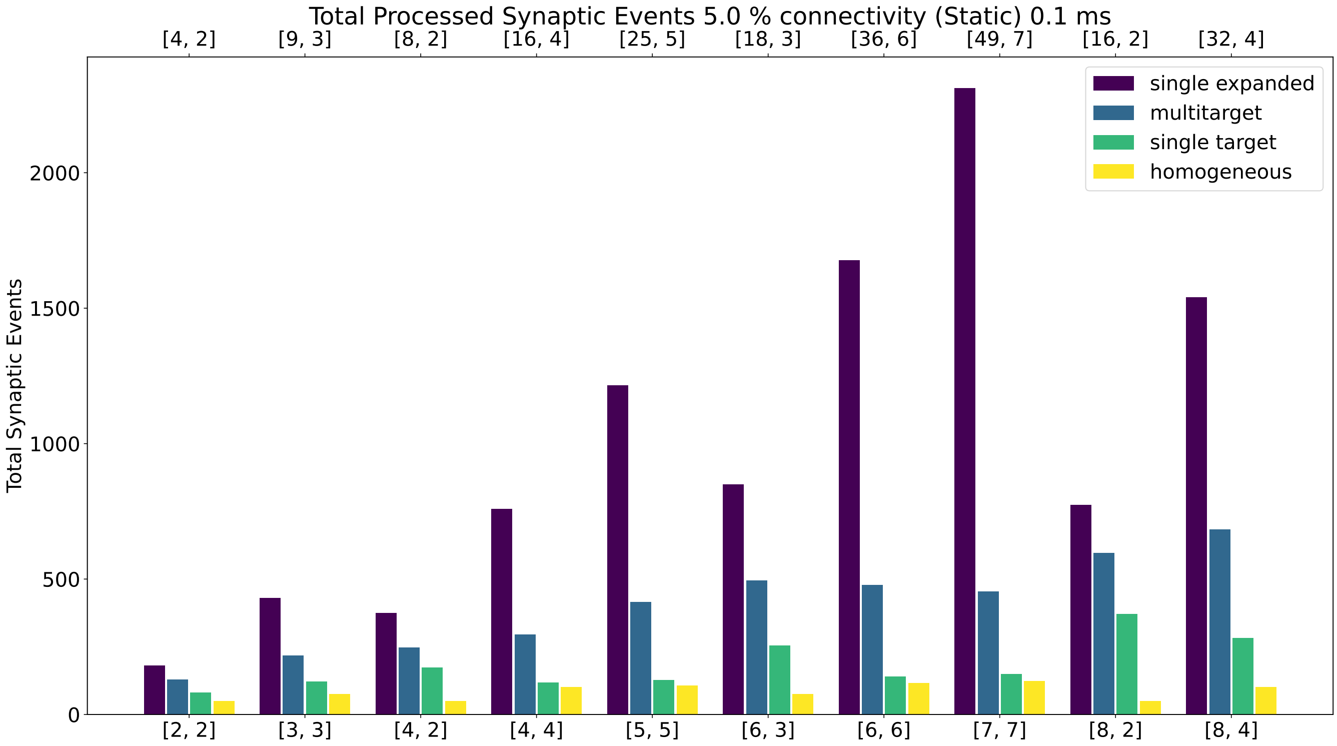

Figure 7. Processed synaptic events per timestep, using 0.1 ms timestep resolution using 64 neurons per core. Connectivity probability set to 5%.

synaptic events, but also requires the largest allocation of hardware resources. The optimal solution remains the Multi-target partitioning (blue points), showing the steepest increases in processing performance with additional resources, with the maximum processed synaptic events close to the single target expanded for all 1 ms simulations, and still optimal for the 0.1 ms. Discrepancies between the Heterogeneous extended and the Multi-target peak performance are due to timing constraints having a greater effect on neural processing, as detailed in the main material. Higher connectivity probabilities result in higher numbers of synaptic events processed per timestep, but flatter slopes in these plots. This can be explained by the increasing number of synapses that each spike targets, which results in a higher processing time per spike packet, combined to a higher transfer time required by the longer synaptic rows.

The plastic values are presented in Figure 26 for 0.1%, Figure 27 for 1%, Figure 28 for 5% and Figure 29 for 10% connectivity. The presented results follow a trend similar to the static case, however, as detailed in the barchart analysis for peak performance, the case of very sparse connectivity shows the Multi-target approach dominating all the other solutions.

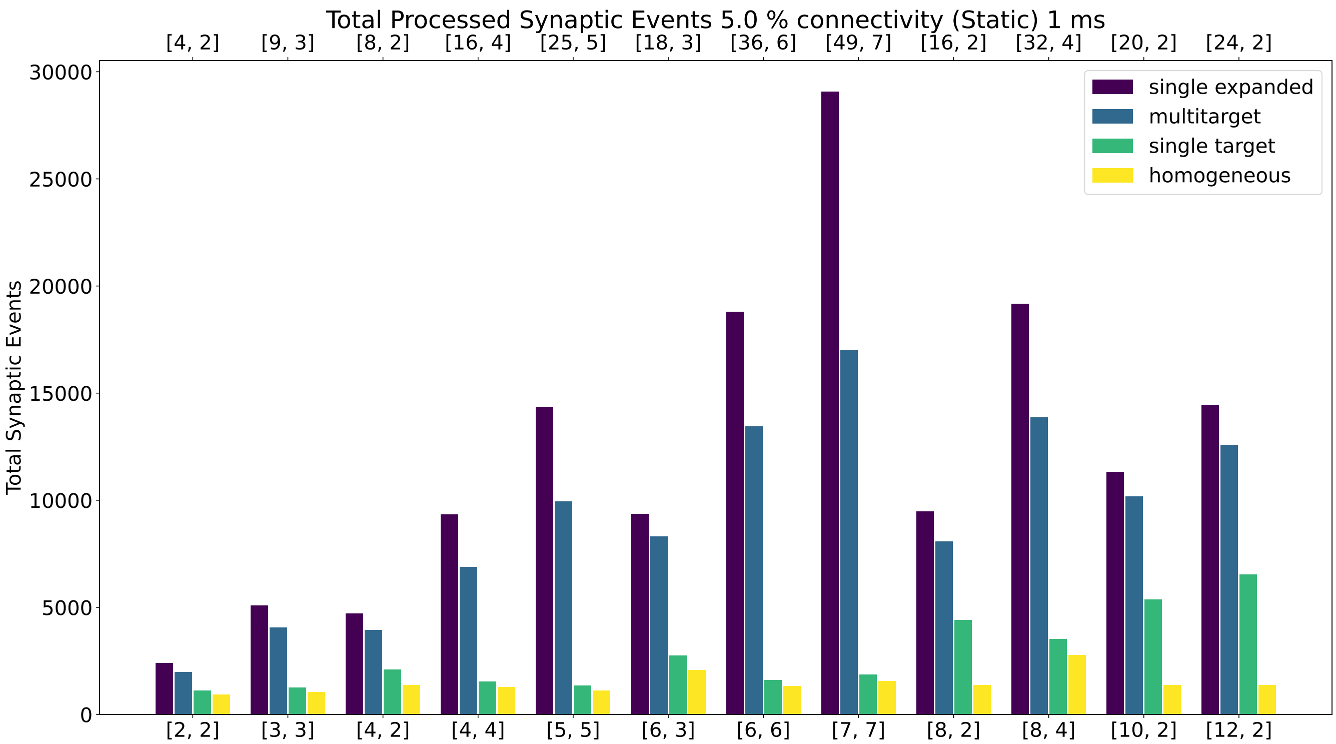

Figure 8. Processed synaptic events per timestep, using 1 ms timestep resolution. using 64 neurons per core Connectivity probability set to 5%.

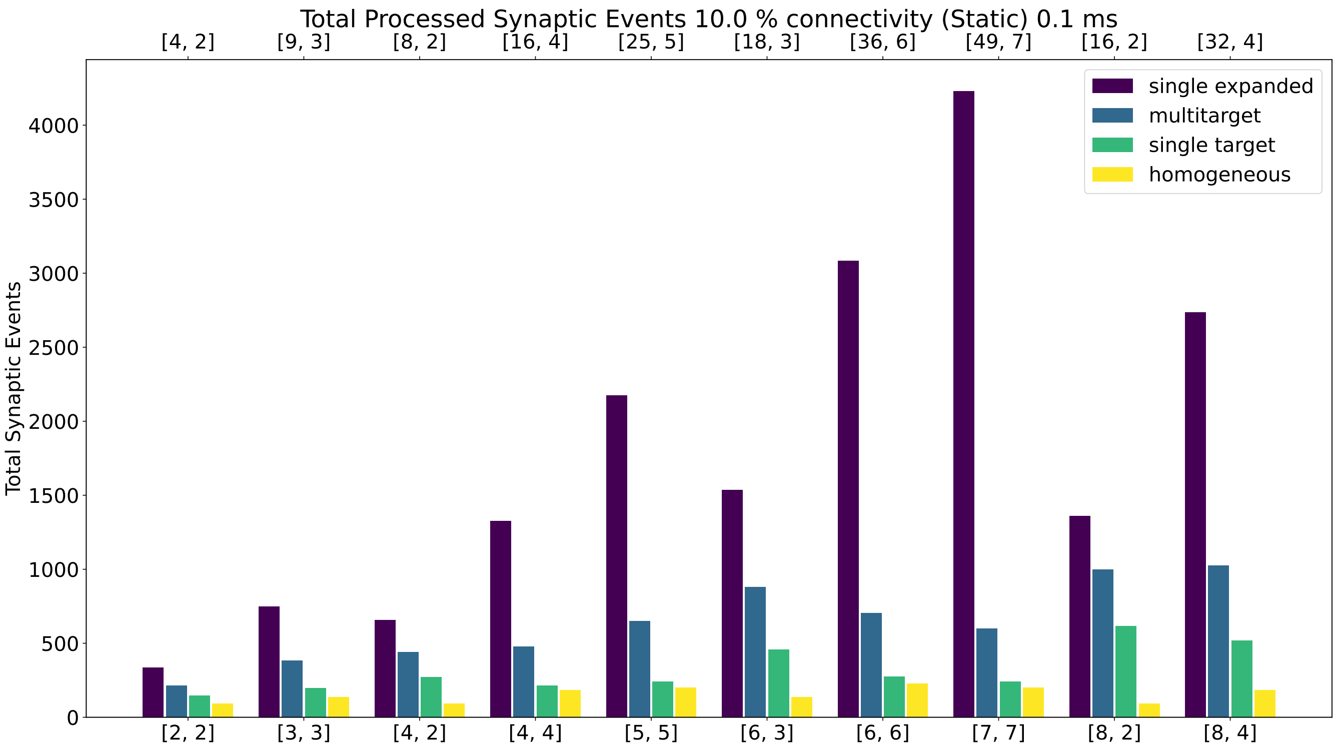

Figure 9. Processed synaptic events per timestep, using 0.11 ms timestep resolution using 64 neurons per core. Connectivity probability set to 10%.

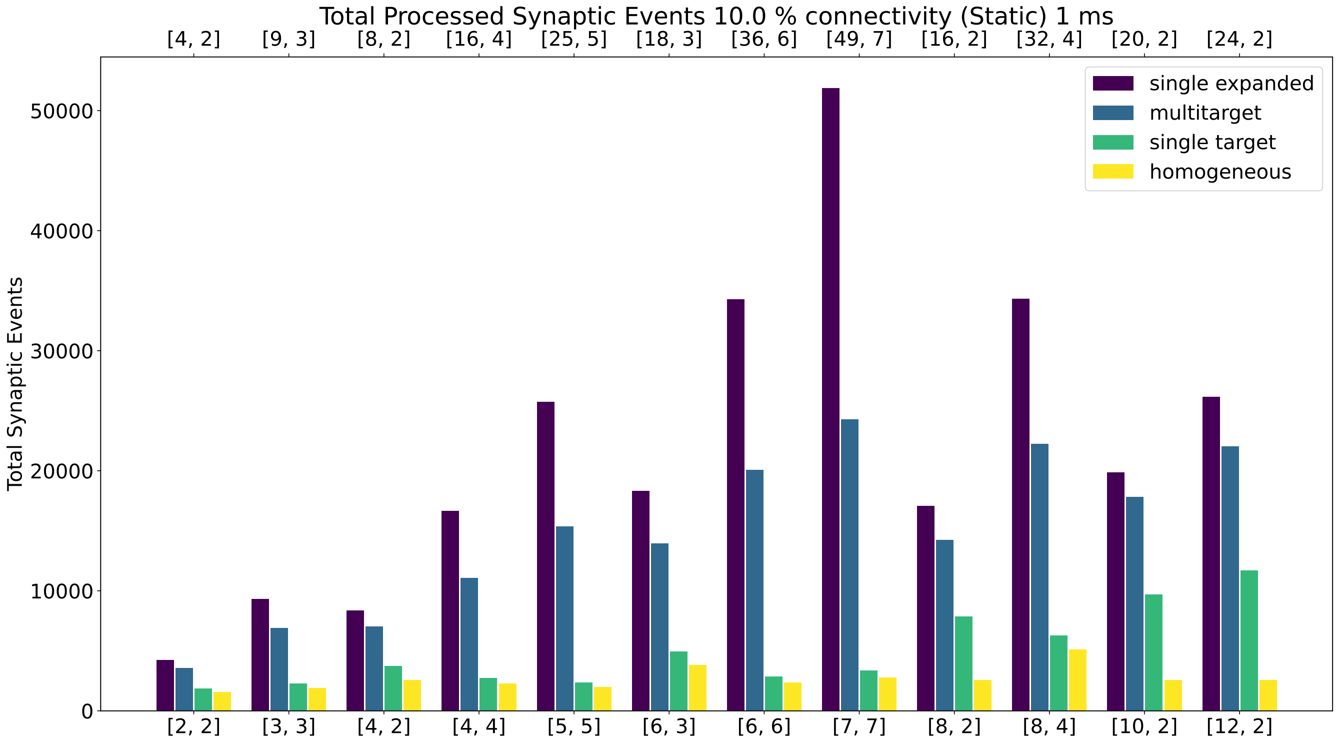

Figure 10. Processed synaptic events per timestep, using 1 ms timestep resolution using 64 neurons per core. Connectivity probability set to 10%.

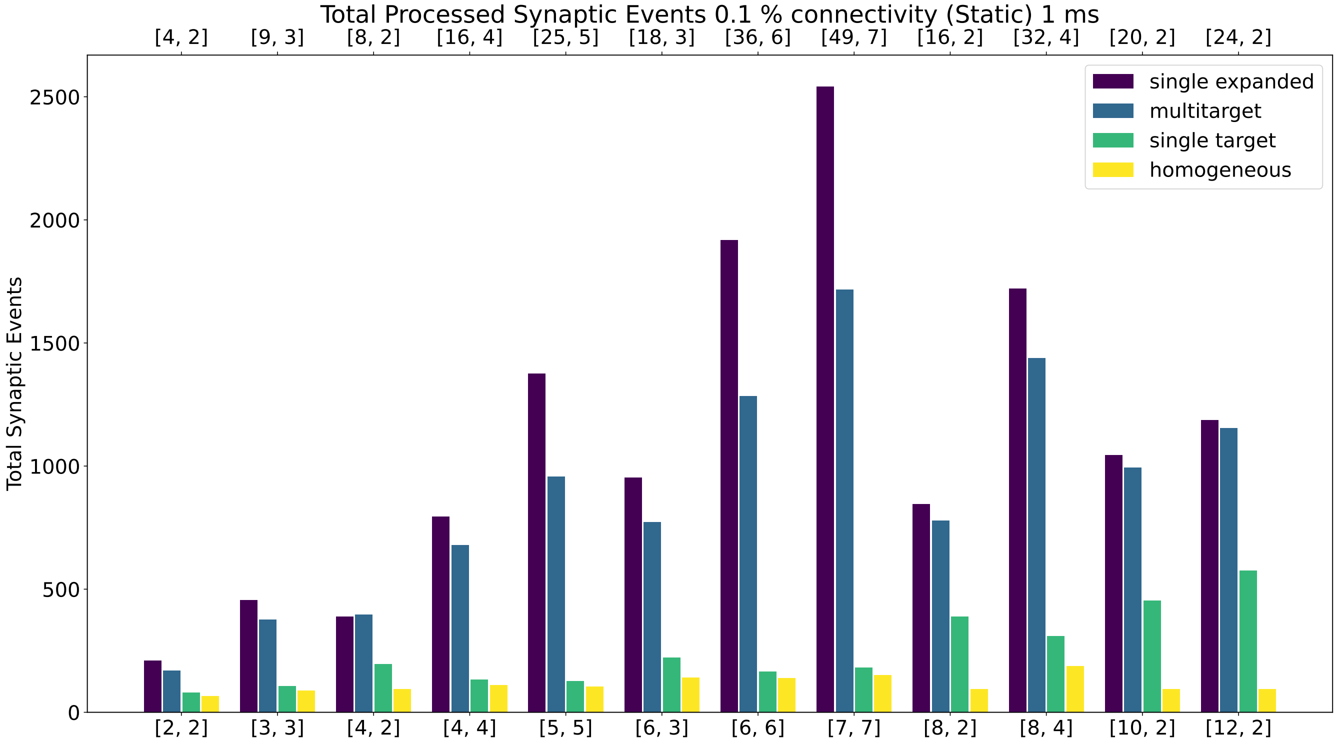

Figure 11. Processed synaptic events per timestep, using 1 ms timestep resolution using 256 neurons per core. Connectivity probability set to 0.1%.

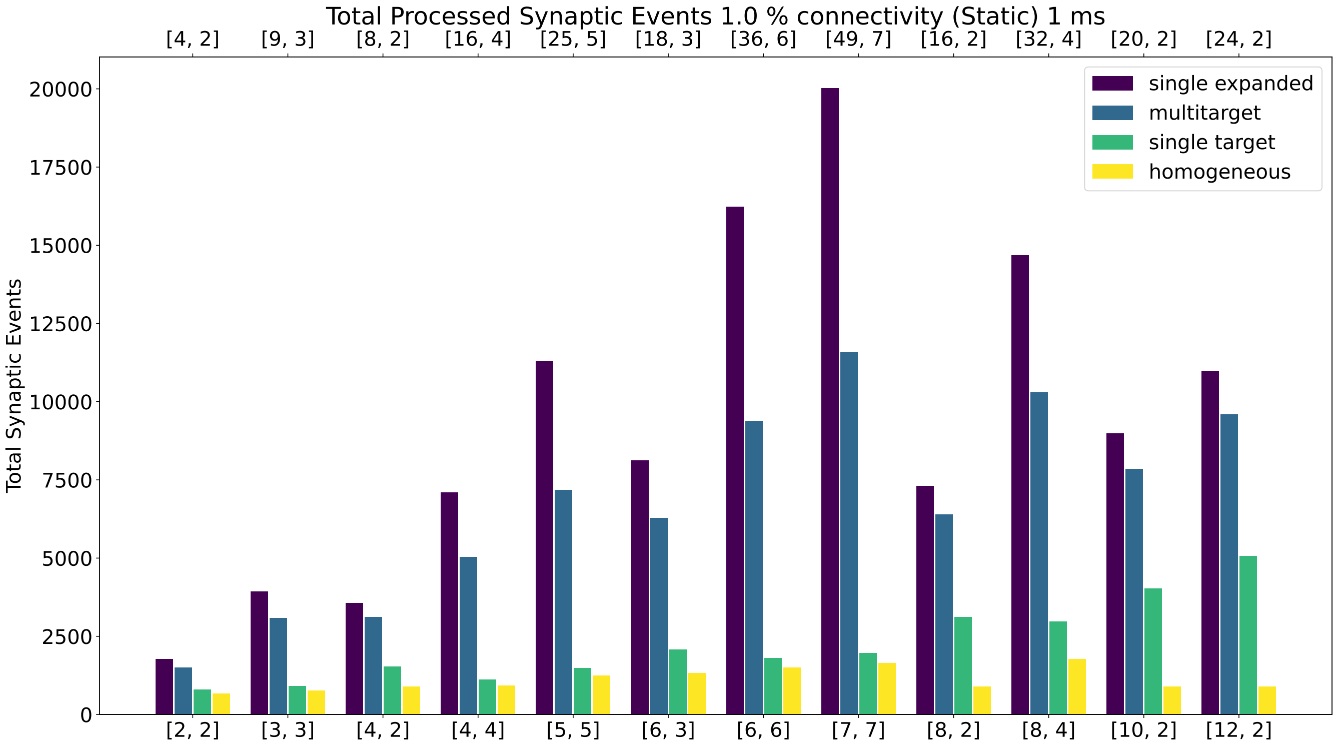

Figure 12. Processed synaptic events per timestep, using 1 ms timestep resolution using 256 neurons per core. Connectivity probability set to 1%.

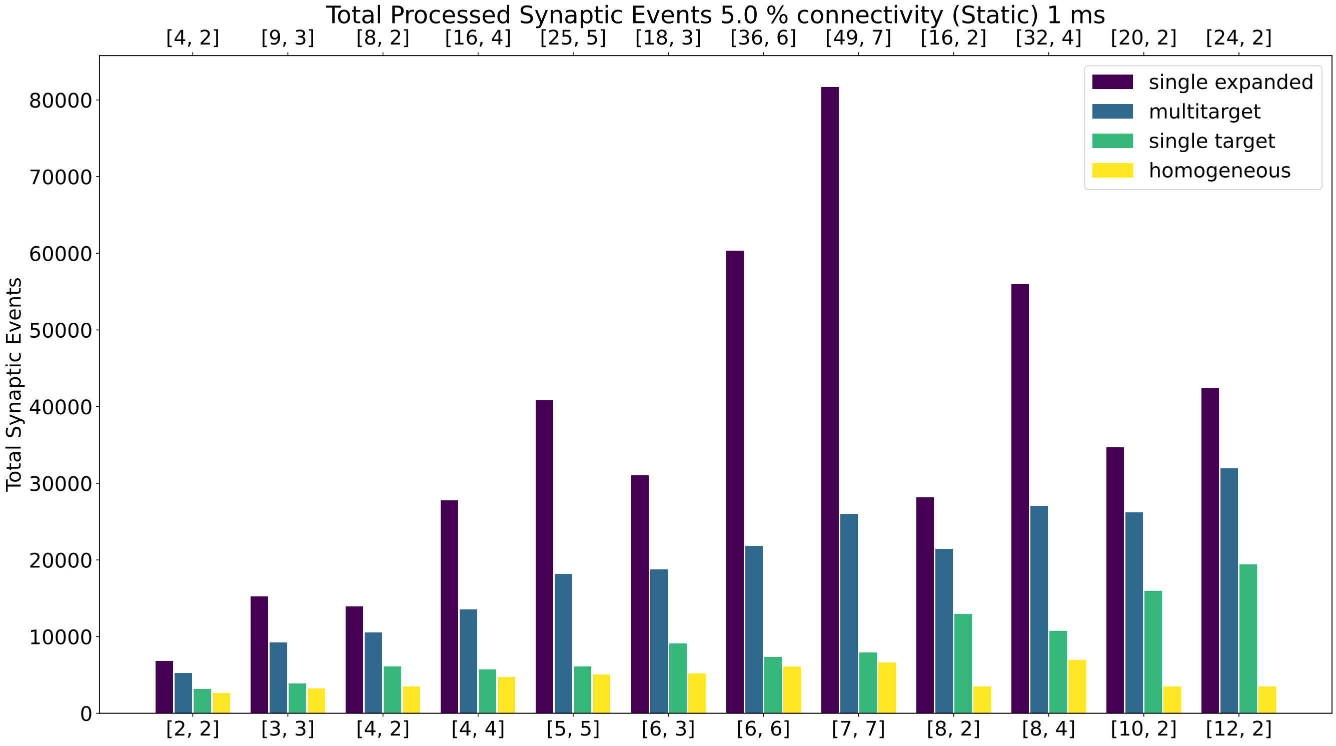

Figure 13. Processed synaptic events per timestep, using 1 ms timestep resolution using 256 neurons per core. Connectivity probability set to 5%.

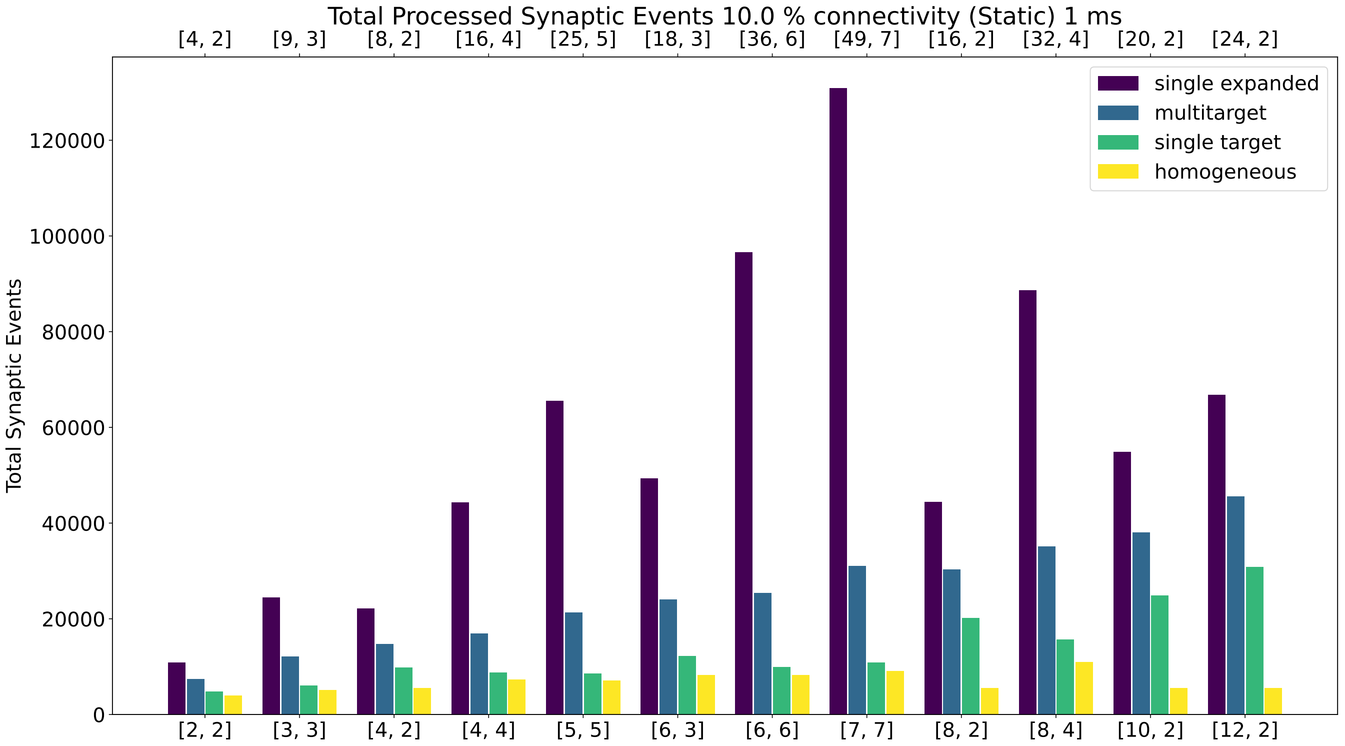

Figure 14. Processed synaptic events per timestep, using 1 ms timestep resolution using 256 neurons per core. Connectivity probability set to 10%.

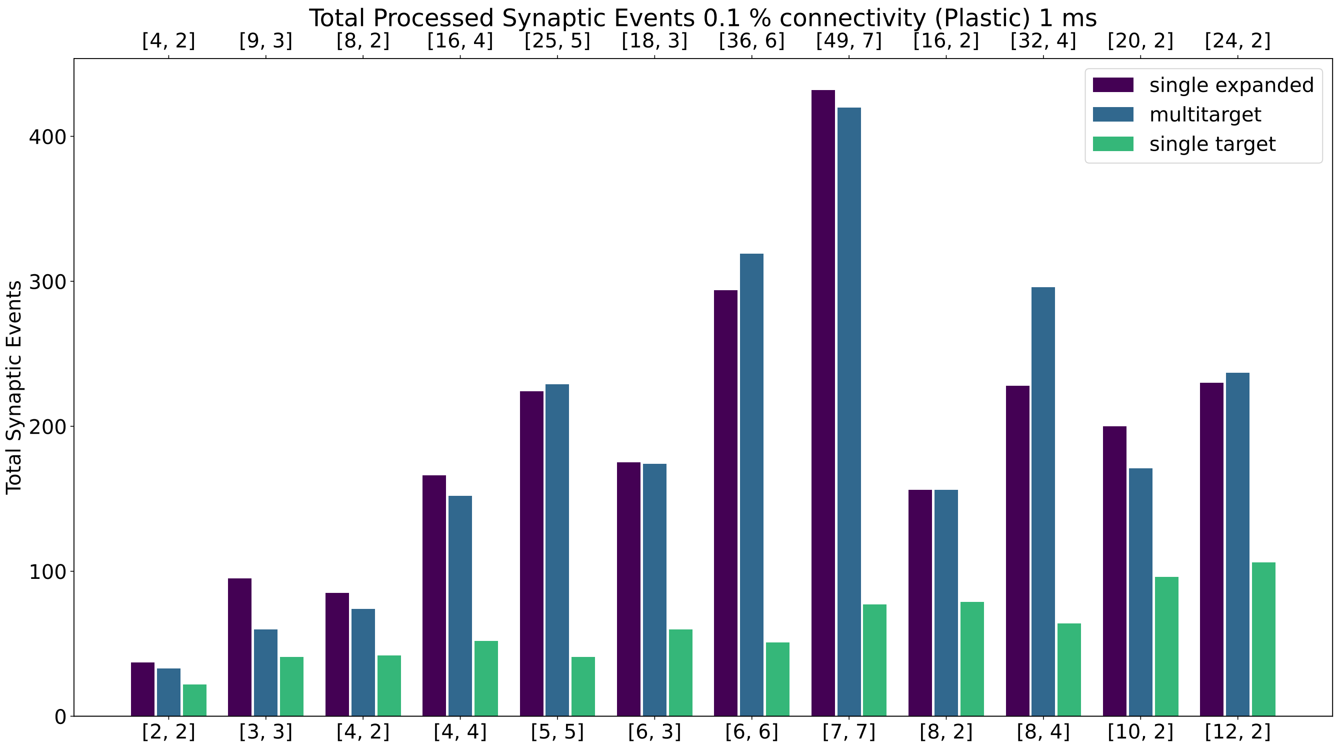

Figure 15. Processed synaptic events per timestep. Plastic configuration. Connectivity probability set to 0.1%

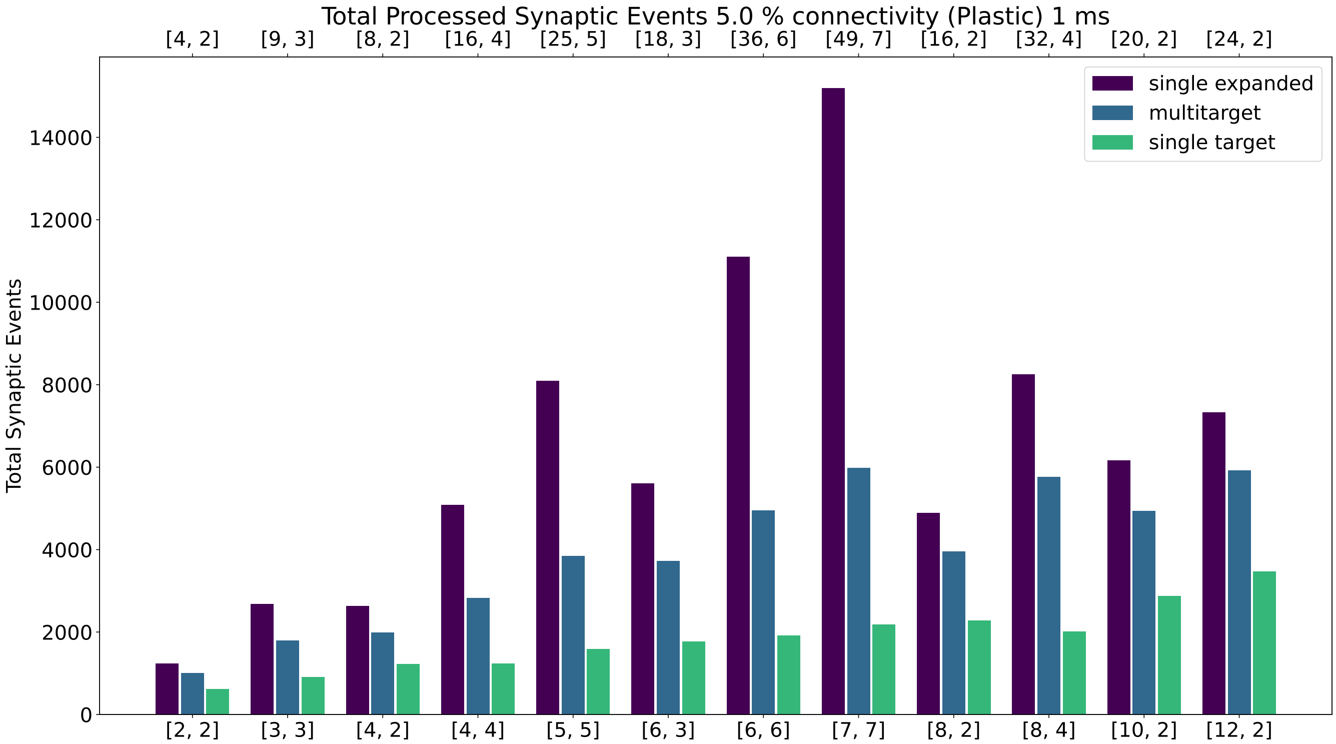

Figure 16. Processed synaptic events per timestep. Plastic configuration. Connectivity probability set to 5%

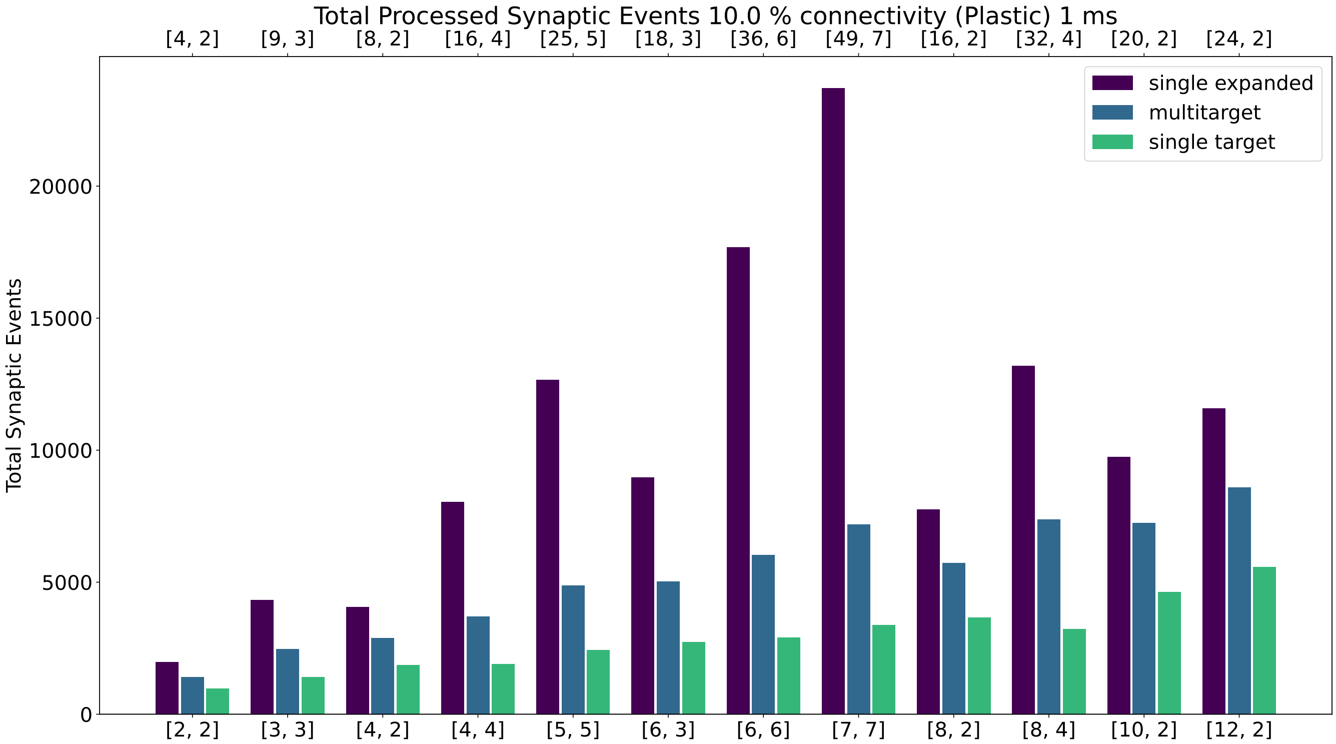

Figure 17. Processed synaptic events per timestep. Plastic configuration. Connectivity probability set to 10%

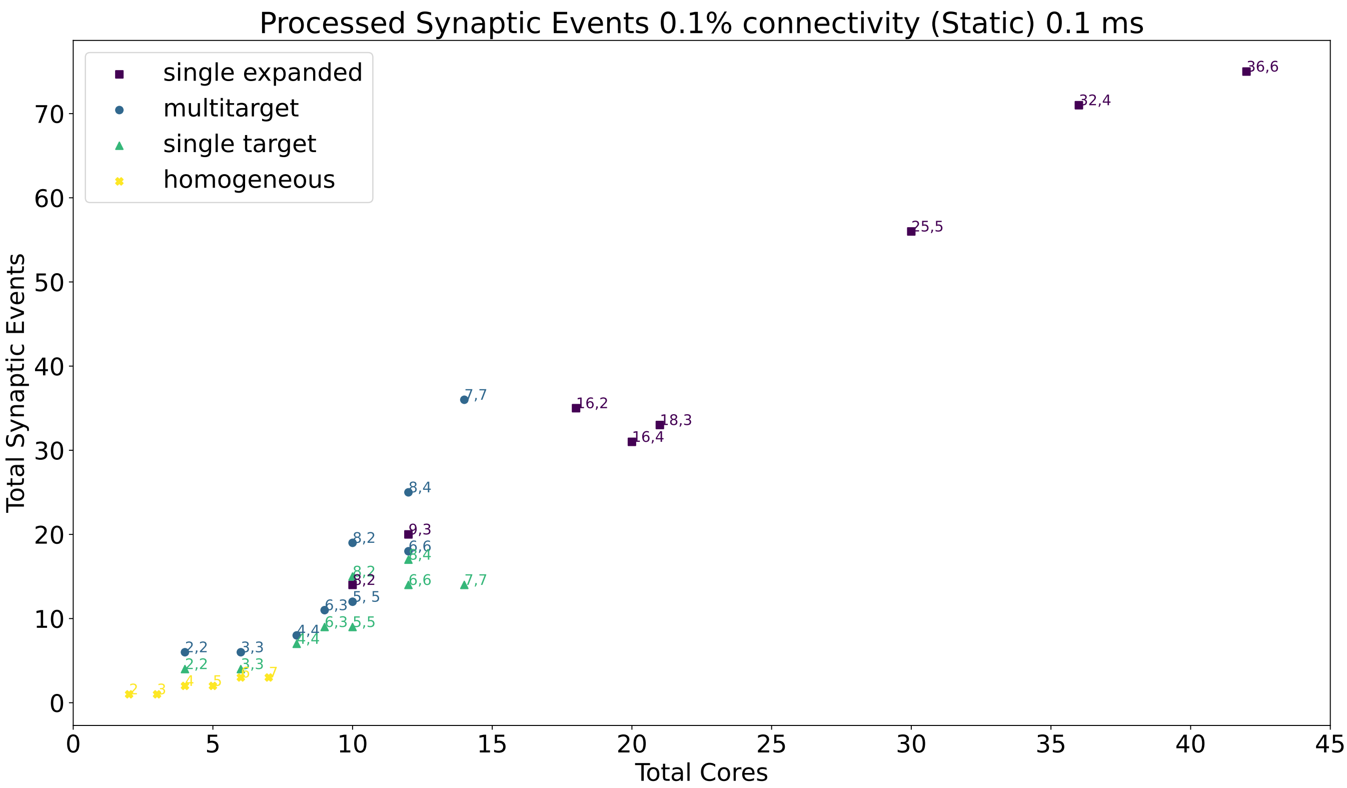

Figure 18. Resource allocation vs peak performance for the three different approaches using 0.1 ms timestep. Connectivity probability set to 0.1%

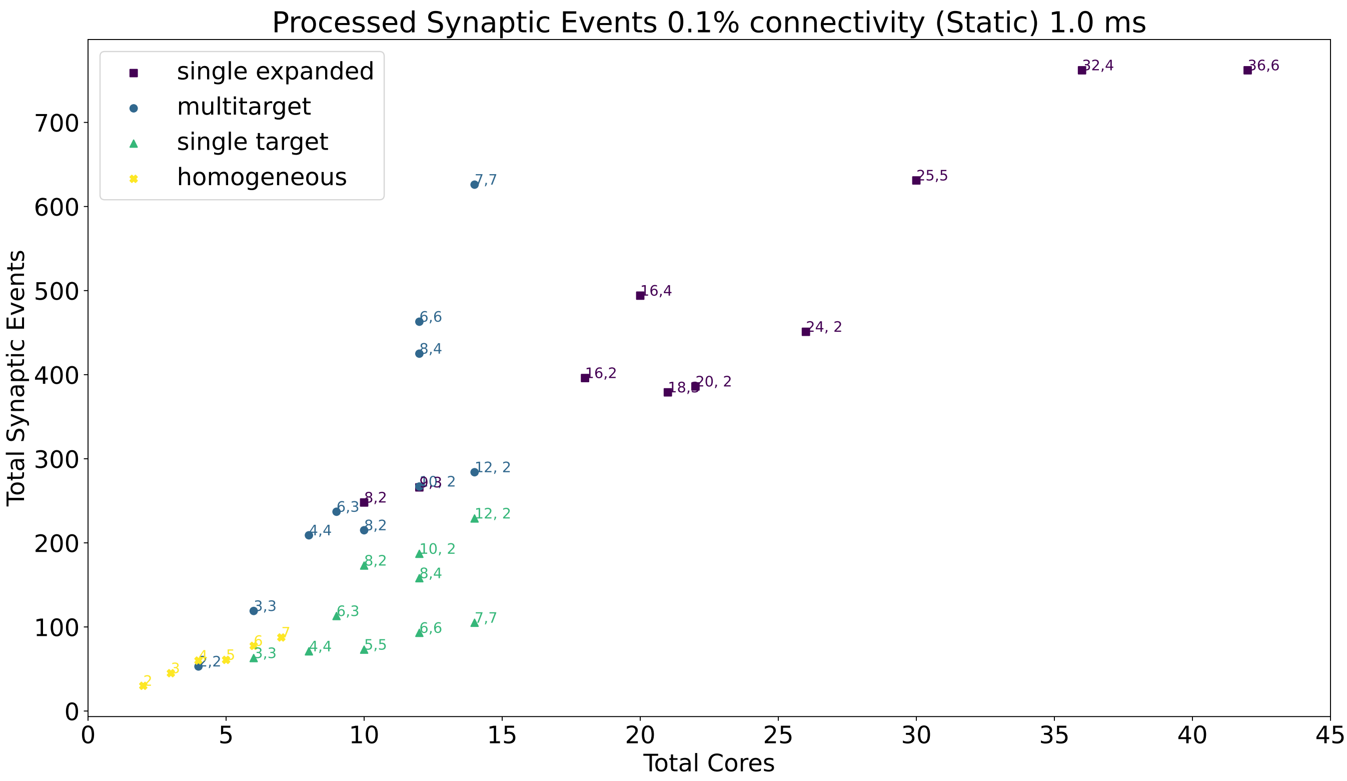

Figure 19. Resource allocation vs peak performance for the three different approaches using 1 ms timestep. Connectivity probability set to 0.1%

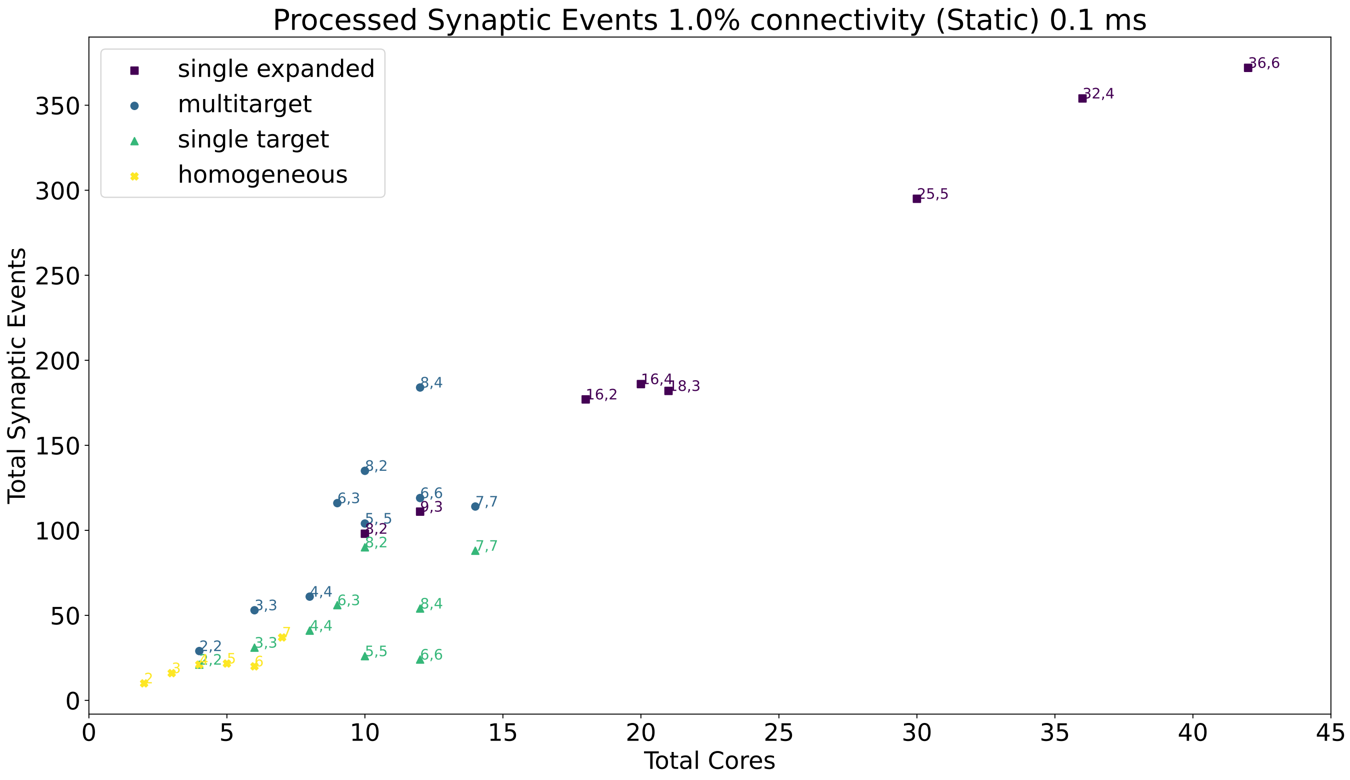

Figure 20. Resource allocation vs peak performance for the three different approaches using 0.1 ms timestep. Connectivity probability set to 1%

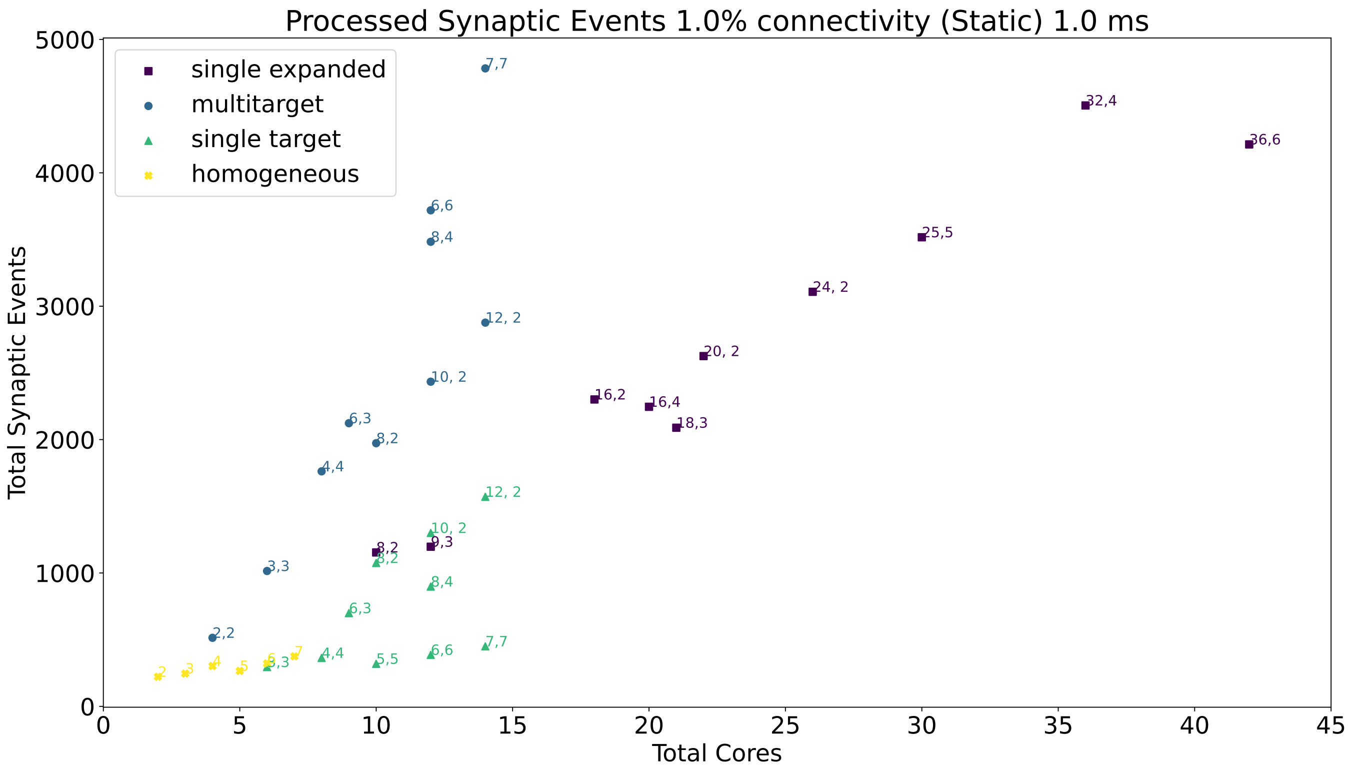

Figure 21. Resource allocation vs peak performance for the three different approaches using 1 ms timestep. Connectivity probability set to 1%

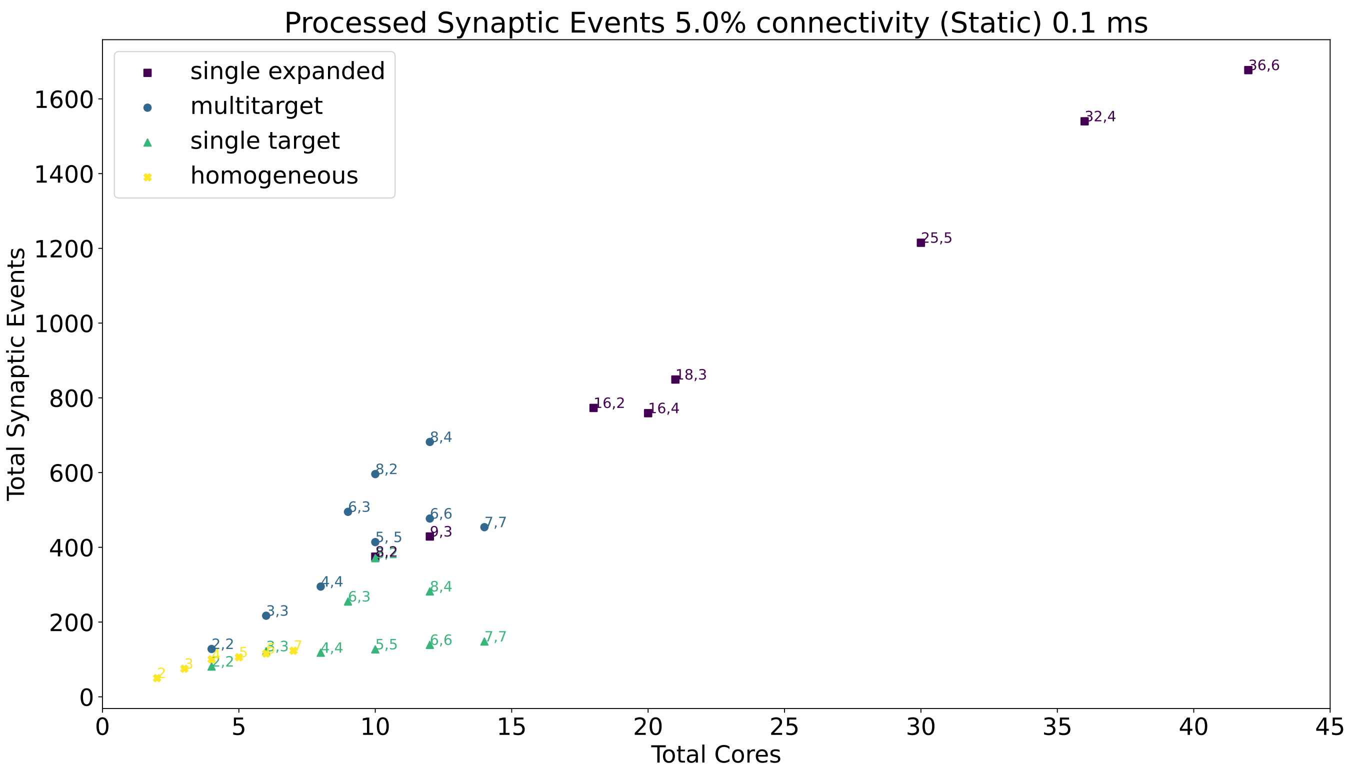

Figure 22. Resource allocation vs peak performance for the three different approaches using 0.1 ms timestep. Connectivity probability set to 5%

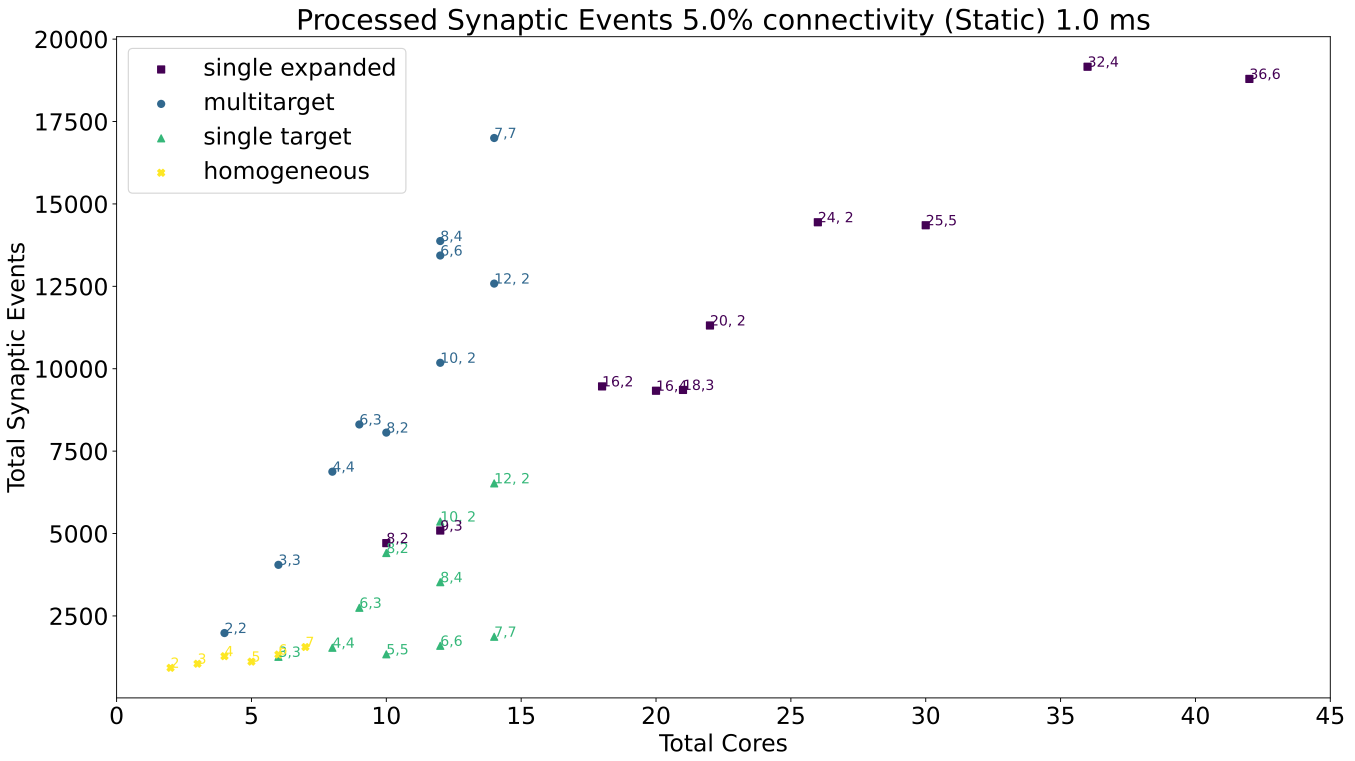

Figure 23. Resource allocation vs peak performance for the three different approaches using 1 ms timestep. Connectivity probability set to 5%

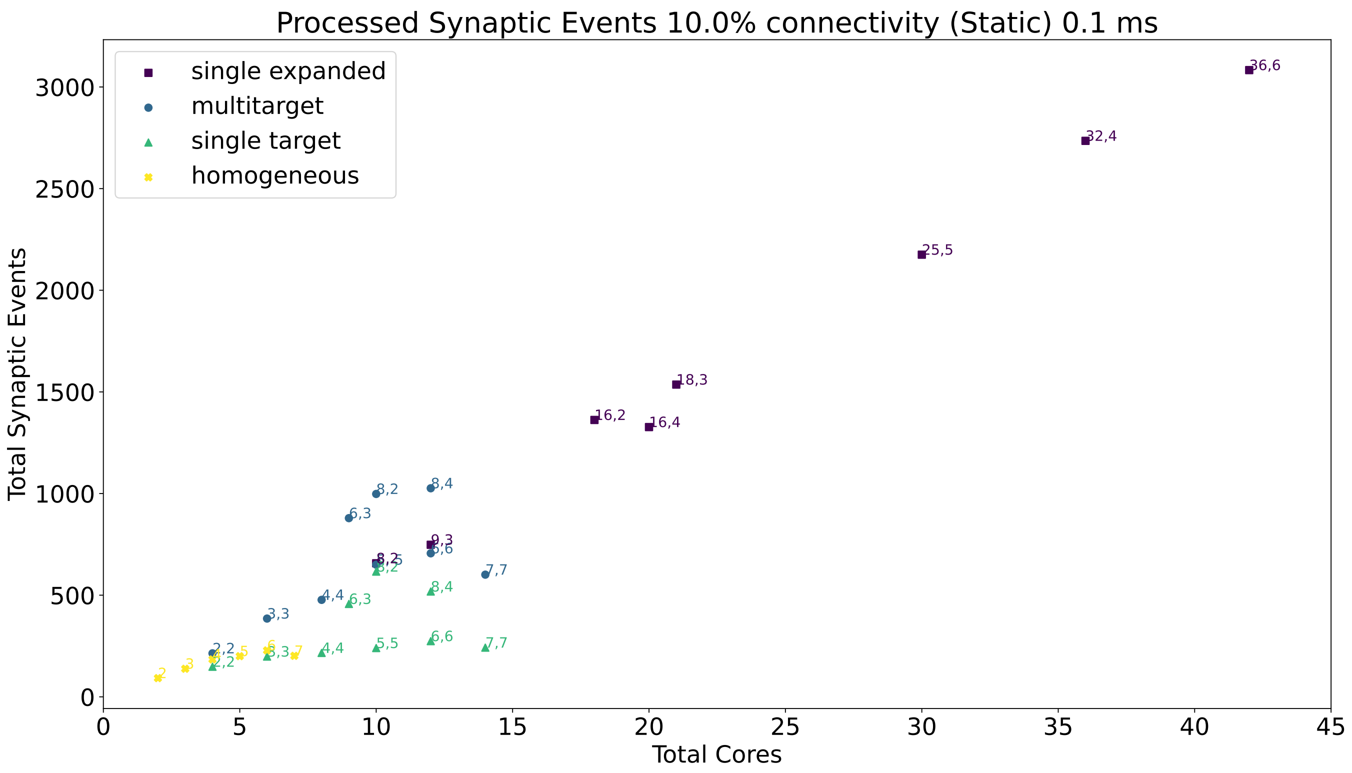

Figure 24. Resource allocation vs peak performance for the three different approaches using 0.1 ms timestep. Connectivity probability set to 10%

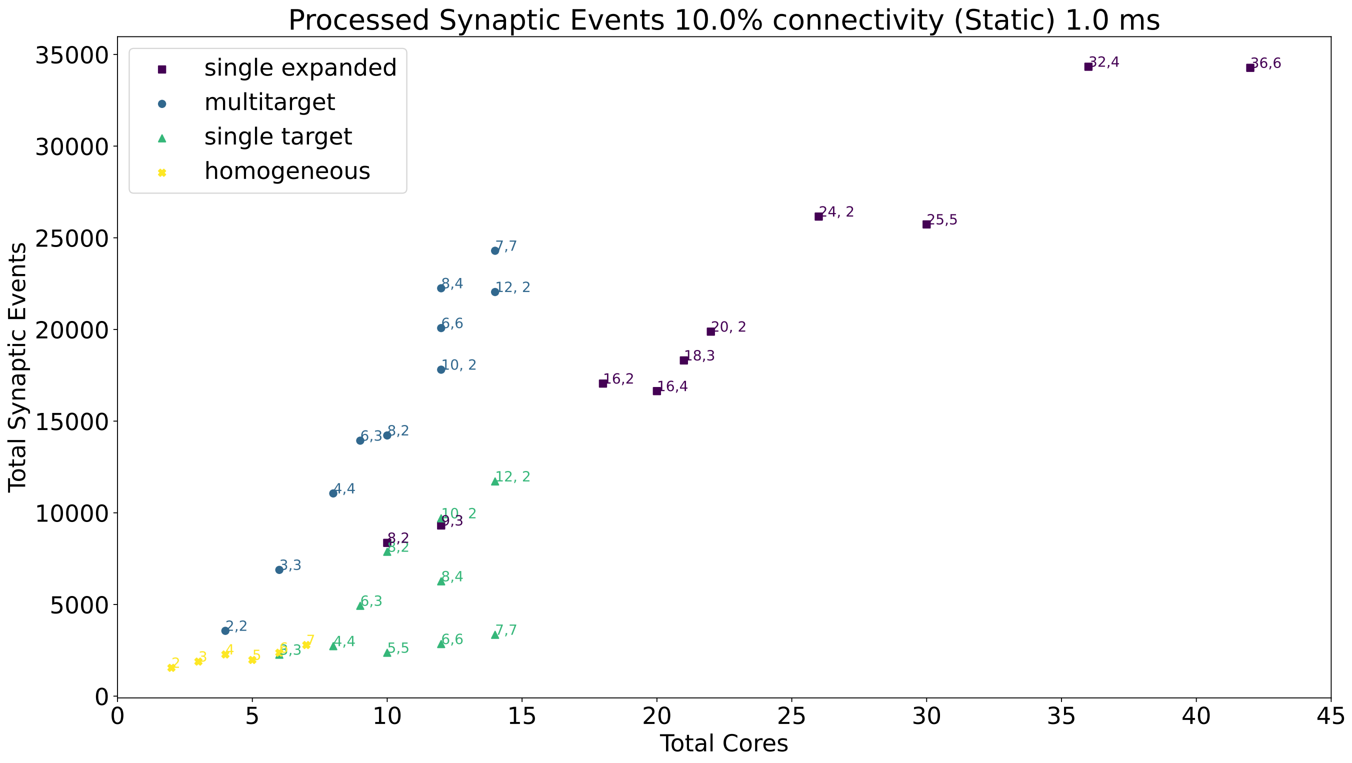

Figure 25. Resource allocation vs peak performance for the three different approaches using 1 ms timestep. Connectivity probability set to 10%

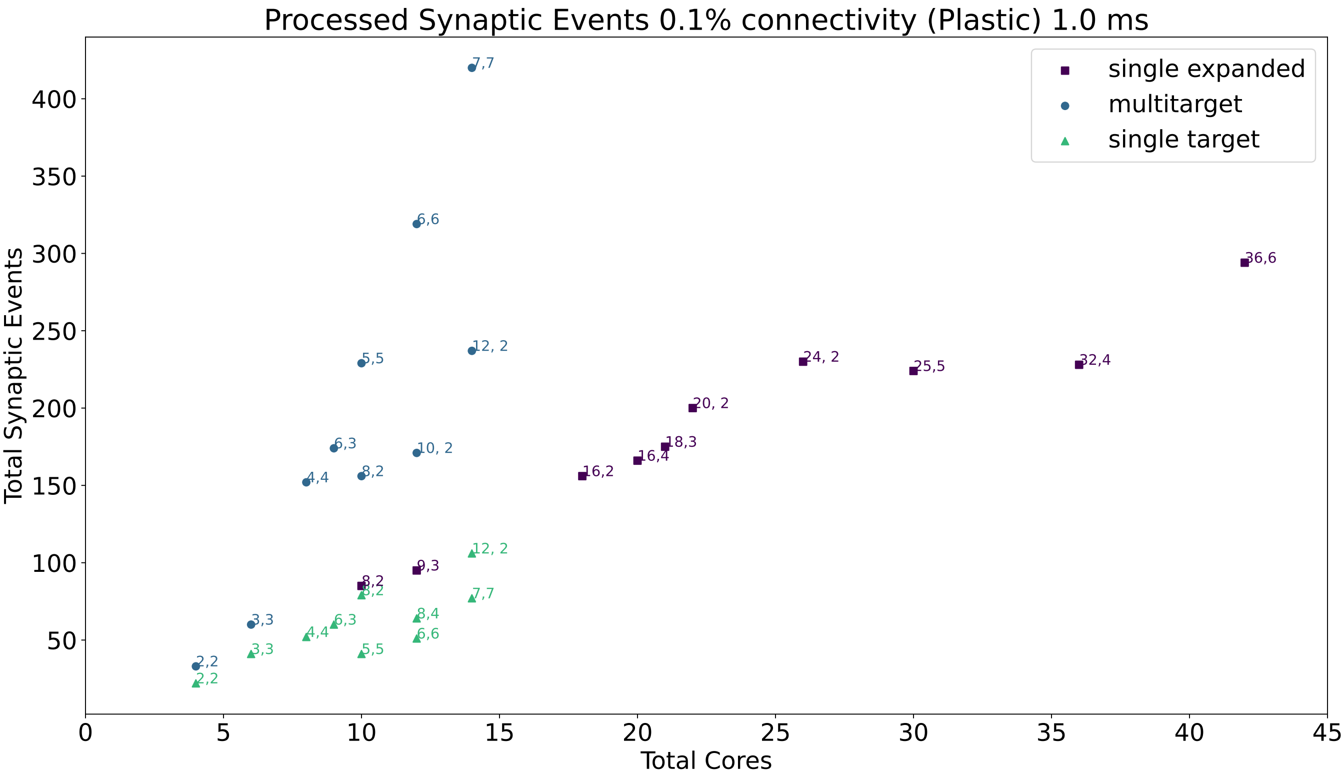

Figure 26. Resource allocation vs peak performance for the three different approaches. Plastic configuration. Connectivity probability set to 0.1%

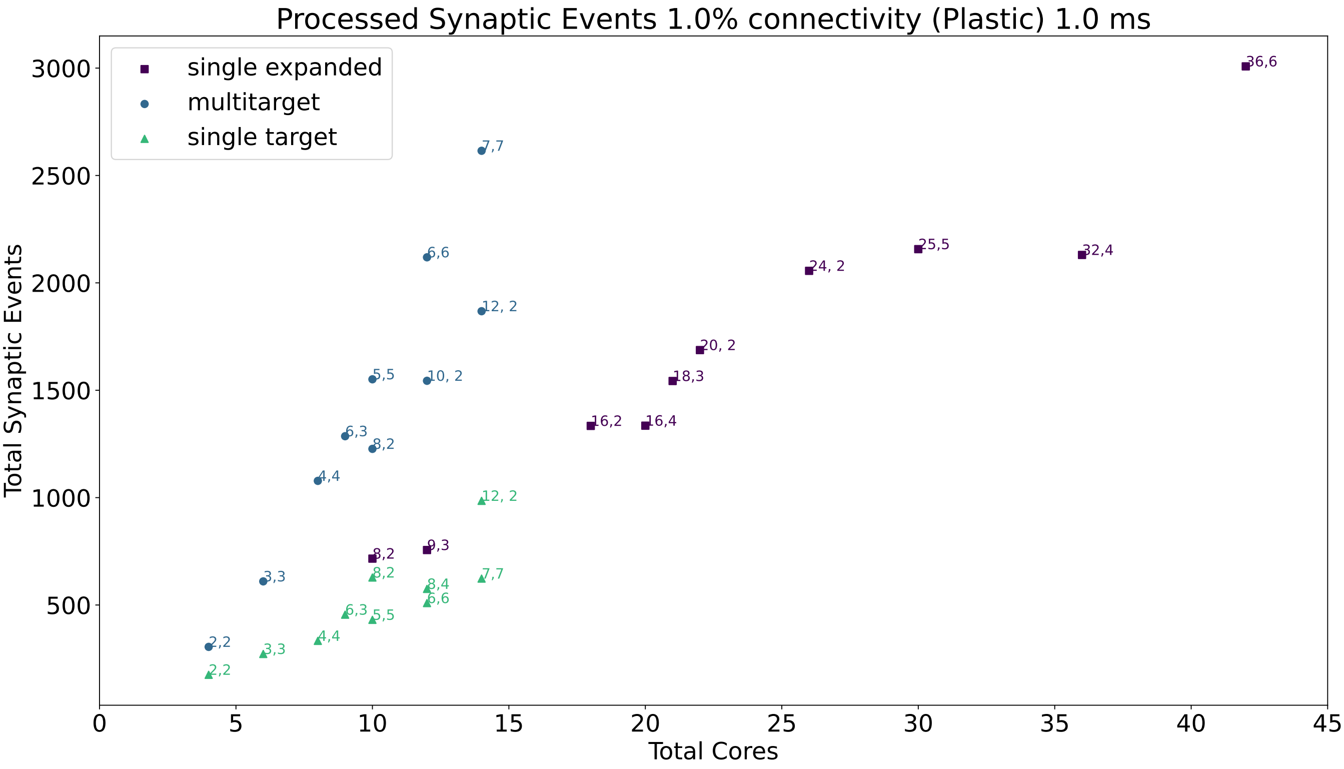

Figure 27. Resource allocation vs peak performance for the three different approaches. Plastic configuration. Connectivity probability set to 1%

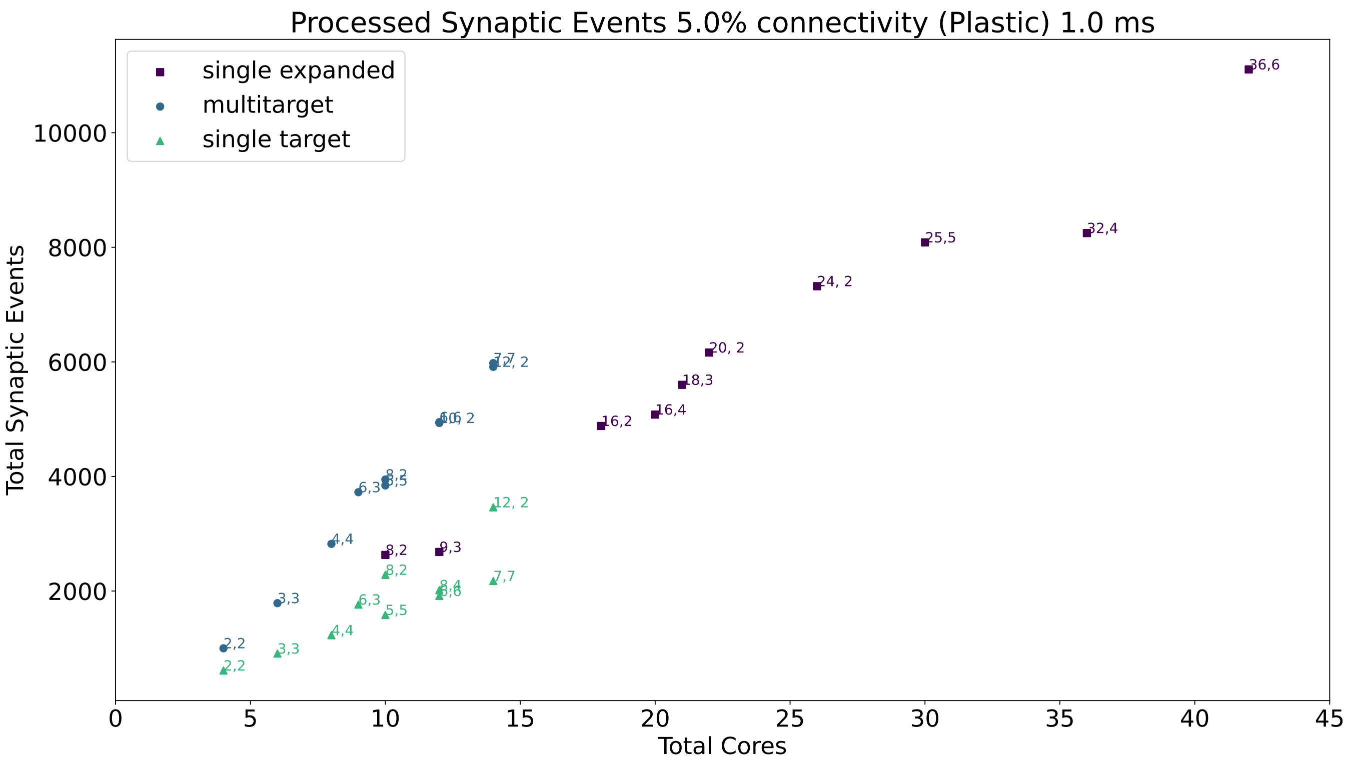

Figure 28. Resource allocation vs peak performance for the three different approaches. Plastic configuration. Connectivity probability set to 5%

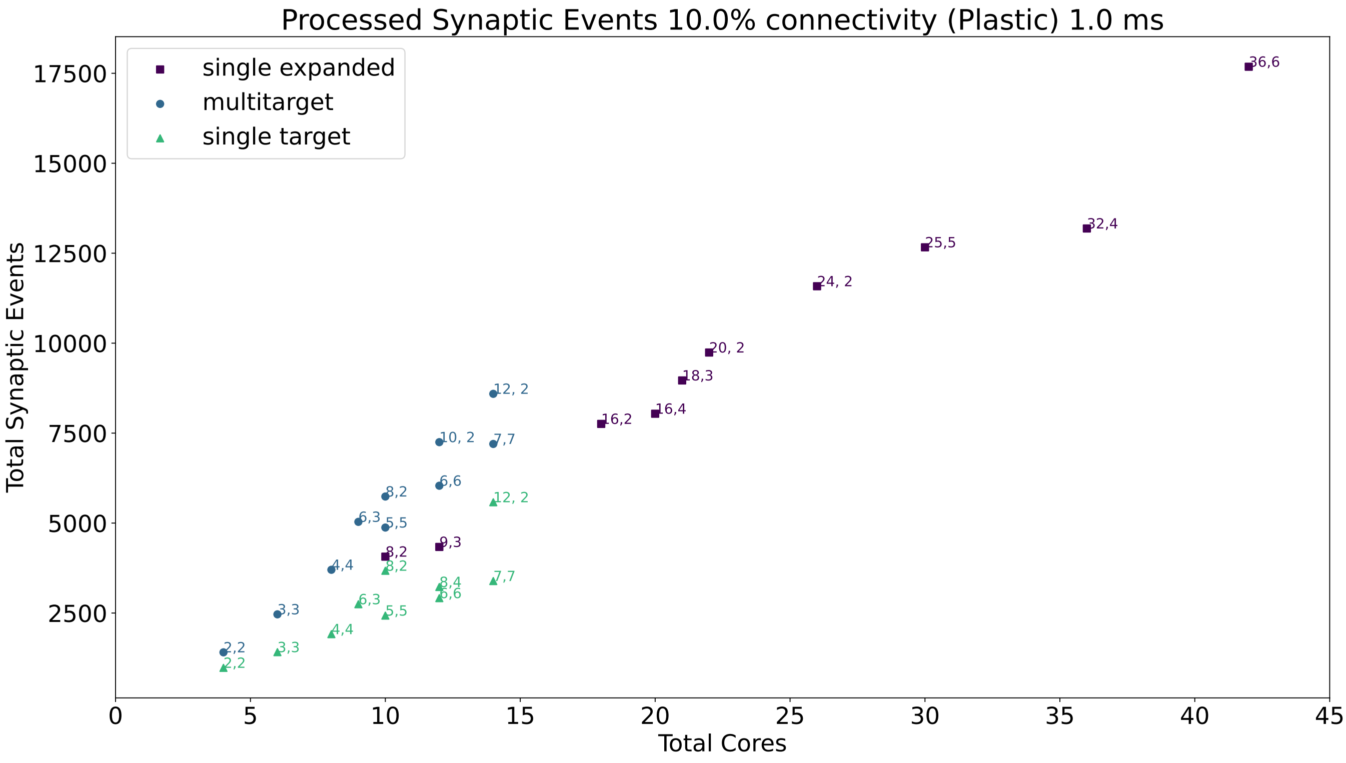

Figure 29. Resource allocation vs peak performance for the three different approaches. Plastic configuration. Connectivity probability set to 10%
